# Use of sodium valproate and other antiseizure drug treatments in England and Wales: quantitative analysis of nationwide linked electronic health records

**DOI:** 10.1136/bmjmed-2023-000760

**Published:** 2024-12-20

**Authors:** Caroline E Dale, Rohan Takhar, Yat Yi Fan, Fatemeh Torabi, Michail Katsoulis, Samuel Kim, Andrew Lambarth, Christopher Tomlinson, Tim Wilkinson, Tanja Mueller, Amanj Kurdi, Mark Ashworth, Mamas A Mamas, Kamlesh Khunti, Ashley Akbari, Andrew D Morris, Munir Pirmohamed, Anthony G Marson, David Williams, David Hunt, Cathie Sudlow, Reecha Sofat

**Affiliations:** 1 Department of Pharmacology and Therapeutics, University of Liverpool, Liverpool, UK; 2 Swansea University Medical School, Swansea, UK; 3 Medical Research Council (MRC) Unit for Lifelong Health and Ageing, London, London, UK; 4 Royal Free London NHS Foundation Trust, London, UK; 5 St George's University Hospitals NHS Foundation Trust, London, UK; 6 University College London Institute of Health Informatics, London, UK; 7 University of Edinburgh Centre for Clinical Brain Sciences, Edinburgh, UK; 8 Strathclyde Institute of Pharmacy and Biomedical Sciences, Glasgow, UK; 9 Department of Clinical Pharmacy, College of Pharmacy, Hawler Medical University, Erbil, Kurdistan, Iraq; 10 Al-Kitab University, Kirkuk, Iraq; 11 King's College London School of Life Course and Population Sciences, London, UK; 12 Keele University Faculty of Medicine and Health Sciences, Keele, UK; 13 University of Leicester Diabetes Research Centre, Leicester, UK; 14 Health Data Research UK, London, UK; 15 Walton Centre NHS Foundation Trust, Liverpool, UK; 16 University College London Elizabeth Garrett Anderson (UCL EGA) Institute for Women's Health, London, UK; 17 British Heart Foundation Data Science Centre, Health Data Research UK, London, UK

**Keywords:** Neurology, Pharmacology, Prenatal care, Medical informatics

## Abstract

**Objective:**

To investigate the use of sodium valproate in England and Wales, including during pregnancy, compared with other antiseizure drug treatments, based on national level electronic health records.

**Design:**

Quantitative analysis of nationwide linked electronic health records.

**Setting:**

Individual level, population scale data from NHS England's Secure Data Environment, from the British Heart Foundation Data Science Centre's CVD-COVID-UK/COVID-IMPACT Consortium (for England), and the Secure Anonymised Information Linkage Databank (for Wales), 1 January 2019 to 31 December 2023.

**Participants:**

1 200 000 individuals dispensed any selected antiseizure drug treatment (ie, sodium valproate, lamotrigine, levetiracetam, carbamazepine, or topiramate); 304 000 women, aged 15-49 years, dispensed any selected antiseizure drug treatment and 28 400 women, aged 15-49 years, dispensed sodium valproate.

**Main outcome measures:**

Prevalent (current) and incident (new) uses of sodium valproate and other antiseizure drug treatments before and during the covid-19 pandemic (1 January 2019 to 31 December 2023), grouped by age and sex. Pregnancy rates per 1000 women, aged 15-49 years, who used antiseizure drug treatments, and timing and dose of sodium valproate dispensed during pregnancy. Geographical variation in use of sodium valproate and disease indications (epilepsy and bipolar affective disorder). Trends in deaths related to epilepsy for 2015-22.

**Results:**

Prevalent use of sodium valproate in women of childbearing potential decreased and use of most other antiseizure drug treatments increased between 2019 and 2023. Incident use of sodium valproate per 100 000 women decreased from seven to five in women aged 15-19 years, from 11 to seven in women aged 20-29 years, and from 14 to seven in women aged 30-39 years between 2019 and 2022. Incident use also decreased in men of the same age but remained at much higher levels (from 53 to 43 in men aged 15-19 years, 59 to 47 in men aged 20-29 years, and 57 to 42 in men aged 30-39 years, per 100 000 men). Pregnancy rates decreased from 6.0 to 5.2 per 1000 women of childbearing potential who were dispensed sodium valproate over the same period. The number of pregnant women who used sodium valproate during pregnancy decreased from 140 in 2019 to 85 in 2023. Epilepsy was the most common indication, followed by bipolar affective disorder (751 and 193 per 1000 women of childbearing potential dispensed sodium valproate, respectively, in 2023). No clear evidence was found that deaths related to epilepsy increased in women aged 15-49 during 2015-22, but a slight increase was found in men aged 15-49 during the later period between April 2018 and December 2022.

**Conclusions:**

Based on comprehensive national records, changes in the dispensing of antiseizure drug treatments in response to regulatory actions were tracked. Rates for use of sodium valproate by women, including during pregnancy, decreased before and continued to slowly decrease during the covid-19 pandemic. Incident use was also reduced in men but remained at much higher levels than in women. This approach, linking national dispensing data to health records at the individual level, could help monitor changes to medicines affected by regulatory changes, including in specific population groups, such as pregnant individuals, and their potential effect on health outcomes.

**WHAT IS ALREADY KNOWN ON THIS TOPIC**

- Sodium valproate is an effective antiseizure drug treatment, but is teratogenic and is associated with a range of adverse neurodevelopmental effects
- Policy changes in 2018 strengthened the regulatory position on sodium valproate, limiting prescriptions in women of childbearing potential and introducing the pregnancy prevention programme
- Existing data are not comprehensive on the use of sodium valproate by women of childbearing potential, including during pregnancy, and on the effect of the covid-19 pandemic on the implementation of policy changes

**WHAT THIS STUDY ADDS**

- Linking information from electronic health record data sources, nationwide pregnancy episodes were identified to look at antiseizure drug treatments during pregnancy, including dose and timing of use
- The study showed that rates of use of sodium valproate by women of childbearing potential, including during pregnancy, decreased before and continued to slowly decrease during the covid-19 pandemic, despite disruption to most services
- No clear evidence was found that epilepsy related deaths increased in women in 2015-22, but a slight increase was found in men after April 2018

**HOW THIS STUDY MIGHT AFFECT RESEARCH, PRACTICE, OR POLICY**

- With improved access to electronic health data with coverage of the whole population, the effect of policy changes and their consequences can be more reliably tracked
- This approach evaluated the use of sodium valproate in the population, which can be used to inform future regulatory changes

## Introduction

 Sodium valproate is an evidence based, antiseizure drug treatment for newly diagnosed idiopathic generalised epilepsy.^1 2^ Sodium valproate is also used in other forms of epilepsy and bipolar affective disorder, as well as in migraine and other conditions. The teratogenic effects of the drug are well known, and when used during pregnancy, sodium valproate can be associated with a range of adverse effects, including increased risk of spontaneous abortion, birth defects, and neurodevelopmental disorders (autism, autism spectrum disorder, attention deficit or hyperactivity disorder, and reduced IQ).^3^,^8^ Major physical malformations are seen in about 10% of babies, with evidence of a dose-response effect.^8^ Guidelines caution against the use of sodium valproate in women of childbearing potential, although they emphasise balancing this risk with effective treatment of life threatening epilepsy.^9^

In 2013, the Medicines and Healthcare products Regulatory Agency (MHRA) strengthened its advice, stating that sodium valproate was "not for use in pregnancy unless there is no effective alternative" and again in 2018, stating that "sodium valproate must no longer be used in any woman or girl able to have children unless she has a pregnancy prevention programme in place".^9 10^ MHRA highlighted that other effective medicines are available for epilepsy and bipolar affective disorder. The Cumberlege report, First do no harm, published in July 2020^11^ during the covid-19 pandemic, reinforced the advice on the use of sodium valproate in women of childbearing potential, and presented sodium valproate as an example of how the health system fails to respond when patients and their families raise concerns about the safety of treatments. In December 2022, the Commission on Human Medicines advised the MHRA to extend reducing the use of sodium valproate in men, based on a full safety review of reproductive toxicity in men, including preclinical and clinical data.^12^,^14^ The guidance now states that sodium valproate should not be started in new patients (men or women), aged <55 years, unless two specialists independently consider and report that no effective alternative treatments are available or that there are reasons that the reproductive risks do not apply. A second specialist signature is also needed for continuation of sodium valproate in women and girls of childbearing potential at their next annual specialist review. This approach is in addition to current safety measures, including the sodium valproate pregnancy prevention plan, which remains in place for any girls and women of childbearing potential.^12^

The direct and indirect effects of the covid-19 pandemic on routine care and actioning of clinical pathways have been well documented, for which a catch-up is required. What has not been shown is the effect, if any, on ongoing regulatory advice, and if implementation of this advice was also slowed during the pandemic. In this study, our aim was to describe the use of sodium valproate in the population of England and Wales during the study period, 1 January 2019 to 31 December 2023, medicines data linked to healthcare data. Based on these data, we investigated if covid-19 affected the ongoing use of sodium valproate by women of childbearing potential, considering the publication of the Cumberlege report and the government's response to it, and regulatory changes.

Our aims were therefore: to describe the use of sodium valproate in the population of England and Wales in 2019-23, after MHRA policy changes (April 2018), the covid-19 pandemic (March 2020), publication of the Cumberlege report (July 2020), and the government's response to it (2021); to compare the use of sodium valproate with other antiseizure drug treatments (lamotrigine, levetiracetam, carbamazepine, and topiramate), and in women versus men; to describe geographical variation in the rate of use of sodium valproate by women of childbearing potential (aged 15-49 years) by local authority district; to describe the use of sodium valproate in women of childbearing potential, including disease indications associated with use; and during pregnancy, including dose and timing of use; and the broader effect of policy changes, with mortality related to epilepsy as an example.

## Materials and methods

### Data sources in England and Wales

We studied de-identified individual level, population scale data accessed through NHS England's Secure Data Environment service for England, from the British Heart Foundation Data Science Centre's CVD-COVID-UK/COVID-IMPACT Consortium, and the Secure Anonymised Information Linkage Databank (SAIL) for Wales.^15 16^ Online supplementary methods has details of the data sources.

In the NHS England Secure Data Environment service, dispensing data from the NHS Business Service Authority are updated on a monthly basis and include prescriptions for all drug treatments dispensed in the community in England.^17^ Dates in NHS Business Service Authority reflect the month when the prescription was submitted for payment rather than the date a drug treatment was dispensed to the patient. The first available month of NHS Business Service Authority data in the English Secure Data Environment was April 2018. We obtained data on indications, concurrent pregnancy, and counselling from the General Practice Extraction Service Data for Pandemic Planning and Research (GDPPR), comprising data from 98% of all English general practices.^15^ Data on indications were also obtained from Hospital Event Statistics.

For Wales, linked population scale, patient level data were accessed through the trusted research environment known as Secure Anonymised Information Linkage Databank.^16^ Dispensing data are from all community pharmacies in Wales available within the Welsh Dispensing Data Set,^18^ which is updated monthly and has records from January 2016 onwards. In the Secure Anonymised Information Linkage Databank on each monthly release of the Welsh Dispensing Data Set, a research ready data asset is created and maintained,^19^ based on covid-19 population e-cohort research ready data asset,^16^ which enhances the dispensing data for research purposes with mapping to additional coding classifications and meta data. Welsh data are presented separately (eg, disease indications, because of non-comparability of mapped codes) or excluded from some analyses (eg, pregnancy, because of insufficient sample size), or both.

### Analyses

#### Prevalent and incident use of sodium valproate and other antiseizure drug treatments

We analysed trends in prevalent (current) and incident (new) uses of sodium valproate before and during the covid-19 pandemic (1 January 2019 to 31 December 2023 in England and 1 January 2019 to 30 June 2022 in Wales). We compared these trends with the dispensing of other commonly used antiseizure drug treatments, including lamotrigine, levetiracetam and brivaracetam, carbamazepine and carbamazepine-like compounds (oxcarbazepine and eslicarbamazepine), and topiramate (online supplemental appendix A). We defined women with childbearing potential as age groups 15-19, 20-29, 30-39, and 40-49 years, and included men of equivalent ages for comparison. Data for sex were taken from information in the data sources used rather than from patient reported gender.

We identified incident (newly dispensed) users of sodium valproate, and also assessed if sodium valproate was the first of any antiseizure drug treatment used, from those included in these analyses. To calculate person level incident drug treatment, we identified the first recorded dispense of sodium valproate in during the study period. We allowed an initial clearance window for the first nine months of data availability to allow monthly incidence counts to stabilise from the high levels of artefact incidence associated with records first becoming available for analysis. April 2018 (the start of NHS Business Service Authority data in the English Secure Data Environment) was used in both the English and Welsh data.

Through linkage of dispensing data of medicines to individual characteristics derived from primary and secondary care datasets,^20^ we investigated how the dispensing of antiseizure drug treatments varied by age and sex. We recorded the number of items of sodium valproate and other antiseizure drug treatments dispensed to men and women by month in England and Wales during the period 1 January 2019 to 31 December 2023 (or 30 June 2022 in Wales). We calculated annual age specific rates of sodium valproate use per 100 000 women of childbearing potential by referencing the relevant mid-year populations for England, available from the Office for National Statistics,^21^ and compared these rates with rates in men of equivalent ages.

Among women of childbearing potential who were dispensed sodium valproate, we investigated the most common combinations of other antiseizure drug treatments dispensed in this population for the most recent calendar year of the study period. We calculated the percentage of women dispensed more than one antiseizure drug treatment as a potential indication of the severity or control of epilepsy, or both, as well as reporting the percentage of individuals only taking sodium valproate.

#### Geographical variation in use of sodium valproate

Based on geographical information at the point of dispense (lower layer super output area mapped to local authority district), we analysed variation in the rates of use of sodium valproate by women of childbearing potential in England.

#### Indications for sodium valproate

In women of childbearing potential, we investigated the main disease indications (epilepsy, bipolar affective disorder, both, or neither) associated with dispensing sodium valproate based on Health Data Research UK phenotypes^22^ (online supplemental appendix B; codes clinically reviewed for relevance by CT and TW). In Wales, validated pre-existing definitions of the study diseases were used.^23^ Records from GDPPR and Hospital Event Statistics were linked to the dispensed drug treatment records with the pseudo-identifier identification number (a non-identifying unique master key that replaces the NHS number across all datasets) and screened for disease codes recorded in the electronic health record at any point up to the time when sodium valproate was dispensed. Results for Wales are presented separately because mapping codes exactly across countries was not possible. The prevalence of disease indication estimates at the time the drugs were dispensed are presented by age group, per 1000 women dispensed sodium valproate in each calendar year. We also evaluated data recorded in the general practice electronic health record (GDPPR) of implementation of the pregnancy prevention programme in women of childbearing potential by calendar year (online supplemental appendix C has details of the codes), and assessed whether this information differed by disease indication.

#### Pregnancy rates in women of childbearing potential dispensed sodium valproate and other antiseizure drug treatments

We investigated when an antiseizure drug treatment was dispensed within an estimated pregnancy episode. A pregnancy episode was defined through linkage of maternity records in Hospital Event Statistics, where possible, or otherwise by pregnancy code recorded in GDPPR (online supplemental appendix D). From the maternity records in Hospital Event Statistics, the pregnancy period could be estimated based on a known date of delivery, miscarriage, or abortion. Online supplementary methods has further details on identification of the pregnancy episode. Based on these pregnancy episodes, we identified annual trends in the age specific rates of pregnancy per 1000 women of childbearing potential who were dispensed an antiseizure drug treatment during the period 2019-22, grouped by antiseizure drug treatment.

We recorded reports of drugs dispensed during the first trimester (days 1-90 of the pregnancy episode) and calculated the average quantity (in g/day) of sodium valproate dispensed during the whole 40 weeks of pregnancy and by trimester. In the NHS Business Service Authority data, each dispensing event has information about the quantity (eg, number of items, tablets) and medicine strength (concentration in mg or mg/mL). Dose was calculated by multiplying the quantity of items at each event by the concentration after converting to grams. The total number of grams dispensed was summed over the total pregnancy episode and during each trimester. This value was divided by the corresponding number of days to calculate average g/day (280 for the whole pregnancy episode; 90 for the first trimester).

#### Trends in mortality related to epilepsy and recurrent seizures by sex

We examined English mortality data from the Office for National Statistics, available in the Secure Data Environment. We extracted deaths by month, from January 2015 to December 2022, for ICD-10 (international classification of diseases, 10th revision) codes G40 (epilepsy and recurrent seizures) and G41 (status epilepticus) where these codes appeared anywhere on the death certificate. Grouped by sex, we fitted linear regression models to these data for the periods before and after advice from MHRA in March 2018, recording the slope and 95% confidence intervals from each model. Also, we used time series analysis with autoregressive integrated moving average models fitted to the earlier period, January 2015 to March 2018, to predict the expected trend in deaths related to epilepsy from April 2018 onwards. This analysis was undertaken with the auto.arima function from the forecast package in R (version 4.2.2). Stationarity was tested with the augmented Dickey-Fuller and Kwiatkowski-Phillips-Schmidt-Shin tests. This analysis was performed according to a prespecified analysis plan published on GitHub, along with the phenotyping and analysis code (https://github.com/BHFDSC/CCU014_03).

### Patient and public involvement

Our protocol and manuscript were reviewed by patient and public participants from the British Heart Foundation Data Science Centre. Patient participants were engaged throughout the research process, providing valuable comment and feedback following presentation of protocols and preliminary results.

## Results

**Figure 1:**
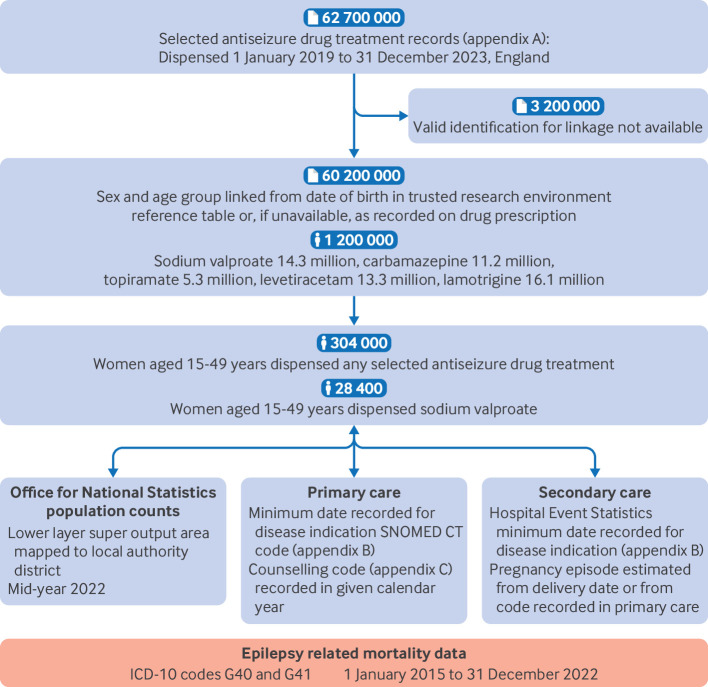
Flowchart illustrating the data sources and linkage between datasets included in the analysis for England. ICD-10=international classification of diseases, 10th revision, codes G40 (epilepsy and recurrent seizures) and G41 (status epilepticus). SNOMED CT=Systematised Nomenclature of Medicine Clinical Terms

Figure 1 shows the data sources used in the study and linkage between datasets for England, and online supplemental figure 1 shows the data sources for Wales.

### Prevalent and incident use of sodium valproate and other antiseizure drug treatments

**Figure 2:**
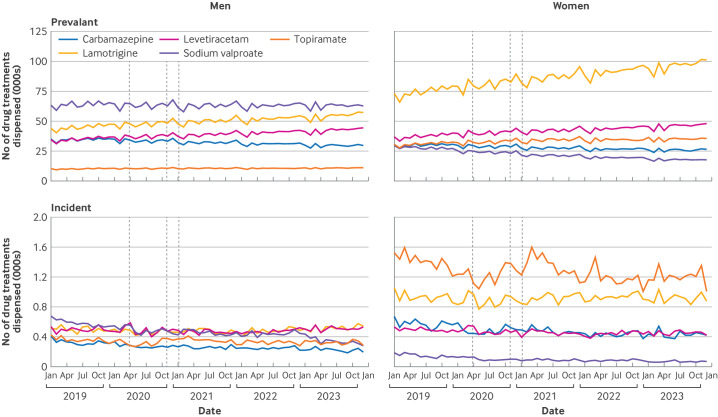
Monthly numbers of prevalent and incident antiseizure drug treatments dispensed in the community from January 2019 to December 2023 in men and women aged 15-49 years, in England. Vertical lines indicate dates of pandemic lockdown periods for reference

The monthly number of dispensings of sodium valproate to women aged 15-49 decreased before, during, and after the pandemic, whereas in men the numbers were constant across the study period in England and Wales (figure 2 and online supplemental figure 2). Prevalent rates of sodium valproate dispensed to women of childbearing potential decreased in all age groups in England between 2019 and 2022, contrasting with rates in men of similar age which were more stable and at a higher level (table 1). Monthly numbers of other antiseizure drug treatments in women of childbearing potential, such as lamotrigine, levetiracetam, and topiramate, increased over the study period (figure 2). Prevalent rates of sodium valproate use were about four times higher among women aged 40-49 than in women aged 20-29 (table 1).

**Table 1:** Incident and prevalent rates of sodium valproate dispensed, in women and men aged 15-49 years, in England 2019-22, by age group

	Women				Men			
	2019	2020	2021	2022	2019	2020	2021	2022
**Rates of sodium valproate dispensed/100 000, by age group (years)**								
Prevalent use:								
15-19	729	636	602	595	2787	2934	2801	2871
20-29	1110	1069	968	892	4426	4646	4720	4634
30-39	2172	1915	1714	1549	6029	6222	6302	6307
40-49	5013	4590	4100	3709	8151	8361	8364	8313
Incident use:								
15-19	7	5	5	5	53	46	40	43
20-29	11	7	6	7	59	52	50	47
30-39	14	10	9	7	57	48	44	42
40-49	23	15	12	12	53	42	39	35
**Total No of of sodium valproate prescriptions dispensed by age group (years)**								
Prevalent use:								
15-19	11 660	10 330	9975	10 090	44 570	47 630	46 390	48 720
20-29	40 045	37 950	34 105	31 915	159 590	164 930	166 215	165 725
30-39	81 260	71 625	64 285	58 855	225 590	232 700	236 410	239 635
40-49	179 005	163 040	144 360	130 270	291 055	296 965	294 515	291 970
Incident use:								
15-19	105	85	80	90	845	745	665	735
20-29	380	245	230	235	2130	1835	1755	1670
30-39	505	385	325	260	2140	1805	1665	1610
40-49	805	525	435	420	1885	1500	1370	1230

Incident rates of sodium valproate dispensed in women of childbearing potential in England decreased between 2019 and 2022 (figure 2 and table 1), from seven to five in women aged 15-19, from 11 to seven in women aged 20-29 years, from 14 to seven in women aged 30-39 years, and from 23 to 12 in women aged 40-49 years (rates per 100 000 women). Incident use also decreased in men of the same ages in the same time period, from 53 to 43 in men aged 15-19 years, from 59 to 47 in men aged 20-29 years, from 57 to 42 in men aged 30-39 years, and from 53 to 35 in men aged 40-49 years (rates per 100 000 men) but rates were much higher than in women (figure 2 and table 1). In 2023, fewer than 400 women in England aged 15-49 years were dispensed sodium valproate without first receiving another antiseizure drug treatment from those selected for inclusion in this analysis (online supplemental figure 3).

Distribution of the number of sodium valproate prescriptions dispensed differed markedly by sex, and compared with other antiseizure drug treatments, were much lower in women of childbearing potential than in men in the same age groups (online supplemental figure 3). In contrast, uptake of the alternative antiseizure drug treatments, particularly lamotrigine and topiramate, was higher in women of childbearing potential than in men of the same age (online supplemental figure 3). At age >60 years, sodium valproate use was more balanced between men and women. In 2023, 64% of all women of childbearing potential dispensed sodium valproate were using this drug treatment exclusively in that calendar year, whereas 30% were dispensed two antiseizure drug treatments. Levetiracetam and lamotrigine were the most commonly dispensed drugs in combination with sodium valproate (online supplemental figure 4).

### Geographical variation in use of sodium valproate

**Figure 3:**
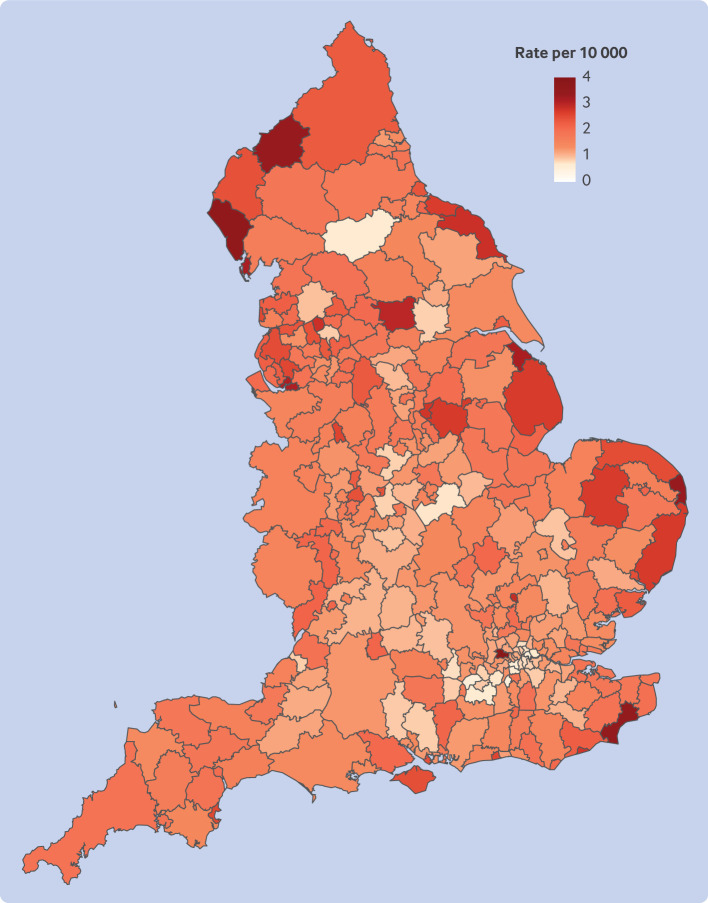
Geographical distribution of sodium valproate dispensed to women of childbearing potential in England by local authority district, in women aged 15-49 years, in 2022

We saw geographical differences in the rates of sodium valproate dispensed to women of childbearing potential. Local authority districts in the northwest, eastern coastal, and bordering Scotland had some of the highest rates (figure 3 and online supplemental figure 5).

### Indications for sodium valproate

**Figure 4:**
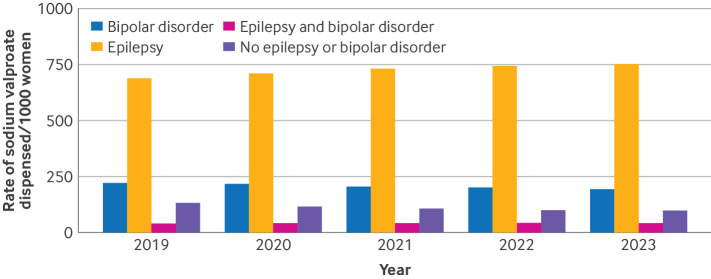
Rates of sodium valproate dispensed in women aged 15-49 years with evidence of epilepsy or bipolar affective disorder in the electronic health record, 2019-23 in England

We found that in women of childbearing potential dispensed sodium valproate, the most common indication was epilepsy (~751 per 1000 women in 2023) and bipolar affective disorder (~193 per 1000 women), with this trend becoming more pronounced over time (figure 4). In about 10% of women of childbearing potential, however, sodium valproate was dispensed without evidence of either of these indications available in the electronic health record (figure 4 and online supplemental figure 6). Indication patterns were similar in Wales, but with an even higher proportion without evidence of epilepsy or bipolar affective disorder, possibly reflecting incomplete mapping of code lists (online supplemental figure 6). Bipolar affective disorder was a more common indication in older groups of women of childbearing potential (30-39 and 40-49 years), with the younger groups (15-19 and 20-29 years) dominated by the epilepsy indication (online supplemental figure 7).

### Pregnancy rates in women of childbearing potential dispensed sodium valproate and other antiseizure drug treatments

**Figure 5:**
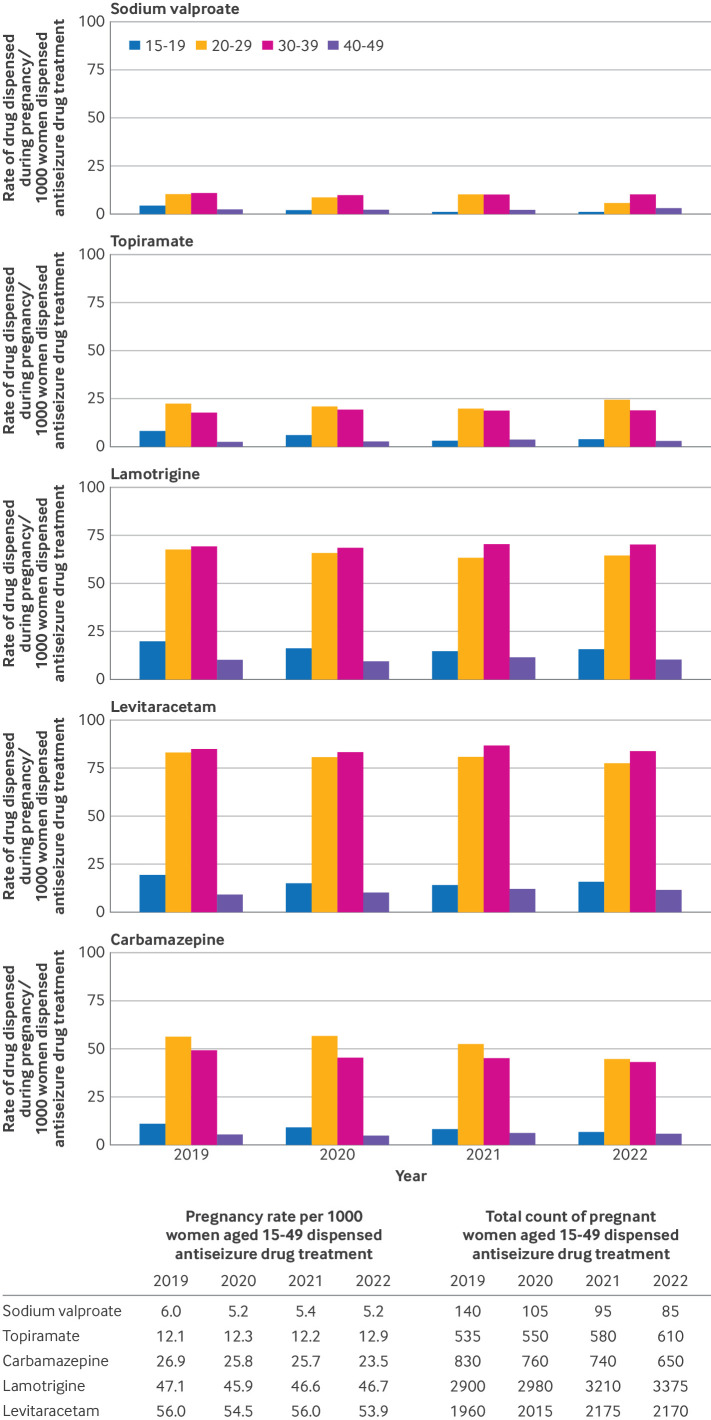
Rates of dispense during a pregnancy episode per 1000 women dispensed antiseizure drug treatment in women aged 15-49 years, 2019-22 in England, by age group and antiseizure drug treatment

The number of women of childbearing potential with any evidence of sodium valproate dispensed during pregnancy decreased from 6.0 in 2019 to 5.2 per 1000 women in 2022 in England (figure 5). These rates were lower than for other antiseizure drug treatments, with the highest rates reported for levetiracetam and lamotrigine. We also saw lower pregnancy rates for women of childbearing potential dispensed topiramate (12.1 women per 1000 in 2019), although rates increased slightly in 2022 to 12.9. Absolute numbers of women dispensed sodium valproate during a pregnancy episode decreased from 140 in 2019 to 85 in 2022 (figure 5). Assigning each pregnancy to the year in which the pregnancy episode commenced to allow for pregnancies that spanned two calendar years resulted in lower numbers (112 in 2019 and 62 in 2022, respectively) (table 2). Most women dispensed sodium valproate during the pregnancy episode received some during the first trimester. Most women received an average dose of <1 g/day of sodium valproate over the course of the pregnancy episode. Only 11 women were dispensed an average dose of >1 g/day during pregnancies commenced in 2022; 15 received >1 g/day in the first trimester.

**Table 2:** Use of sodium valproate in women during pregnancy in England, 2019-22. Each pregnancy was assigned only to the year in which it commenced (n=325)

Use of sodium valproate	Year of start of pregnancy			
	2019	2020	2021	2022
Total No of pregnant women	112	73	78	62
No of women dispensed sodium valproate in first trimester	102	69	72	57
Average dose dispensed during pregnancy (g/day):				
<0.5	73	48	41	34
0.5-1	23	14	18	17
>1	16	11	19	11
Average dose dispensed in first trimester (g/day):				
<0.5	48	28	33	17
0.5-1	26	26	19	25
>1	27	14	22	15
Average dose dispensed in second and third trimesters (g/day):				
<0.5	38	17	19	17
0.5-1	17	14	11	19
>1	16	10	25	<10

Although some evidence of counselling according to the pregnancy prevention plan was recorded in GDPPR, we found no evidence of counselling with the available codes for most women who received sodium valproate. This pattern was consistent across disease indications, although we saw some evidence for increased recording of these codes over time (online supplemental figure 8).

### Trends in mortality related to epilepsy by sex

**Figure 6:**
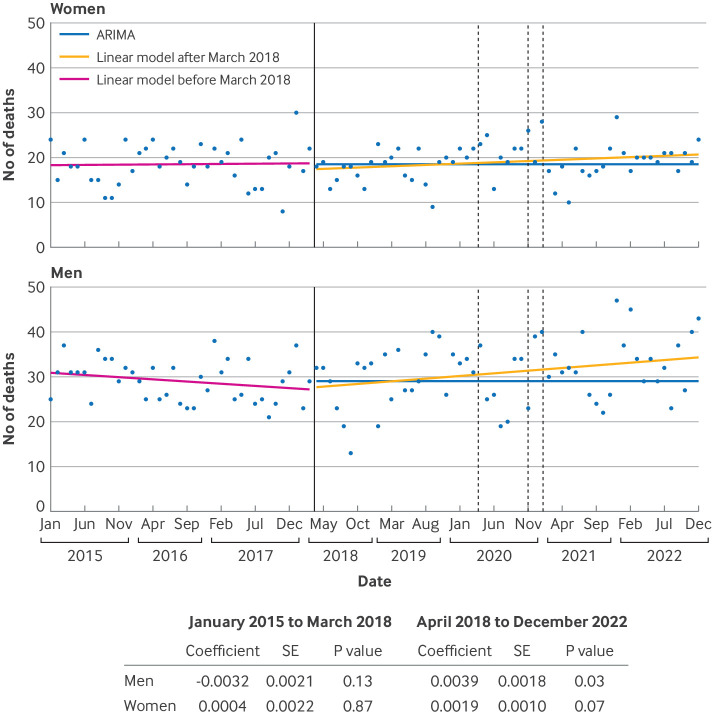
Mortality related to epilepsy and recurrent seizures over time based on ICD-10 (international classification of diseases, 10th revision) codes G40 (epilepsy and recurrent seizures) and G41 (status epilepticus). Number of deaths in men and women of childbearing age, 15-49 years, by year and month, 2015-22 in England. Solid vertical line indicates Medicines and Healthcare products Regulatory Agency (MHRA) policy change; broken vertical line indicates pandemic national lockdown periods. Linear models before and after March 2018 refer to before and after policy recommendations made by MHRA in March 2018. ARIMA=autoregressive integrated moving average model; SE=standard error

Linear regression of the number of deaths related to epilepsy for the period January 2015 to March 2018 showed no evidence of a change in death over time between men and women of childbearing age (figure 6). The autoregressive integrated moving average model fitted to the period January 2015 to March 2018 predicted constant death counts for the period after March 2018: 28 (95% confidence interval 26.7 to 29.3) deaths related to epilepsy each month for men and 18 (16.2 to 19.0) for women. Beta coefficients from the linear models were positive during the subsequent period from April 2018 to December 2022 for both sexes, with stronger evidence for change in men (β=0.0039, P=0.034) than in women (β=0.0019, P=0.066).

## Discussion

### Principal findings

In this study, we used data that linked dispensed drug treatments to disease indications and personal characteristics for the whole population of England and Wales to comprehensively describe the epidemiology of the use of sodium valproate. This design overcomes the limitations of sampling in the epidemiological studies carried out so far. This approach can also provide a mechanism for regulators and those responsible for delivery of healthcare to track changes in the dispensing of medicines and how this behaviour varies by characteristics, including age, sex, geography, indication, pregnancy, and other comorbidities in near real time. Based on these data, we quantified the rates of use of sodium valproate, comparing use in women and men, and with other antiseizure drug treatments during the study period. The study period included the covid-19 pandemic, the Cumberlege report, and followed policy recommendations by MHRA. We investigated the indications for sodium valproate and described geographical variation in the use of sodium valproate. We also defined a systematic approach to dispensing of sodium valproate to women of childbearing potential, including during pregnancy, and the dose dispensed during different developmental periods of pregnancy.

Our findings showed that prevalent use of sodium valproate during the period 2019-23 decreased in women of childbearing potential, although no changes were seen in men of equivalent age. Geographical differences remained, however, in the rate of sodium valproate dispensed to women of childbearing potential by local authority district. For incident use, decreases were seen in both women of childbearing potential and men. Pregnancy rates in those taking sodium valproate decreased from 6.0 to 5.2 pregnancies per 1000 women aged 15-49 years in England. These findings expand previous work and show that whole population linked records are useful to track and implement the safety of medicines.^24^,^28^ Overall, these data showed evidence of harm reduction (meaningful reduction in the use of sodium valproate in pregnancies that had a high risk of serious lifelong harms) without an increase in the most serious epilepsy related harm of deaths in women during the study period.

### Strengths and limitations of this study

Our data science approach was helpful for many aspects of this study, but more research is needed, taking into account the limitations of our analyses, which could be supplemented by further investigation. Firstly, off-label use of sodium valproate could be a useful first target. Sodium valproate is the antiepileptic drug of first choice for newly diagnosed idiopathic generalised epilepsy, based on evidence from the Standard and New Antiepileptic Drugs (SANAD) trials.^1 2^ Sodium valproate is, however, also used in other forms of epilepsy and in bipolar affective disorder, however, where the evidence base is poor and other drugs are similarly effective. Codes for subtypes of epilepsy were not available, and therefore identifying the use of sodium valproate for non-idiopathic generalised epilepsy was not possible. In 10% of women dispensed sodium valproate, however, we could not identify a previous indication for epilepsy or bipolar affective disorder in the electronic health record, suggesting potential off-label use. Incomplete recording or availability of codes mean that routine data lack the sensitivity to capture all episodes of epilepsy or bipolar affective disorder.^29^ Codes for migraine were not available in GDPPR.

Secondly, our results showed that the number of pregnant women who used sodium valproate decreased during the study period. This finding could be attributed to implementation of MHRA guidance, although other factors, such as reduced contact with the healthcare system and lower rates of birth, were also a characteristic of the pandemic that might have contributed to these trends and hence ongoing pharmacovigilance is essential. Our approach provides a comprehensive method for pharmacovigilance, but also shows that with linked data, individual drug registries might not be needed, but rather that pipelines within the linked data can be informative for a range of drugs. Pipelines refer to algorithms that could be automatically applied in a systematic way across all drug treatments within healthcare data to present these types of results in real time. The potential ofthis approach was highlighted in our study with other antiseizure drug treatments. For example, concern has been raised about the teratogenic effect of topiramate from the Nordic Registry,^28^ although a US study did not find a higher risk of autism after the use of topiramate.^30^ Topiramate was classed as an antiseizure drug treatment for our research, according to the British National Formulary, but topiramate is also used for other conditions, such as migraine. Our data showed that prevalent use of topiramate increased over our study period. We also found that pregnancy rates were higher in women taking topiramate and might be increasing. The data science approach described here could be replicated for topiramate, without having to establish a registry, but rather by reusing codes across medicines and other relevant characteristics.

Thirdly, like any chronic condition, a switch away from a drug that has provided good control can contribute to loss of control. This effect is true of epilepsy, and loss of control might increase the number of seizures and, as a result, increase the use of healthcare facilities, and in extreme cases cause death, including sudden unexpected death in epilepsy. Epilepsy, particularly when seizures are not controlled, is associated with an increased risk of psychiatric disorders, cognitive impairment, cardiovascular and bone disease, physical injuries, and increased mortality.^31^ That sodium valproate is still used during pregnancy might be explained by the wish to avoid such complications, including sudden unexpected death in epilepsy (SUDEP). During the review of this manuscript, the Patient Safety Commissioner published the Hughes report, highlighting the importance of shared decision making between women of childbearing potential, pregnant women, and those treating their epilepsy.^32^ This practice needs to be in place despite pressures on the system, given the lasting effects of harms to the mother or fetus, which have been economically quantified.^33^ Our approach aims to enhance pharmacovigilance by incorporating its use into the healthcare system, but also by encouraging further research. For example, we have quantified the dose of sodium valproate used during pregnancy as <1 g/day for most individuals. This finding could be useful to more precisely understand dose-response relations, facilitated by linked maternal and child records. This research could also put UK data in the context of international findings, allowing us to more fully understand benefit and risk profiles.^34^

Linked to this research is the importance of tracking any unintended effects when switching away from sodium valproate. The confidential enquiry investigating maternal deaths (Mothers and Babies: Reducing Risk through Audit and Confidential Enquiries, MBRRACE-UK^35^) is an important source of information that aims to improve maternal healthcare and wellbeing. The enquiry found that sudden unexpected death in epilepsy could be increasing in women who are pregnant and during the postpartum period: eight sudden unexpected deaths in epilepsy were reported in 2013-15, 18 in 2016-18, and 14 in 2019-21. In our analyses, we did not find an increase in deaths related to epilepsy in women of childbearing potential although the codes we used were broad and not limited to sudden unexpected death in epilepsy. Our analyses and the MBRRACE-UK report cannot causally link switching away from sodium valproate with mortality. Our data, together with the approach used by MBRRACE, however, could help in understanding these associations and also extend analyses to morbidity among women (eg, seizure control, hospital admissions, and epilepsy subtypes), as well as sequential or combined use of antiseizure drug treatments and their effects on epilepsy related outcomes.

We saw an emerging mortality trend in men. The timing of this trend did not overlap with recent advice from MHRA for men. More research is needed to determine the reasons for this trend, but this study showed the usefulness of a national approach for regulators and those implementing health policy. This finding is particularly important at a time when further changes to MHRA advice are being implemented, restricting sodium valproate use in men as well as women aged <55 years. Our data science and flexible pipeline approach allows near real time tracking to improve pharmacovigilance strategies, extending the analysis presented here by incorporating other analyses (eg, use of healthcare facilities). Trends over time, however, are required to reliably interpret the effect on outcomes such as death, because variation in the population for other reasons (eg, the covid-19 pandemic in this study) could be responsible for year-to-year variations.

Fourthly, shared decision making was highlighted by the Cumberlege report; many women taking sodium valproate were unaware of the harms associated with the drug. Based on a data science approach, we found no primary care evidence of pregnancy prevention programmes for most women. Implementation of pregnancy prevention programme mostly occurs in secondary care, however, and this information might not always transfer to the primary care record in a timely fashion or at all. Furthermore, this information might be stored as free text rather than recorded codes and was therefore not captured in our study. An audit of specialists suggested that the pregnancy prevention programme is being implemented but is not universal despite the regulatory advice.^36^ Digitisation of the risk acknowledgement form in primary and secondary care might give greater visibility to healthcare professionals and facilitate monitoring of trends in counselling. Greater sharing of this information with primary care to help identify and monitor these individuals is important because MHRA guidance places the onus on general practice to ensure individuals are counselled, despite the fact that counselling is provided in secondary care. Another limitation is that GDPPR does not include all SNOMED CT codes that might be relevant for pregnancy prevention programmes and the annual risk acknowledgement form, and evidence might therefore be underestimated in these analyses.

Finally, the study had some limitations that are important to acknowledge. Future research could include understanding outcomes and health trajectories in those individuals switched away from sodium valproate, based on individual level data, to understand whether seizure control might be linked to the switched medicines, or if we need to investigate other factors that contribute to poor control. In this study, we focused on mortality to examine unintended effects; poor control might also be indicated by increased use of healthcare facilities, which we did not investigate. Some limitations of the data include the lack of dispensing data before April 2018 in NHS England's Secure Data Environment. Access to these data would have facilitated more formal modelling of the effect of the policy recommendations made by MHRA in 2018. At least some of the prescriptions for sodium valproate dispensed without an indication for epilepsy or bipolar affective disorder might be explained by incomplete data in the electronic health record, including partial coverage of codes or incomplete mapping or linkage of medicines data to other datasets, or both.

Other limitations of this analysis include assessment of real life adherence, because what proportion of women who were dispensed sodium valproate actually took the drug during pregnancy was not known. Accurately determining pregnancy episodes is challenging in electronic health record data, and it is not possible to determine if pregnancies were planned or unplanned in this study because of recording and reporting biases. Determining the use of contraception would have been informative but we could not record this information because not all forms of contraception are recorded in the electronic health record. Further research to understand the context of the increasingly uncommon but continued use of sodium valproate to pregnant women is needed. Also, we could not incorporate data from Wales in all analyses (eg, pregnancy rates by antiseizure drug treatment) because of potential identification of individuals, demonstrating the need for large datasets to understand rare conditions or rare treatments.

### Conclusions

In this study, we found continuing but slowly declining use of sodium valproate by women of childbearing potential in England and Wales, including in pregnancy, during the study period, 2019-23, which included the covid-19 pandemic. Incident use of sodium valproate also decreased in men of the same age but remained at much higher levels. Ongoing pharmacovigilance is essential to track the use of this teratogenic medicine and to monitor changes affected by regulatory changes. Analyses could be extended to epilepsy outcomes, including outpatient and emergency attendance, and sudden unexpected death in epilepsy.

## Supplementary material

10.1136/bmjmed-2023-000760 online supplemental file 1

10.1136/bmjmed-2023-000760 online supplemental file 2

## Data Availability

Data may be obtained from a third party and are not publicly available.
