# Supplementary material for: Use of sodium valproate and other antiseizure drug treatments in England and Wales: quantitative analysis of nationwide linked electronic health records
Source: BMJ Med. 2024 Dec 20;3(1):e000760. doi: 10.1136/bmjmed-2023-000760 (PMC12164316; doi:10.1136/bmjmed-2023-000760)
Supplement: online supplemental file 1 [file bmjmed-3-1-s001.pdf]

SUPPLEMENTARY MATERIALS

Supplementary Figure 1: Flowchart to illustrate data sources and linkage between datasets included in analysis, Wales

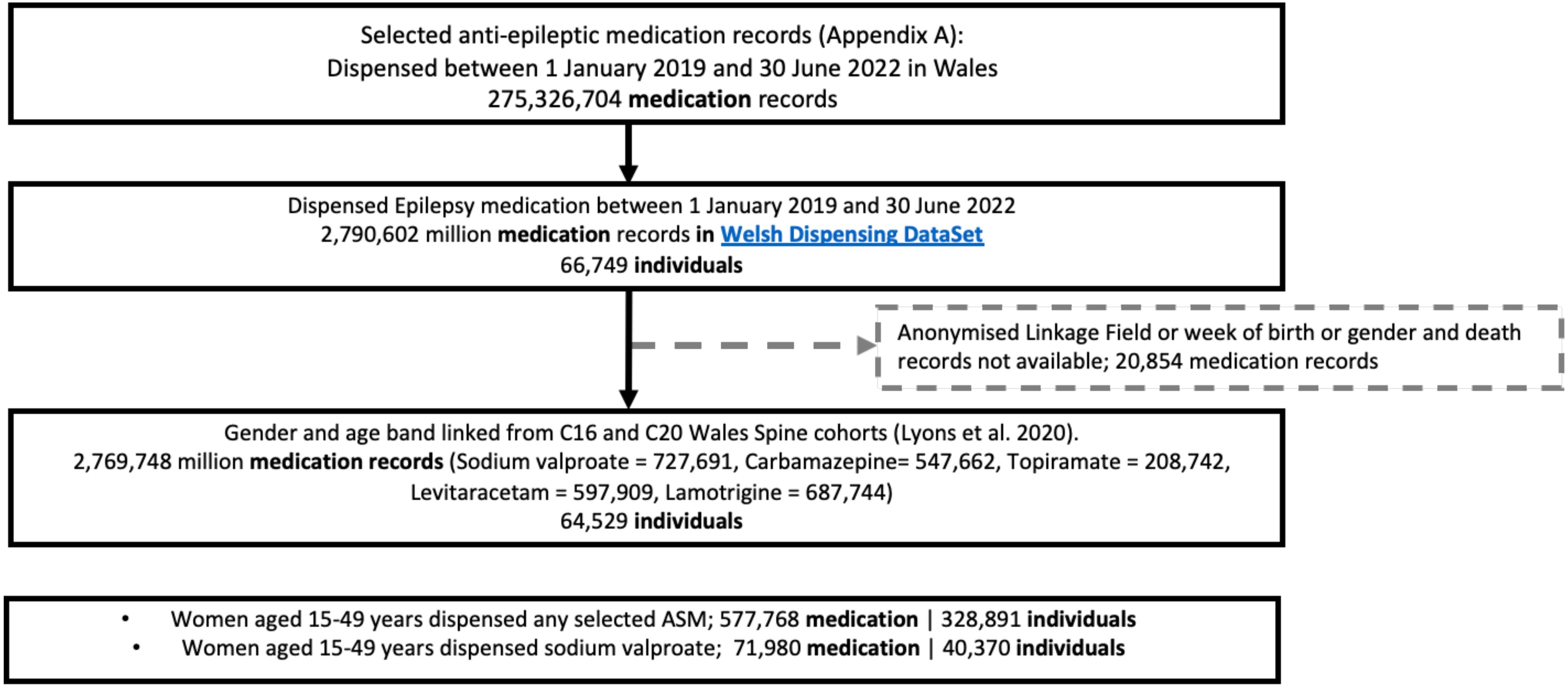

Supplementary Figure 2: Monthly counts of prevalent and incident ASMs dispensed in the community January 2019 to December 2023; men and women aged 15-49 years, Wales. Vertical lines indicate dates of pandemic lockdowns for reference.

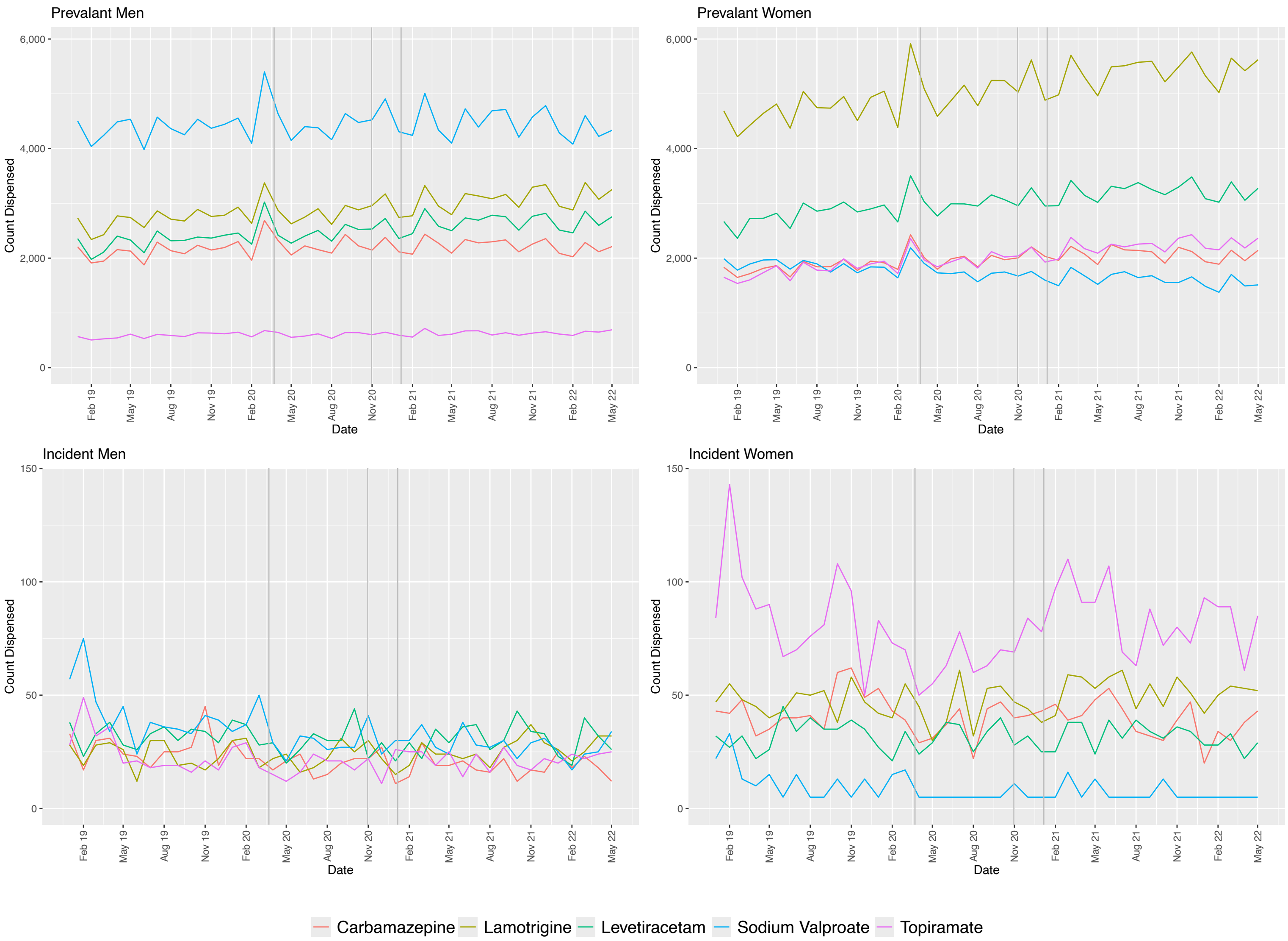

Supplementary Figure 3: Age- and sex- distribution of count of SV dispenses compared with other ASMs (lamotrigine, levetiracetam, topiramate, carbamazepine) in England 2023; prevalent, incident and incident of all selected ASMs

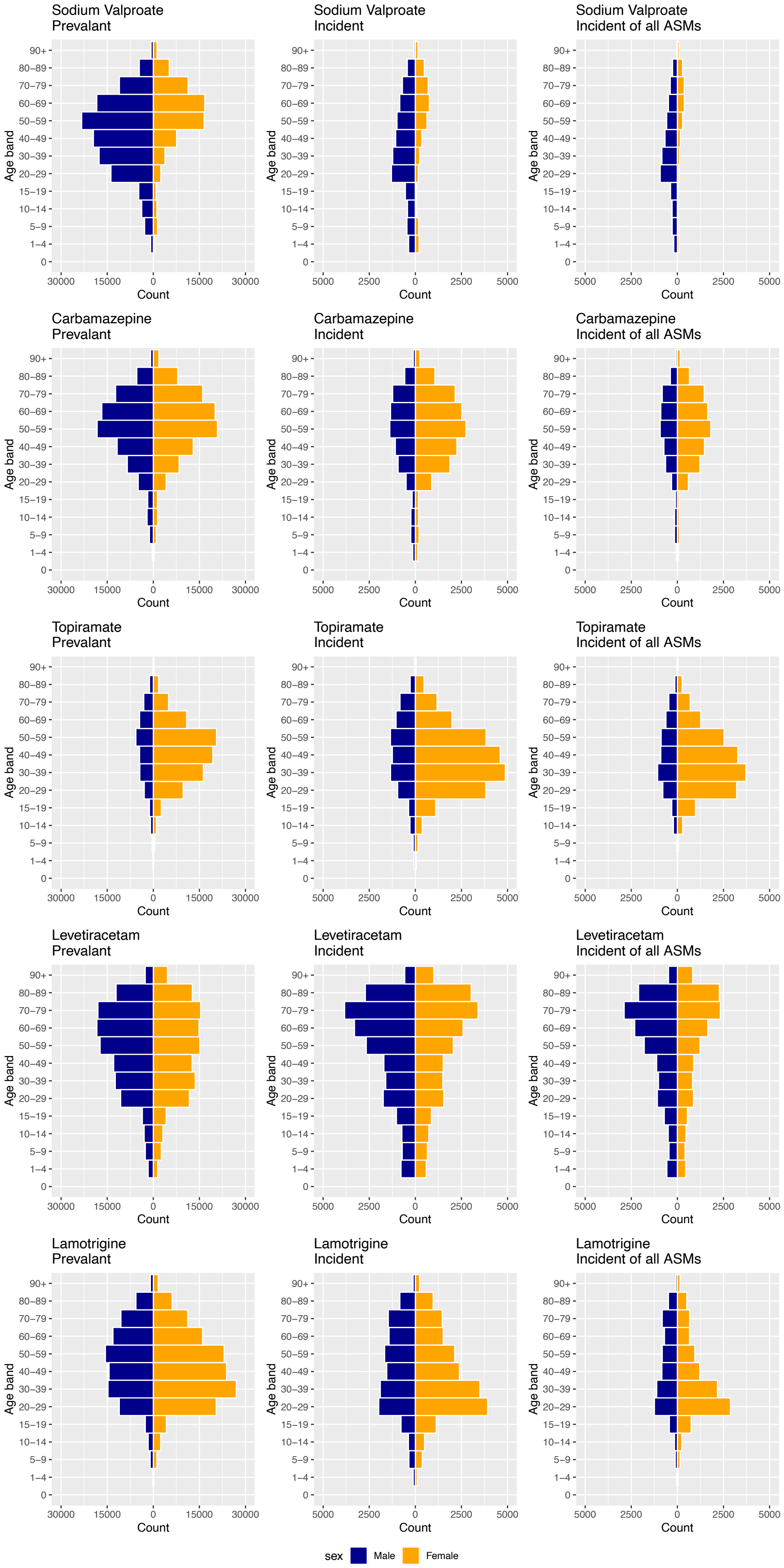

<u>Footnote</u>: 'Prevalent' is all dispenses a given ASM, 'incident' is the first dispense of a given ASM to an individual, 'incident of all ASMs' indicates if the first dispense of the given ASM was also the first dispense of all the selected ASMs to an individual

Supplementary Figure 4: Distribution of selected ASMs taken in combination with sodium valproate; women of CBP dispensed sodium valproate in England 2023

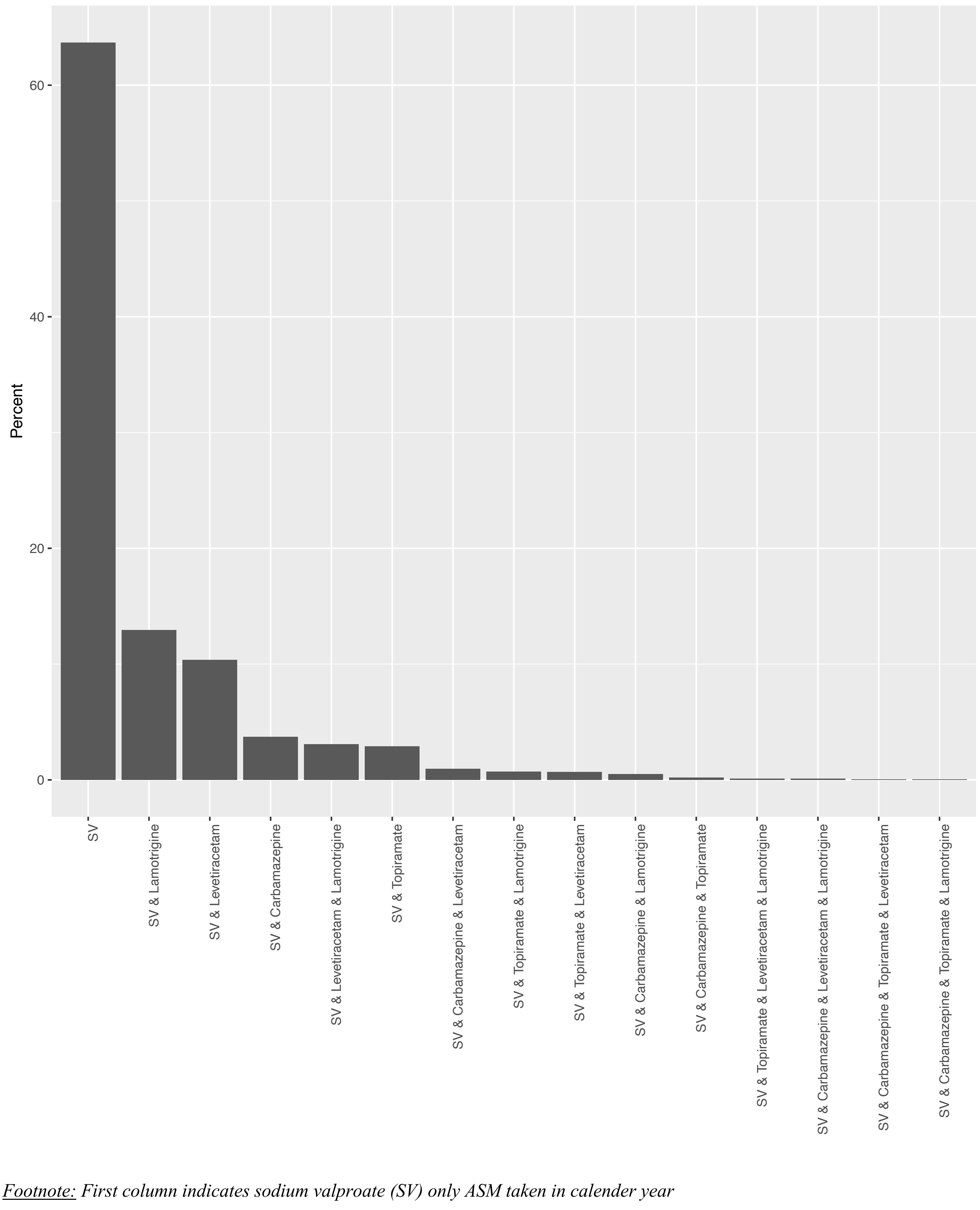

<u>Footnote:</u> *First column indicates sodium valproate (SV) only ASM taken in calender year*

Supplementary Figure 5: Geographical distribution of sodium valproate dispensed to women of CBP in England by Local Authority District, 2022; rate per 10,000 women by age band

2022 , Age Band 15–19

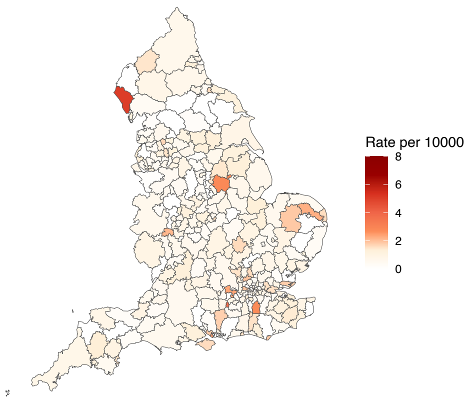

2022 , Age Band 20–29

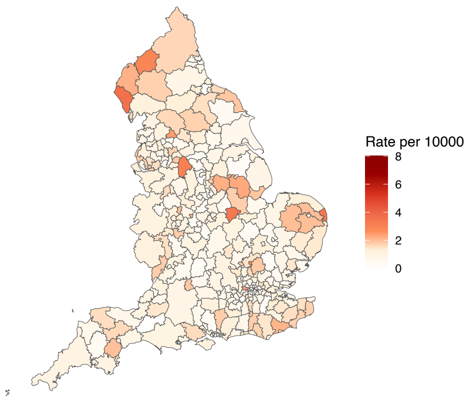

2022 , Age Band 30–39

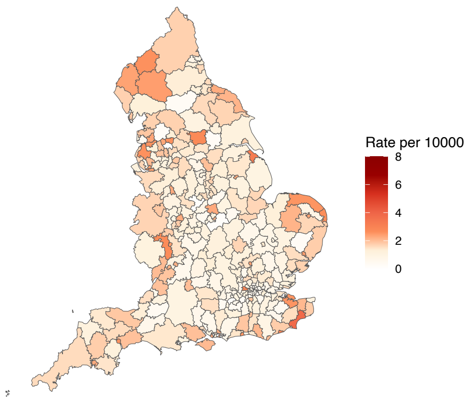

2022 , Age Band 40–49

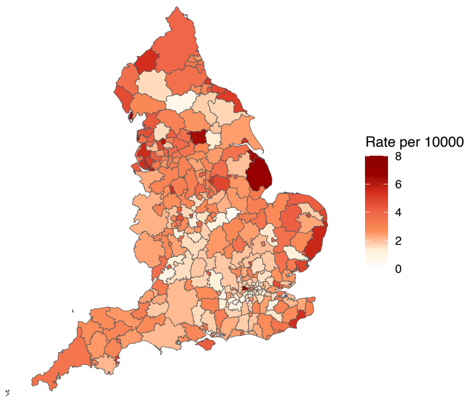

Supplementary Figure 6: Women aged 15-49 years dispensed sodium valproate with evidence of epilepsy or bipolar disorder in the electronic health record by year 2019-2021 in Wales; rate per 1000

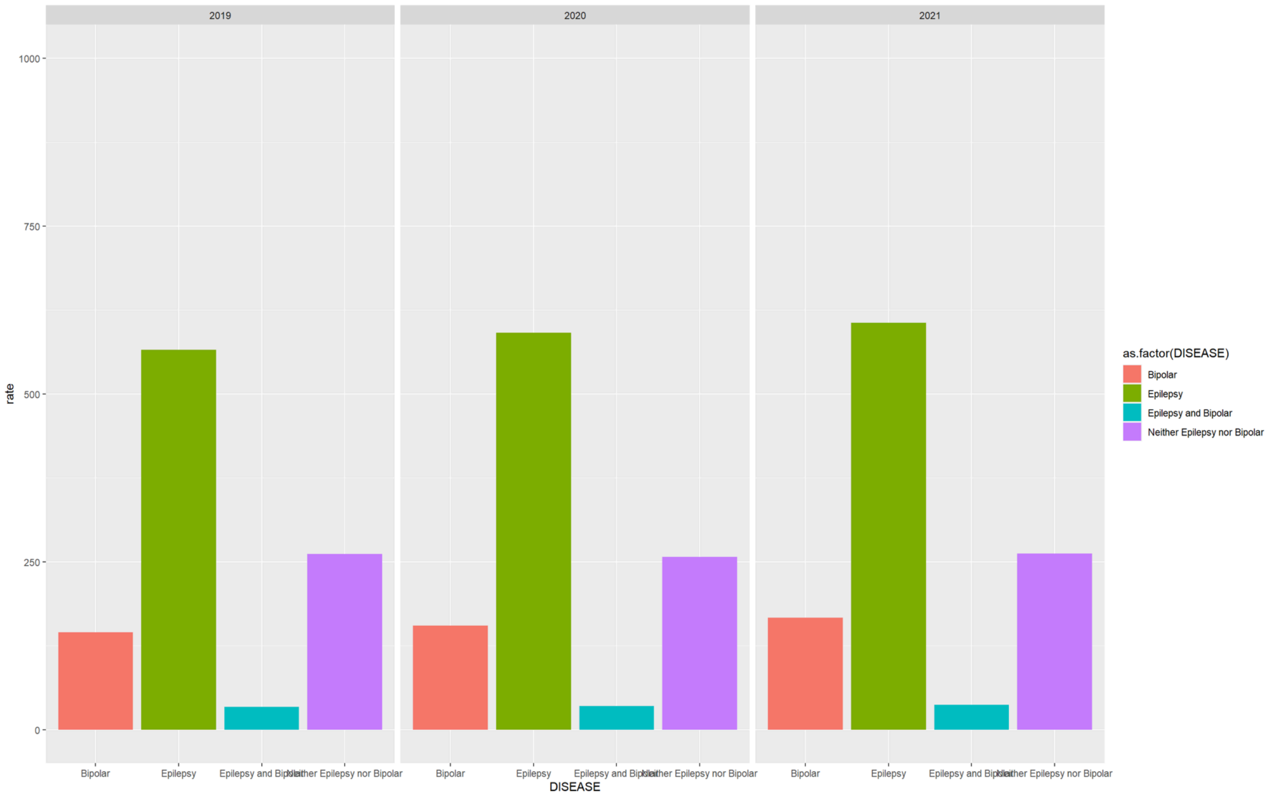

Supplementary Figure 7: Women aged 15-49 years dispensed sodium valproate with evidence of epilepsy or bipolar disorder in the electronic health record by age band and year 2019-2023 in England; rate per 1000

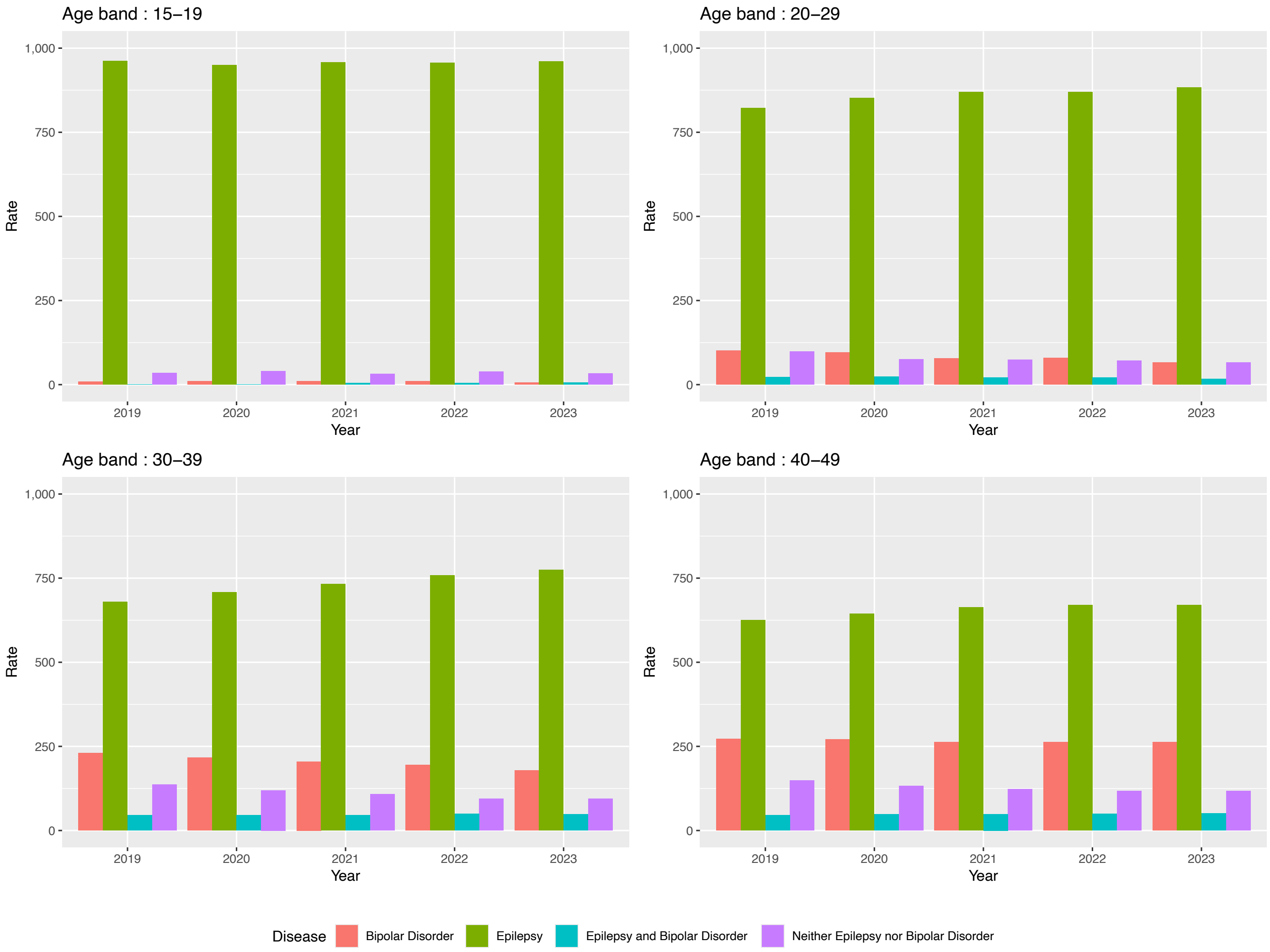

Supplementary Figure 8: Indication (epilepsy, bipolar disorder, both, neither) associated with dispense of sodium valproate overlaid with record of counselling code (contraception and/ or pregnancy) in same calendar year as dispense of sodium valproate in England 2019-2023; women

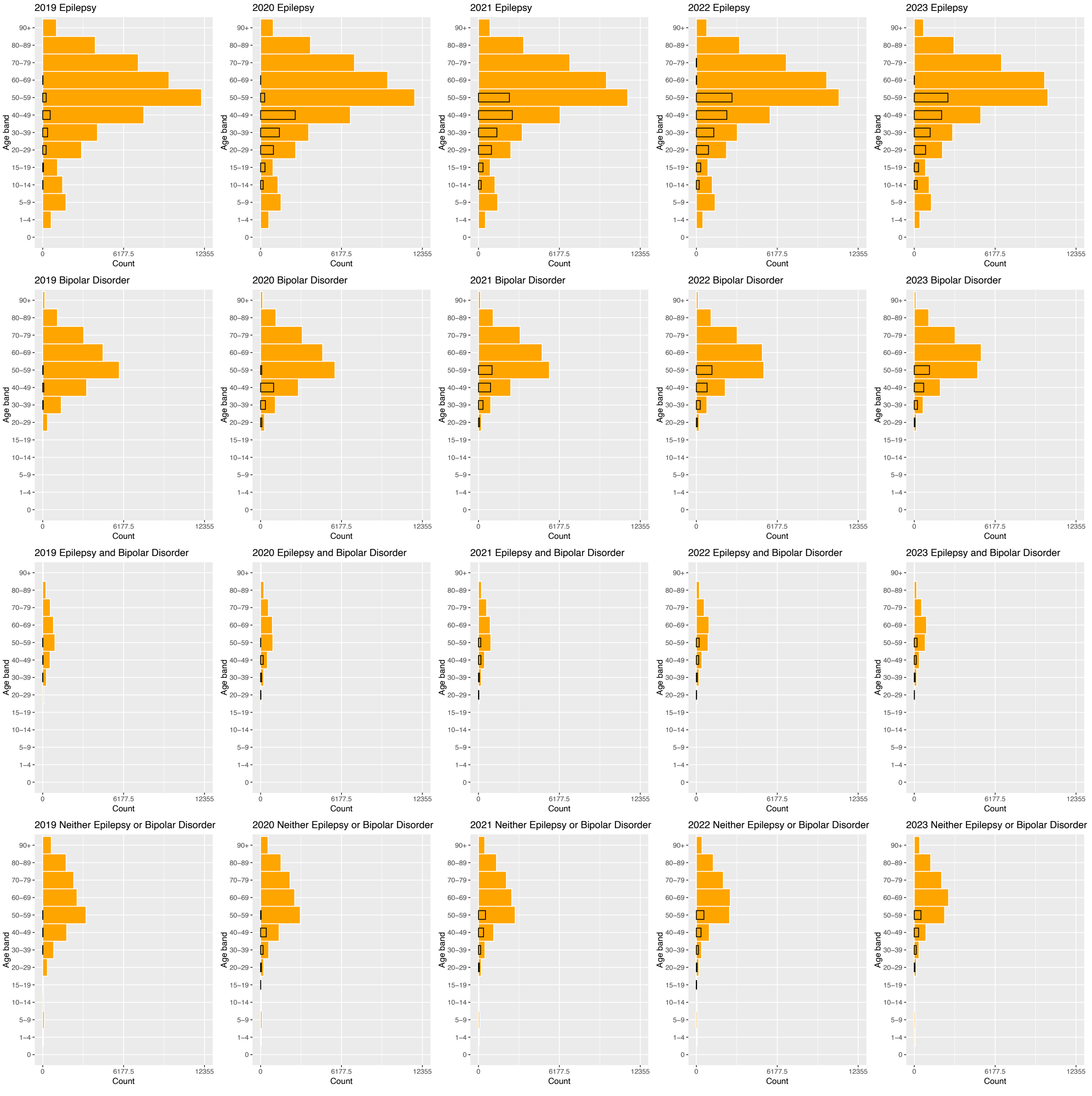

Footnote: Black overlay indicates N of women with any evidence of counselling in their primary care record defined by selected codes (Appendix C)

APPENDICES

Appendix A: Categorisation of included antiepileptic medicines; BNF presentation and codes

Valproate / valproic acid:

Valproic acid 150mg gastro-resistant capsules	040801020AAAAAA
Valproic acid 300mg gastro-resistant capsules	040801020AAABAB
Valproic acid 500mg gastro-resistant capsules	040801020AAACAC
Valproic acid 250mg/5ml oral liquid	040801020AAAFAF
Valproic acid 500mg/5ml oral liquid	040801020AAAGAG
Valproic acid 125mg gastro-resistant capsules	040801020AAAHAH
Convulex 150mg gastro-resistant capsules	040801020BBAAAA
Convulex 300mg gastro-resistant capsules	040801020BBABAB
Convulex 500mg gastro-resistant capsules	040801020BBACAC
Depakote 125mg sprinkle gastro-resistant capsules	040801020BCACAH
SV 300mg modified-release tablets	0408010W0AAA1A1
SV 500mg modified-release tablets	0408010W0AAA2A2
SV 500mg/5ml oral liquid	0408010W0AAA7A7
SV 200mg/5ml oral solution sugar free	0408010W0AAAAAA
SV 100mg tablets	0408010W0AAABAB
SV 200mg gastro-resistant tablets	0408010W0AAACAC
SV 500mg gastro-resistant tablets	0408010W0AAADAD
SV 200mg/5ml oral solution	0408010W0AAAEAE
SV 400mg inj vials	0408010W0AAAHAH
SV 100mg suppositories	0408010W0AAARAR
SV 200mg modified-release tablets	0408010W0AAAZAZ
SV 300mg suppositories	0408010W0AABCBC
SV 600mg/5ml oral solution	0408010W0AABEBE
SV 90mg/5ml oral solution	0408010W0AABFBF
SV 60mg/5ml oral solution	0408010W0AABIBI
SV 125mg/5ml oral solution	0408010W0AABJBJ
SV 20mg/5ml oral solution	0408010W0AABKBK
SV 75mg/5ml oral solution	0408010W0AABLBL
SV 10mg/5ml oral solution	0408010W0AABMBM
SV 300mg/3ml solution for injection ampoules	0408010W0AABPBP
SV 150mg modified-release capsules	0408010W0AABQBQ
SV 300mg modified-release capsules	0408010W0AABRBR
SV 500mg MR gran sachets sugar free	0408010W0AABSBS
SV 1g modified-release gran sachets sugar free	0408010W0AABTBT
SV 1g/10ml solution for injection ampoules	0408010W0AABUBU
SV 50mg MR gran sachets sugar free	0408010W0AABVBV
SV 100mg MR gran sachets sugar free	0408010W0AABWBW
SV 250mg MR gran sachets sugar free	0408010W0AABXBX
SV 750mg MR gran sachets sugar free	0408010W0AABYBY
SV 400mg/4ml solution for injection ampoules	0408010W0AABZBZ
SV 200mg/ml oral solution	0408010W0AACACA
SV 60mg/5ml oral suspension	0408010W0AACBCB

Epilim 100mg crushable tablets	0408010W0BBAAAB
Epilim 200 gastro-resistant tablets	0408010W0BBABAC
Epilim 500 gastro-resistant tablets	0408010W0BBACAD
Epilim 200mg/5ml liquid	0408010W0BBADAA
Epilim 200mg/5ml syrup	0408010W0BBAEAE
Epilim Intravenous 400mg inj vials	0408010W0BBAIAH
Epilim Chrono 200 tablets	0408010W0BBAJAZ
Epilim Chrono 300 tablets	0408010W0BBAKA1
Epilim Chrono 500 tablets	0408010W0BBALA2
Epilim Chronosphere MR 500mg granules sachets	0408010W0BBAMBS
Epilim Chronosphere MR 50mg granules sachets	0408010W0BBANBV
Epilim Chronosphere MR 100mg granules sachets	0408010W0BBAPBW
Epilim Chronosphere MR 250mg granules sachets	0408010W0BBAQBX
Epilim Chronosphere MR 750mg granules sachets	0408010W0BBARBY
Epilim Chronosphere MR 1000mg granules sachets	0408010W0BBASBT
Orlept 200mg gastro-resistant tablets	0408010W0BEAAAC
Orlept 500mg gastro-resistant tablets	0408010W0BEABAD
Orlept SF 200mg/5ml liquid	0408010W0BEACAA
Epival CR 300mg tablets	0408010W0BHAAA1
Epival CR 500mg tablets	0408010W0BHABA2
Episenta 300mg/3ml solution for injection ampoules	0408010W0BIAABP
Episenta 150mg modified-release capsules	0408010W0BIABBQ
Episenta 300mg modified-release capsules	0408010W0BIACBR
Episenta 500mg modified-release granules sachets	0408010W0BIADBS
Episenta 1000mg modified-release granules sachets	0408010W0BIAEBT
Episenta 1g/10ml solution for injection ampoules	0408010W0BIAFBU
Depakin 200mg/ml oral solution	0408010W0BJAACA
Dyzantil 200mg modified-release tablets	0408010W0BKAAAZ
Dyzantil 300mg modified-release tablets	0408010W0BKABA1
Dyzantil 500mg modified-release tablets	0408010W0BKACA2
Valproic acid 250mg gastro-resistant tablets	0402030Q0AAAAAA
Valproic acid 500mg gastro-resistant tablets	0402030Q0AAABAB
Depakote 250mg gastro-resistant tablets	0402030Q0BBAAAA
Depakote 500mg gastro-resistant tablets	0402030Q0BBABAB
Belvo 250mg gastro-resistant tablets	0402030Q0BCAAAA
Belvo 500mg gastro-resistant tablets	0402030Q0BCABAB
Syonell 500mg gastro-resistant tablets	0402030Q0BDAAAB
Syonell 250mg gastro-resistant tablets	0402030Q0BDABAA

Lamotrigine:

Lamictal 100mg dispersible tablets	0408010H0BBAFAW
Lamictal 100mg tablets	0408010H0BBABAA
Lamictal 200mg tablets	0408010H0BBAGA1
Lamictal 25mg dispersible tablets	0408010H0BBAEAQ
Lamictal 25mg tablets	0408010H0BBACAC

Lamictal 2mg dispersible tablets	0408010H0BBAKBJ
Lamictal 50mg tablets	0408010H0BBAAAB
Lamictal 5mg dispersible tablets	0408010H0BBADAP
Lamotrigine 1.5mg/5ml oral liquid	0408010H0AABMBM
Lamotrigine 1.7mg/5ml oral liquid	0408010H0AABSBS
Lamotrigine 100mg dispersible tablets sugar free	0408010H0AAAWAW
Lamotrigine 100mg tablets	0408010H0AAAAAA
Lamotrigine 10mg/5ml oral suspension	0408010H0AABUBU
Lamotrigine 15mg/5ml oral liquid	0408010H0AAAZAZ
Lamotrigine 200mg dispersible tablets sugar free	0408010H0AABQBQ
Lamotrigine 200mg tablets	0408010H0AAA1A1
Lamotrigine 200mg/5ml oral liquid	0408010H0AABGBG
Lamotrigine 20mg/5ml oral suspension	0408010H0AABNBN
Lamotrigine 25mg dispersible tablets sugar free	0408010H0AAAQAQ
Lamotrigine 25mg tablets	0408010H0AAACAC
Lamotrigine 25mg/5ml oral liquid	0408010H0AABRBR
Lamotrigine 2mg dispersible tablets sugar free	0408010H0AABJBJ
Lamotrigine 37.5mg/5ml oral liquid	0408010H0AAA4A4
Lamotrigine 5.5mg/5ml oral liquid	0408010H0AAA5A5
Lamotrigine 50mg dispersible tablets sugar free	0408010H0AABPBP
Lamotrigine 50mg tablets	0408010H0AAABAB
Lamotrigine 50mg/5ml oral suspension	0408010H0AAA3A3
Lamotrigine 5mg dispersible tablets sugar free	0408010H0AAAPAP
Lamotrigine 5mg/5ml oral liquid	0408010H0AABEBE
Lamotrigine 60mg/5ml oral liquid	0408010H0AABTBT
Lamotrigine 70mg/5ml oral suspension	0408010H0AABVBV

Levetiracetam and like compounds:

Brivaracetam 100mg tablets	0408010ALAAAEAE
Brivaracetam 10mg tablets	0408010ALAAAAAA
Brivaracetam 25mg tablets	0408010ALAAABAB
Brivaracetam 50mg tablets	0408010ALAAACAC
Brivaracetam 50mg/5ml oral solution sugar free	0408010ALAAAGAG
Brivaracetam 50mg/5ml solution for injection vials	0408010ALAAAFAF
Brivaracetam 75mg tablets	0408010ALAAADAD
Briviact 100mg tablets	0408010ALBBAEAE
Briviact 10mg tablets	0408010ALBBAAAA
Briviact 10mg/ml oral solution	0408010ALBBAGAG
Briviact 25mg tablets	0408010ALBBABAB
Briviact 50mg tablets	0408010ALBBACAC
Briviact 50mg/5ml solution for injection vials	0408010ALBBAFAF
Briviact 75mg tablets	0408010ALBBADAD
Desitrend 1000mg granules sachets	0408010A0BDACAM
Desitrend 100mg/ml oral solution	0408010A0BDADAH
Desitrend 250mg granules sachets	0408010A0BDAAAK

Desitrend 500mg granules sachets	0408010A0BDABAL
Desitrend 500mg/5ml concentrate for inf ampoules	0408010A0BDAEAN
Genlev 100mg/ml oral solution	0408010A0BEAAAH
Keppra 100mg/ml oral solution	0408010A0BBADAH
Keppra 1g tablets	0408010A0BBACAC
Keppra 250mg tablets	0408010A0BBAAAA
Keppra 500mg tablets	0408010A0BBABAB
Keppra 500mg/5ml concentrate for solution for infusion vials	0408010A0BBAFAJ
Keppra 750mg tablets	0408010A0BBAEAI
Kevesy 1.5g/100ml infusion bags	0408010A0BFACAR
Kevesy 1g/100ml infusion bags	0408010A0BFABAQ
Kevesy 500mg/100ml infusion bags	0408010A0BFAAAP
Levetiracetam 1.5g/100ml infusion bags	0408010A0AAARAR
Levetiracetam 100mg/ml oral solution sugar free	0408010A0AAAHAH
Levetiracetam 1g granules sachets sugar free	0408010A0AAAMAM
Levetiracetam 1g tablets	0408010A0AAACAC
Levetiracetam 1g/100ml infusion bags	0408010A0AAAQAQ
Levetiracetam 250mg granules sachets sugar free	0408010A0AAAKAK
Levetiracetam 250mg tablets	0408010A0AAAAAA
Levetiracetam 250mg/5ml oral liquid	0408010A0AAAFAF
Levetiracetam 300mg/5ml oral liquid	0408010A0AAADAD
Levetiracetam 400mg/5ml oral liquid	0408010A0AAAEAE
Levetiracetam 500mg granules sachets sugar free	0408010A0AAALAL
Levetiracetam 500mg tablets	0408010A0AAABAB
Levetiracetam 500mg/100ml infusion bags	0408010A0AAAPAP
Levetiracetam 500mg/5ml solution for infusion ampoules	0408010A0AAANAN
Levetiracetam 500mg/5ml solution for infusion vials	0408010A0AAAJAJ
Levetiracetam 750mg tablets	0408010A0AAAIAI
Matever 1g tablets	0408010A0BCAAAC
Matever 250mg tablets	0408010A0BCADAA
Matever 500mg tablets	0408010A0BCACAB
Matever 500mg/5ml concentrate for inf vials	0408010A0BCAEAJ
Matever 750mg tablets	0408010A0BCABAI

Topiramate:

Topamax 100mg tablets	040801050BBABAB
Topamax 15mg sprinkle capsules	040801050BBAEAU
Topamax 200mg tablets	040801050BBACAC
Topamax 25mg sprinkle capsules	040801050BBAFAV
Topamax 25mg tablets	040801050BBADAD
Topamax 50mg sprinkle capsules	040801050BBAGAW
Topamax 50mg tablets	040801050BBAAAA
Topiramate 100mg tablets	040801050AAABAB
Topiramate 100mg/5ml oral solution	040801050AACCCC
Topiramate 100mg/5ml oral suspension	040801050AAAZAZ

Topiramate 100mg/5ml oral suspension sugar free	040801050AACHCH
Topiramate 10mg/5ml oral liquid	040801050AAAQAQ
Topiramate 12.5mg/5ml oral liquid	040801050AAAXAX
Topiramate 125mg/5ml oral liquid	040801050AAAYAY
Topiramate 12mg/5ml oral liquid	040801050AABPBP
Topiramate 150mg/5ml oral liquid	040801050AABSBS
Topiramate 15mg capsules	040801050AAAUAU
Topiramate 15mg/5ml oral liquid	040801050AABVBV
Topiramate 16mg/5ml oral liquid	040801050AABTBT
Topiramate 200mg tablets	040801050AAACAC
Topiramate 200mg/5ml oral liquid	040801050AABABA
Topiramate 20mg/5ml oral liquid	040801050AABDBD
Topiramate 250mg/5ml oral suspension	040801050AABZBZ
Topiramate 25mg capsules	040801050AAAVAV
Topiramate 25mg tablets	040801050AAADAD
Topiramate 25mg/5ml oral solution	040801050AACECE
Topiramate 25mg/5ml oral suspension	040801050AABXBX
Topiramate 30mg/5ml oral liquid	040801050AABLBL
Topiramate 35mg/5ml oral liquid	040801050AABRBR
Topiramate 50mg capsules	040801050AAAWAW
Topiramate 50mg tablets	040801050AAAAAA
Topiramate 50mg/5ml oral suspension	040801050AAARAR
Topiramate 50mg/5ml oral suspension sugar free	040801050AACGCG
Topiramate 5mg/5ml oral liquid	040801050AABIBI
Topiramate 6.25mg/5ml oral liquid	040801050AABHBH
Topiramate 62.5mg/5ml oral suspension	040801050AACDCD
Topiramate 67.5mg/5ml oral liquid	040801050AABUBU
Topiramate 75mg/5ml oral liquid	040801050AAASAS
Topiramate 8mg/5ml oral suspension	040801050AACFCF

Carbamazipine and like compounds:

Arbil MR 200mg tablets	0408010C0BGAAAG
Arbil MR 400mg tablets	0408010C0BGABAH
Carbagen 100mg tablets	0408010C0BFACAB
Carbagen 200mg tablets	0408010C0BFADAC
Carbagen 400mg tablets	0408010C0BFAEAD
Carbagen SR 200mg tablets	0408010C0BFAAAG
Carbagen SR 400mg tablets	0408010C0BFABAH
Carbamazepine 100mg chewable tablets sugar free	0408010C0AAAJAJ
Carbamazepine 100mg tablets	0408010C0AAABAB
Carbamazepine 100mg/5ml oral liquid	0408010C0AABDBD
Carbamazepine 100mg/5ml oral suspension sugar free	0408010C0AAAPAP
Carbamazepine 10mg/5ml oral liquid	0408010C0AABGBG
Carbamazepine 120mg/5ml oral liquid	0408010C0AABFBF
Carbamazepine 125mg suppositories	0408010C0AAAUAU

Carbamazepine 200mg chewable tablets sugar free	0408010C0AAAKAK
Carbamazepine 200mg modified-release tablets	0408010C0AAAGAG
Carbamazepine 200mg tablets	0408010C0AAACAC
Carbamazepine 200mg/5ml oral liquid	0408010C0AAAXAX
Carbamazepine 250mg suppositories	0408010C0AAAVAV
Carbamazepine 35mg/5ml oral liquid	0408010C0AABEBE
Carbamazepine 400mg modified-release tablets	0408010C0AAAHAH
Carbamazepine 400mg tablets	0408010C0AAADAD
Carbamazepine 40mg/5ml oral suspension	0408010C0AABHBH
Carbamazepine 500mg/5ml oral suspension	0408010C0AAAYAY
Carbamazepine 50mg/5ml oral solution	0408010C0AABIBI
Epimaz 100mg tablets	0408010C0BCAAAB
Epimaz 200mg tablets	0408010C0BCABAC
Epimaz 400mg tablets	0408010C0BCACAD
Epimaz Retard 200mg tablets	0408010C0BCADAG
Epimaz Retard 400mg tablets	0408010C0BCAEAH
Eslicarbazepine 200mg tablets	0408010AIAAABAB
Eslicarbazepine 50mg/1ml oral suspension sugar free	0408010AIAAACAC
Eslicarbazepine 800mg tablets	0408010AIAAAAAA
Oxcarbazepine 150mg tablets	0408010D0AAACAC
Oxcarbazepine 300mg tablets	0408010D0AAABAB
Oxcarbazepine 400mg/5ml oral suspension	0408010D0AAAFAF
Oxcarbazepine 600mg tablets	0408010D0AAADAD
Oxcarbazepine 60mg/ml oral suspension sugar free	0408010D0AAAEAE
Tegretol 100mg Chewtabs	0408010C0BBAGAJ
Tegretol 100mg tablets	0408010C0BBAAAB
Tegretol 100mg/5ml liquid	0408010C0BBADAP
Tegretol 125mg suppositories	0408010C0BBAIAU
Tegretol 200mg Chewtabs	0408010C0BBAHAK
Tegretol 200mg tablets	0408010C0BBABAC
Tegretol 250mg suppositories	0408010C0BBAJAV
Tegretol 400mg tablets	0408010C0BBACAD
Tegretol Prolonged Release 200mg tablets	0408010C0BBAEAG
Tegretol Prolonged Release 400mg tablets	0408010C0BBAFAH
Teril Retard 200mg tablets	0408010C0BEAAAG
Teril Retard 400mg tablets	0408010C0BEABAH
Trileptal 150mg tablets	0408010D0BBABAC
Trileptal 300mg tablets	0408010D0BBAAAB
Trileptal 600mg tablets	0408010D0BBACAD
Trileptal 60mg/ml oral suspension	0408010D0BBADAE
Zebinix 200mg tablets	0408010AIBBABAB
Zebinix 50mg/1ml oral suspension	0408010AIBBACAC

Appendix B: Disease indication definitions

ICD10 codes:

Disease	ICD10code	ICD10code Description	Category
Epilepsy	G40	Epilepsy	History of Epilepsy
Epilepsy	G41	Status epilepticus	History of Epilepsy

SNOMED-CT codes:

Note not all these codes are active in the selected primary care GDPPR dataset available in the English SDE

conceptId	term
241006	Epilepsia partialis continua
6204001	Juvenile myoclonic epilepsy
7033004	Petit mal status
13973009	Grand mal status
19598007	Generalized epilepsy
28055006	West syndrome
31758001	Postseizure state
44145005	Benign Rolandic epilepsy
50866000	Childhood absence epilepsy
57935008	Epileptic aura
65120008	Generalized convulsive epilepsy
73495003	Dyssynergia cerebellaris myoclonica
79745005	Reflex epilepsy
89525009	Gelastic epilepsy
95208000	Photogenic epilepsy
84757009	Epilepsy
161480008	H/O: epilepsy
163591007	O/E - petit mal fit
163593005	O/E - psychomotor fit
163594004	O/E - salaam attack
170706008	Epilepsy associated problems
170710006	Epilepsy treatment changed
170711005	Epilepsy treatment started
192845009	Myoclonic encephalopathy
192979009	Generalized non-convulsive epilepsy
192981006	Epileptic seizures - atonic
192982004	Epileptic seizures - akinetic
192990004	Benign myoclonic epilepsy in infancy
192991000	Epileptic seizures - clonic
192992007	Epileptic seizures - myoclonic
192993002	Epileptic seizures - tonic
192999003	Partial epilepsy with impairment of consciousness
193000002	Temporal lobe epilepsy
193002005	Psychosensory epilepsy
193003000	Mesiobasal limbic epilepsy
193004006	Epileptic automatism
193008009	Somatosensory epilepsy
193009001	Partial epilepsy with autonomic symptoms

193010006	Visual reflex epilepsy
193011005	Unilateral epilepsy
193017009	Kojevnikov's epilepsy
193021002	Cursive (running) epilepsy
193022009	Localization-related(focal)(partial)idiopathic epilepsy and epileptic syndromes with seizures of localized onset
230290000	Epileptic dementia
230381009	Localisation-related epilepsy
230387008	Benign occipital epilepsy of childhood - early onset variant
230413002	Juvenile absence epilepsy
230418006	Lennox-Gastaut syndrome
230423006	Unverricht-Lundborg syndrome
230429005	Early infantile epileptic encephalopathy with suppression bursts
230437002	Severe myoclonic epilepsy in infancy
230438007	Acquired epileptic aphasia
230441003	Drug-induced epilepsy
230444006	Menstrual epilepsy
230445007	Nocturnal epilepsy
230456007	Status epilepticus
230460005	Complex partial status epilepticus
267581004	Progressive myoclonic epilepsy
307356008	Motor epilepsy
307357004	Jacksonian, focal or motor epilepsy
313307000	Epileptic seizure
314827004	Epilepsy control good
314828009	Epilepsy control poor
352818000	Tonic-clonic epilepsy
361123003	Psychomotor epilepsy
361268000	Alcohol-induced epilepsy
395689002	Transient epileptic amnesia (finding)
401062003	Epilepsy medication review (procedure)
401178003	Epilepsy care arrangement (finding)
407585000	Seizure free > 12 months (finding)
407616001	Epilepsy severity (finding)
407617005	No seizures on treatment (finding)
407618000	1 to 12 seizures a year (finding)
407619008	2 to 4 seizures a month (finding)
407620002	1 to 7 seizures a week (finding)
407621003	Daily seizures (finding)
407622005	Many seizures a day (finding)
407623000	Emergency epilepsy treatment since last appointment (finding)
407627004	Epilepsy confirmed (finding)
407675009	Complex partial epileptic seizure (disorder)
413101007	Stress-induced epilepsy (disorder)
414860000	No epilepsy drug side effects (finding)
416090009	Epilepsy monitoring verbal invite (procedure)
439248002	Epilepsy assessment (regime/therapy)
290671000119100	Status epilepticus due to complex partial epileptic seizure
82381000119103	Epileptic dementia with behavioral disturbance

ICD10 codes:

Disease	ICD10code	ICD10code Description	Category
Bipolar affective disorder and mania	F30	Manic episode	Diagnosis of Bipolar affective disorder and mania
Bipolar affective disorder and mania	F31	Bipolar affective disorder	Diagnosis of Bipolar affective disorder and mania

SNOMED-CT codes:
Note not all these codes are active in the selected primary care GDPPR dataset available in the English SDE

conceptId	term
162004	Severe manic bipolar I disorder without psychotic features
4441000	Severe bipolar disorder with psychotic features
5703000	Bipolar disorder in partial remission
13313007	Mild bipolar disorder
13746004	Bipolar disorder
16506000	Mixed bipolar I disorder
28663008	Severe manic bipolar I disorder with psychotic features
31446002	Bipolar I disorder, most recent episode hypomanic
41836007	Bipolar disorder in full remission
49512000	Depressed bipolar I disorder in partial remission
53049002	Severe bipolar disorder without psychotic features
53607008	Depressed bipolar I disorder in remission
61403008	Severe depressed bipolar I disorder without psychotic features
63249007	Manic bipolar I disorder in partial remission
79584002	Moderate bipolar disorder
83225003	Bipolar II disorder
85248005	Bipolar disorder in remission
191580002	Affective psychoses (& [bipolar] or [depressive] or [manic])
191583000	Single manic episode, mild
191584006	Single manic episode, moderate
191586008	Single manic episode, severe, with psychosis
191588009	Single manic episode in full remission
191590005	Recurrent manic episodes
191592002	Recurrent manic episodes, mild
191593007	Recurrent manic episodes, moderate
191595000	Recurrent manic episodes, severe, with psychosis
191597008	Recurrent manic episodes, in full remission
191618007	Bipolar affective disorder, current episode manic
191620005	Bipolar affective disorder, currently manic, mild
191621009	Bipolar affective disorder, currently manic, moderate
191623007	Bipolar affective disorder, currently manic, severe, with psychosis
191625000	Bipolar affective disorder, currently manic, in full remission
191627008	Bipolar affective disorder, current episode depression
191629006	Bipolar affective disorder, currently depressed, mild
191630001	Bipolar affective disorder, currently depressed, moderate
191632009	Bipolar affective disorder, currently depressed, severe, with psychosis
191634005	Bipolar affective disorder, currently depressed, in full remission
191636007	Mixed bipolar affective disorder
191638008	Mixed bipolar affective disorder, mild
191639000	Mixed bipolar affective disorder, moderate
191641004	Mixed bipolar affective disorder, severe, with psychosis

191643001	Mixed bipolar affective disorder, in full remission
191658009	Atypical manic disorder
192362008	Bipolar affective disorder , current episode mixed
231494001	Mania
231495000	Manic stupor
231496004	Hypomania
268619003	Manic disorder, single episode
371596008	Bipolar I disorder (disorder)
405273008	Manic mood (finding)
765176007	Psychosis and severe depression co-occurrent and due to bipolar affective disorder

Appendix C: Counselling/ pregnancy advice for people with epilepsy

Definition from dss_corporate.gdppr_cluster_refset where Cluster ID = 'EPILCC_COD'

Cluster_ID	Cluster_Desc	Refset_Description	ConceptId	ConceptId_Description
EPILCC_COD	Contraceptive counselling codes for people with epilepsy	United Kingdom National Health Service primary care data extraction - General practice data extraction - contraceptive counselling for epilepsy done simple reference set (foundation metadata concept)	526921000000102	Contraceptive advice for patients with epilepsy (procedure)
EPILPA_COD	Pregnancy advice codes for people with epilepsy	United Kingdom National Health Service primary care data extraction - General practice data extraction - pregnancy advice for epilepsy given simple reference set (foundation metadata concept)	526961000000105	Pregnancy advice for patients with epilepsy (procedure)
EPILPCA_COD	Pre-conception advice codes for people with epilepsy	United Kingdom National Health Service primary care data extraction - General practice data extraction - pre conception advice for epilepsy given simple reference set (foundation metadata concept)	526941000000109	Pre-conception advice for patients with epilepsy (procedure)

Appendix D: List and source of included pregnancy codes

Pregnancy codes are drawn from multiple sources:

1) "Pregnancy" PH348. HDRUK Phenotype library. https://phenotypes.healthdatagateway.org/phenotypes/PH348/version/1512/detail/. (Authors: Julie George, Emily Herrett, Liam Smeeth, Harry Hemingway, Anoop Shah, Spiros Denaxas). Mapped from Read v2 to SNOMED.

2) dss_corporate.gdppr_cluster_refset where Cluster_ID = 'C19PREG_COD'

cluster_id	cluster_desc	conceptid	conceptid_description
C19PREG_COD	Pregnant patients at any stage of pregnancy	10742121000119100	Asthma in mother complicating childbirth (disorder)
C19PREG_COD	Pregnant patients at any stage of pregnancy	169488004	Contraceptive intrauterine device failure - pregnant (finding)
C19PREG_COD	Pregnant patients at any stage of pregnancy	169501005	Pregnant diaphragm failure (finding)
C19PREG_COD	Pregnant patients at any stage of pregnancy	169508004	Pregnant sheath failure (finding)
C19PREG_COD	Pregnant patients at any stage of pregnancy	169560008	Pregnant - urine test confirms (finding)
C19PREG_COD	Pregnant patients at any stage of pregnancy	169561007	Pregnant - blood test confirms (finding)
C19PREG_COD	Pregnant patients at any stage of pregnancy	169562000	Pregnant - vaginal examination confirms (finding)
C19PREG_COD	Pregnant patients at any stage of pregnancy	169564004	Pregnant - on abdominal palpation (finding)
C19PREG_COD	Pregnant patients at any stage of pregnancy	169565003	Pregnant - planned (finding)
C19PREG_COD	Pregnant patients at any stage of pregnancy	169566002	Pregnancy unplanned but wanted (finding)
C19PREG_COD	Pregnant patients at any stage of pregnancy	413567003	Aplastic anemia associated with pregnancy (disorder)
C19PREG_COD	Pregnant patients at any stage of pregnancy	72301000119103	Asthma in pregnancy (disorder)
C19PREG_COD	Pregnant patients at any stage of pregnancy	77386006	Pregnant (finding)
C19PREG_COD	Pregnant patients at any stage of pregnancy	91948008	Asymptomatic human immunodeficiency virus infection in pregnancy (disorder)
C19PREG_COD	Pregnant patients at any stage of pregnancy	10231000132102	In-vitro fertilisation pregnancy (finding)
C19PREG_COD	Pregnant patients at any stage of pregnancy	1148801000000110	Monochorionic monoamniotic triplet pregnancy (disorder)
C19PREG_COD	Pregnant patients at any stage of pregnancy	1148811000000100	Trichorionic triamniotic triplet pregnancy (disorder)
C19PREG_COD	Pregnant patients at any stage of pregnancy	1148821000000100	Dichorionic triamniotic triplet pregnancy (disorder)
C19PREG_COD	Pregnant patients at any stage of pregnancy	1148841000000110	Dichorionic diamniotic triplet pregnancy (disorder)
C19PREG_COD	Pregnant patients at any stage of pregnancy	1149411000000100	Monochorionic diamniotic triplet pregnancy (disorder)
C19PREG_COD	Pregnant patients at any stage of pregnancy	1149421000000110	Monochorionic triamniotic triplet pregnancy (disorder)
C19PREG_COD	Pregnant patients at any stage of pregnancy	134781000119106	High risk pregnancy due to recurrent miscarriage (finding)
C19PREG_COD	Pregnant patients at any stage of pregnancy	16356006	Multiple pregnancy (disorder)
C19PREG_COD	Pregnant patients at any stage of pregnancy	169563005	Pregnant - on history (finding)
C19PREG_COD	Pregnant patients at any stage of pregnancy	237238006	Pregnancy with uncertain dates (finding)
C19PREG_COD	Pregnant patients at any stage of pregnancy	237239003	Low risk pregnancy (finding)
C19PREG_COD	Pregnant patients at any stage of pregnancy	276367008	Wanted pregnancy (finding)
C19PREG_COD	Pregnant patients at any stage of pregnancy	314204000	Early stage of pregnancy (finding)
C19PREG_COD	Pregnant patients at any stage of pregnancy	439311009	Intends to continue pregnancy (finding)

C19PREG_COD	Pregnant patients at any stage of pregnancy	444661007	High risk pregnancy due to history of preterm labor (finding)
C19PREG_COD	Pregnant patients at any stage of pregnancy	459166009	Dichorionic diamniotic twin pregnancy (disorder)
C19PREG_COD	Pregnant patients at any stage of pregnancy	459167000	Monochorionic twin pregnancy (disorder)
C19PREG_COD	Pregnant patients at any stage of pregnancy	459168005	Monochorionic diamniotic twin pregnancy (disorder)
C19PREG_COD	Pregnant patients at any stage of pregnancy	459171002	Monochorionic monoamniotic twin pregnancy (disorder)
C19PREG_COD	Pregnant patients at any stage of pregnancy	47200007	High risk pregnancy (finding)
C19PREG_COD	Pregnant patients at any stage of pregnancy	60810003	Quadruplet pregnancy (disorder)
C19PREG_COD	Pregnant patients at any stage of pregnancy	64254006	Triplet pregnancy (disorder)
C19PREG_COD	Pregnant patients at any stage of pregnancy	65147003	Twin pregnancy (disorder)
C19PREG_COD	Pregnant patients at any stage of pregnancy	713575004	Dizygotic twin pregnancy (disorder)
C19PREG_COD	Pregnant patients at any stage of pregnancy	713576003	Monozygotic twin pregnancy (disorder)
C19PREG_COD	Pregnant patients at any stage of pregnancy	72957006	Diamniotic-monochorionic twins (finding)
C19PREG_COD	Pregnant patients at any stage of pregnancy	80997009	Quintuplet pregnancy (disorder)
C19PREG_COD	Pregnant patients at any stage of pregnancy	890096007	Pregnancy with implant contraceptive (finding)

3) Criteria for identifying delivery episodes in HES
(Codes adopted from Blotière et al. 2018 [1])

**Variable used**		**Inclusion Criteria (Value recorded in HES and explanation)**	**Weeks for estimation**
**Diagnostic codes (ICD-10)**	Live births & Stillbirths	Z37: Outcome of delivery	40
		O48: Prolonged pregnancy	42
		O80: Single spontaneous delivery	40
		O81: Single delivery by forceps and vacuum extractor	40
		O82: Single delivery by caesarean section	40
		O83: Other assisted single delivery	40
		O84: Multiple delivery	40
		O60.1: Preterm spontaneous labour with preterm delivery	36+6d
		O60.2: Preterm spontaneous labour with term delivery by caesarean section	36+6d
		O60.3: Preterm delivery without spontaneous labour	36+6d
	Abortion	O03: Spontaneous abortion	23+6d*
		O04: Medical abortion	23+6d*
		O05: Other abortion	23+6d*
		O06: Unspecified abortion	23+6d*
	Ectopic pregnancy	O00: Ectopic pregnancy	12*
	Other	O01: Hydatidiform mole	14*
		O02: Other abnormal products of conception	23+6d*
**Procedure codes (OPCS-4)**	**Delivery**	R17: Elective caesarean delivery	40 (When there is no preterm/post-term delivery code)
		R18: Other caesarean delivery	
		R19: Breech extraction delivery	
		R20: Other breech delivery	
		R21: Forceps cephalic delivery	
		R22: Vacuum delivery	
		R23: Cephalic vaginal delivery with abnormal presentation of head at delivery without instrument	
		R24: Normal delivery	
		R25: Other methods of delivery	
	**Abortion**	Q09.1: Open removal of products of conception from uterus	-
		Q10.1: Dilation of cervix uteri and curettage of products of conception from uterus	-
		Q10.2: Curettage of products of conception from uterus NEC	-
		Q11: Other evacuation of contents of uterus	23+6d**
		Q14: Introduction of abortifacient into uterine cavity	23+6d**
		Q58: Delivery of terminated fetus	-
		R06: Destruction of fetus	-

	**Gestation age**	Y95.1: Over 20 weeks gestational age<br>Y95.2: From 14 weeks to 20 weeks gestational age<br>Y95.3: From 9 weeks to < 14 weeks gestational age<br>Y95.4: Under 9 weeks gestational age<br>Y95.8: Other specified<br>Y95.9: Unspecified	24<br>20<br>13+6d<br>8+6d<br>Based on the diagnosis<br>Based on the diagnosis
**HES Specific Fields**	**Episode type** *(EPITYPE)*	1 = General episode (anything that is not covered by the other codes)<br>2 = Delivery episode<br>5 = Other delivery event	-
	**Sex**	2 = Female	-
	**Admission date** *(ADMIDATE)*	Between 01/2018 and 12/2021	-

*: Only apply when there is no gestation age available (Y95.8/Y95.9/Null)

**: Only apply with the presence of "Z37" code and without preterm/post-term delivery codes.

Supplementary Methods

Data

Motivated by the public health importance of understanding the relationship between COVID-19, cardiovascular diseases and a wide range of other health conditions, the Health Data Research UK (HDR UK) British Heart Foundation (BHF) Data Science Centre (DSC) established the CVD-COVID-UK/COVID-IMPACT consortium and related research programme[41,42]. Through this initiative, linked, nationally-collated electronic health record (EHR) data for the population of England have been made available to support research into the wide ranging direct and indirect impacts of COVID-19 on health. Details of the collaboration and the data included within each of the national TREs are described in full elsewhere (https://bhfdatasciencecentre.org/areas/cvd-covid-uk-covid-impact/)[20]. **Figure 1** details the data included in these analyses. Data processing details for this work are available under an open-source license at https://github.com/BHFDSC/CCU014_03.

Identification of the pregnancy episode

Code definitions considered for pregnancy are included in **Appendix D**. Pregnant codes recorded for males and pregnancies identified to women aged 50+ were excluded.

All HES APC episodes with diagnoses and procedures indicative of the completion of pregnancy were extracted. We limited the records to females and the time period from January 2017 to December 2023 based on their admitted date. Then the selected records were linked to the HES maternity database using the HES Record Identifier. Since HES maternity is the supplementary record of HES APC, one episode of the record shares the same identifier in both datasets. However, records may be out of sync if the pseudonymisation process presents errors when transferring data from NHS England to SDE. Non-linkable identifiers were discarded, which consisted of 1.8% of the HES maternity records. We then de-duplicated the episodes by only selecting the records with the later episode end date or the greater number of identifiers.

Since we selected records based on pregnancy outcomes, we treated episode end date as the end date of pregnancy. Next, the pregnancy start date was defined by subtracting the length of gestation from the pregnancy end date. For those with no information on length of gestation (36.2%), we assigned the weeks according to their OPCS/ICD-10 codes in that episode. For women who had code "Z37" but without gestation weeks information, 40 weeks was assigned unless preterm or prolonged labour code appeared. For preterm delivery, 36 weeks and 6 days was assigned. For prolonged labour, 42 weeks was assigned. For abortions or termination of pregnancy cases, we assigned the week based on their OPCS code of gestation age code. If there was no OPCS codes of gestation age, we assigned the maximal possible weeks of performing the procedure. For example, 23 weeks and 6 days was assigned for abortions and 12 weeks was assigned for ectopic pregnancy. The maximum possible estimated weeks were assigned given the desire to use the pregnancy interval in a safety focussed project - i.e., in order to avoid missing possible pregnancies.

For the remaining potential pregnancies that we were unable to link to HES APC a different approach was taken. Primary care records were ordered by event date and patient ID. The very first pregnancy code in a woman's record was compared to the subsequent pregnancy code, calculating the date difference between the two records. When the difference was great enough (defined below) it was considered as another pregnancy episode. If the pregnancy code was related to postpartum (i.e., codes in the category of labour delivery or condition at birth), the interval with the next record should be 6 weeks apart, since the majority of non-lactating women will not return to ovulation until 6 weeks postpartum, meaning they have a chance to be pregnant again. If the pregnancy code was not related to postpartum, the interval with the next record should be at least 40 weeks apart – the length of a full-term pregnancy. If the subsequent pregnancy code date was <40 weeks, it was considered to belong to the first pregnancy episode. Finally, we estimated the pregnancy period according to the type of code. We assumed that women with codes of pregnancy recognition were 10-weeks pregnant. This assumption is made since most women book their antenatal appointment within 10-week gestation during the period 2018 to 2020 according to NHS England's Maternity Services Monthly Statistics. Therefore, to estimate their pregnancy start date, we subtract 10 weeks from the date of the pregnancy code recorded. We then added 30 weeks to the date of recorded pregnancy code as the end of pregnancy. For pregnancy outcome codes, we took the date recorded as the pregnancy end date. Assuming it was full-term pregnancy, we deducted 40 weeks to determine the pregnancy start date.
