# Supplementary material for: Use of sodium valproate and other antiseizure drug treatments in England and Wales: quantitative analysis of nationwide linked electronic health records
Source: BMJ Med. 2024 Dec 20;3(1):e000760. doi: 10.1136/bmjmed-2023-000760 (PMC12164316; doi:10.1136/bmjmed-2023-000760)
Supplement: online supplemental file 2 [file bmjmed-3-1-s002.pdf]

PROTOCOL: The impact of the COVID-19 pandemic on use of sodium valproate and implementation of recommendations of the Cumberlege report

Authors

Caroline E Dale & Rohan Takhar, Yat Yi Fan, Fatemeh Torabi, Michail Katsoulis, Samuel Kim, Andrew Lambarth, Christopher Tomlinson, Tim Wilkinson, Tanja Mueller, Amanj Kurdi, Mark Ashworth, Mamas A Mamas, Kamlesh Khunti, Ashley Akbari, Andrew D Morris, Munir Pirmohamed, Anthony G Marson, David Williams, David Hunt, Cathie Sudlow, Reecha Sofat, on behalf of the CVD-COVID-UK/COVID-IMPACT Consortium

Version history

Version	Date	Comments
0.1	28/01/21	Initial Draft by RS
0.2	31/01/21	Reviewed by CD
0.3	09/02/21	Updated by CD; codelists added
0.4	22/02/21	Updated following comments in Box
0.5	09/03/22	Rationalisation of objectives for short report (3 nations) focusing on different disease diagnoses (epilepsy, bipolar) and how these may impact rates of counselling and/ or pregnancy
0.6	22/03/22	Changes to dates/code lists following data feasibility checks
0.7	15/05/22	Scotland removed as data not available
0.8	23/06/22	EPMA analysis added
0.9	05/09/22	Link with medicines restricted to within pregnancy episode
1.0	12/10/22	Addition of logistic regression to investigate factors associated with SV use during pregnancy<br>Dose during pregnancy added; analysis by trimester
1.1	07/04/23	Monthly SV counts updated to July 2022
1.2	10/06/23	Analysis of ASMs taken in combination with SV added
1.3	04/07/23	Analysis of epilepsy-related mortality and HES
1.4	31/01/24	Changes in response to BMJ reviewer requests, namely<br>Removing:<br>• Stratifications by ethnicity, geographical region and IMD<br>• Logisitic regression of factors associated with sodium valproate pregnancy<br>• EPMA analyses<br><br>And:<br>• Adding monthly trends of all ASMs by gender<br>• Revision of epilepsy-related mortality models<br><br>Study period updated to end 2023; and pregnancy rates to 2022

Background:

Medicines are the most common intervention for treating disease; however their use is not always without harm. Therefore, the benefits of using medication should always be balanced against potential harm. Reducing harm can be challenging either because those at highest risk cannot be identified, or identification may be at the point of harm, e.g. during hospital admission. However, there are some medicines for which the harm is predictable. Early identification and mitigation of risk can make this harm avoidable. Teratogenic drugs present a particular case where harm results to an embryo or foetus when a medicine is ingested by the mother. Crossing the placenta and uptake by the developing foetus particularly during organogenesis can result in irreversible harm. It is uncertain how the use of high-risk

medicines and related harms has changed during the COVID-19 pandemic, either due to changes in prescription patterns or through reduced monitoring due to disruption of primary care services.

One example of a teratogenic medicine where avoidable harms are well known is sodium valproate. Sodium valproate/valproic acid[1] has notable and severe adverse effects, including the risk of birth defects, developmental disorders, and higher risk of miscarriage[2]. Children born to mothers who took the medicine during pregnancy may have a range of neurodevelopmental effects, including intellectual disability, difficulties with language and memory, and learning and behaviour problems[3]. Labelling of the drug reflects these risks and includes a warning on the risk of birth defects. Sodium valproate is primarily used for the treatment of epilepsy but is used although less commonly for bipolar disorder, migraine, chronic pain and a range of other psychiatric conditions[4]. The Food and Drug Administration (FDA) in the United States (US) advises valproate should not be taken by women of child-bearing age for any condition unless the drug is essential to managing the woman's medical condition[5]. In March 2018 the MHRA in the United Kingdom (UK) endorsed a strengthened regulatory position on valproate medicines in stating that "Valproate must no longer be used in any woman or girl able to have children unless she has a pregnancy prevention programme (PPP) in place"[6, 7]. The PPP is designed to make sure patients are fully aware of the risks and the need to avoid becoming pregnant and includes the completion of a signed risk acknowledgement form when their treatment is reviewed by a specialist, at least annually.

The report by Baroness Cumberlege 'First do no Harm' (published July 2020) highlighted the issue of sodium valproate and the avoidable harm in the UK, including the pain of mothers who took it during pregnancy to manage their seizures without knowing the risks this could pose for their unborn children. The Cumberlege report also highlighted potential differences in implementation of pregnancy/ contraception counselling amongst users with bipolar disorder rather than epilepsy [3, 8]. Other recommended actions included long-term data collection of women on all antiepileptic drugs (and their children) and to establish a registry of all valproate users who become pregnant[9] (see Supplementary Material for full list of recommended actions). One of the challenges to the implementation of the recommendations of this report is the COVID-19 pandemic and the indirect effects it has had on many if not all aspects of healthcare, but perhaps especially the implementation of new policies. Reduced face-to-face interaction between patients and GPs as a result of the pandemic[10] may also have had implications for counselling amongst users of sodium valproate.

Aim:

To demonstrate, using sodium valproate as an example, how national electronic health data can identify indirect effects on the use of harmful medicines, highlighting where and by whom it is being used, drawing on linked socio-demographic and co-morbidity data, and how the COVID-19 pandemic has impacted these trends. This example can also be a conduit to avoiding harm from medicines through the detailed description of their use nationwide and streamlined implementation of actionable analytics within the NHS.

Objectives:

1. To measure the use of sodium valproate (prevalent and incident) and describe how this has changed during the course of the COVID-19 pandemic and/or in response to the Cumberlege report by placing sodium valproate in the context of other commonly used anti-epileptics (ASMs e.g. lamotrigine, levitaracetam, topiramate, carbamazepine).
2. To describe how the use of sodium valproate varies according to socio-demographic measures (geographical region, ethnicity, socioeconomic deprivation, gender, age) and how this compares to other anti-epileptic medicines (medicines-lens focus).
3. To measure the use of sodium valproate by women of child-bearing potential (CBP; age 15-49) and how this compares to the age- and sex- distribution of other anti-epileptic medicines.
4. To measure the rate of pregnancy amongst women of CBP using sodium valproate, how this compares with other ASMs, and if this has changed during the pandemic.
5. To describe the total dose and timing of sodium valproate used during pregnancy
6. To measure the prevalence of diagnosis for different disease indications amongst women of CBP using of sodium valproate (epilepsy, bipolar, both, neither).
7. To measure the rate of counselling (pre-conception/ contraceptive/ pregnancy advice) and if rates have changed over time and/ or differ by disease indication (epilepsy, bipolar, both, neither).
8. To explore any evidence for change in epilepsy-related mortality, stratified by gender.

Methods:

<u>Study Design</u>
This is a descriptive analysis of a potentially harmful medicine use (sodium valproate) over time and by sub-populations before and during the COVID-19 pandemic.

<u>Data Sources</u>
England:
Linked population level National Health Service (NHS) data sources within NHS Digital Trusted Research Environment (TRE) for England. The data sources include:

1. Medicines Dispensing Data: NHS BSA – to define medication population
2. Patient Skinny Table (data derived from GDPPR, HES, ONS deaths) – for linked demographic information
3. GDPPR – for defining indications, concurrent pregnancy, and counselling

Wales:
Linked population level data for Wales accessed through the Trusted Research Environment (TRE) known as SAIL Databank (Lyons et al. 2009). The data sources include:

1. Welsh Dispensing DataSet (WDDS) – to define medication population
2. Welsh Demographic Dataset for defining the demographic details of the cohort
3. Welsh Longitudinal General Practice dataset (WLGP) for defining indications, pregnancy, contraceptive use and pregnancy counselling.
4. We will also derive a pregnancy spine from multiple sources including:
    a. Maternity Indicators Dataset (MIDS)
    b. National community Child Health Database (NCCHD)
    c. Congenital Anomaly Register and Information Service for Wales (CARS)

<u>Study period</u>
NHS BSA data available from 1st April 2018 to 31st December 2023. 1st January 2019 to 31st December 2023 for annual medicines-based analyses (e.g. Objective 2)

<u>Inclusion/Exclusion criteria</u>

Main study population will be defined based on all those who had at least one dispensed prescription of interest between 1st January 2019 to 31st December 2023. 2018 data will be included for incident analyses.

For England: All records of dispensed medications with LSOA=E (England) during the study period will be included.
For Wales: All records of dispensed medications with LSOA=W (Wales) during the study period will be included.

For all records across two nations, records with null IDs will be excluded from linked analyses based on ID, e.g. ethnicity, incidence. Methods will be applied to include additional medicines with null IDs but data for age and sex (see below for details).

<u>Analysis pipeline</u>

1) Sodium valproate and other anti-epileptic medicines will be categorised by BNF codes and identified in the NHS BSA dispensing data (**Appendix Table A**).

2) Total medicines volume (by year, month) will be counted by anti-epileptic category for years 2019,2020,2021,2022,2023 as well as annual counts of the population (distinct individuals using ID) receiving medicines for years 2019,2020,2021,2022,2023.

3) Medicines counts and populations will further be stratified by socio-demographic variables:

- o Age band (5-year bands for ages 0-19 years; 10-year age bands ages 20 years-90+). For England, the consortium patient "skinny" table will be used as the primary source for this information. Note individuals transitioning between age bands within a given calendar year (e.g. from age 59 to 60) will be assigned to the lower age band 50-59 for the duration of the calendar year to avoid double counting of these individuals. For Wales, C20 cohort will be used (Lyons et al. 2020). For dispensed medication records with null IDs (i.e. where linkage is not possible), age will be recovered from the age variable recorded at medicine dispense.
- o Sex. For England, the patient skinny table will be used as the primary source for this information but for dispensed medication records with null IDs (i.e. where linkage is not possible), sex will be recovered from the variable recorded at medicine dispense. For Wales, C20 cohort will be used (Lyons et al. 2020).
- o Geographical region. Linked using dispense LSOA variable (complete). Missing region included as separate category
- o Deprivation will be measured uisng routine deprivation scores measures, England: IMD and Wales: WIMD. Linked using dispense LSOA (complete).
- o Ethnicity. For England: Through linkage to the patient skinny table/medicine records with null IDs will therefore be excluded from this stratification. For Wales, Through linkage to Ethnicity Spine (Akbari et al., 2022).

4) Incidence dispenses for each individual will be identified by ranking within substance and across the entire epileptic category and selecting rank=1 for each and both. 2018 dispenses will be used as a "washing out" window and results presented for 2019, 2020, 2021, 2022.

5) Rates of sodium valproate use by age- and sex- will be calculated using ONS mid-year national population estimates (e.g. for England LSOA staring with E) as the denominator. These will be compared with rates of other anti-epileptic medications over the same period (2019, 2020, 2021, 2022 – once available from ONS). Aggregating LSOA to LAD rates of use of sodium valproate by women of CBP and men of the same age across England will be compared.

6) Amongst women of CBP dispensed SV, we will investigate the most common combinations of other ASMs dispensed in this population for the most recent calendar year of the study period.

7) Rates of different indications (epilepsy, bipolar disorder, both, neither; **Appendix Table D**) will be calculated in women of CBP for years 2019, 2020, 2021, 2022.

8) Primary care records data and Welsh pregnancy spine table will be screened to identify women who have a recorded dispense of sodium valproate dispense during a defined pregnancy episode. Where available, pregnancy episodes will be defined using HES based on evidence of a birth/ mis-carriage/ termination applying appropriate estimation of pregnancy duration. In addition, primary care data will be assessed for evidence of pregnancy. Code definitions for pregnancy are included in **Appendix Table B**. Pregnant codes recorded for males and pregnancies identified by a single code to women aged 50+ will be excluded.

9) Timing of dispense by trimester (first trimester days 1-90 of the pregnancy episode) will be investigated and the quantity (in grams per day) of valproate dispensed during the pregnancy will be calculated.

10) Evidence for counselling (contraception and/ or pregnancy; **Appendix Table C**) in same calendar year as dispense of SV in England 2019-2023 in females by age and disease indication will be explored.

11) Using English mortality data from the ONS available in the SDE, we will extract deaths by month from January 2015 to December 2022 including for ICD-10 codes G40 (Epilepsy & recurrent seizures) & G41 (Status epilepticus) where these codes appeared anywhere on the death certificate. Stratifying by sex, we will fit linear regression models for the periods before and after the MHRA advice in March 2018 recording the slope and 95% confidence intervals from each model. In addition, time-series using autoregressive integrated moving average (ARIMA) models fitted to the period January 2015 to March 2018 will be used to predict the expected trend in epilepsy-related deaths from April 2018 onwards.

Analyses undertaken, but not included in final manscript:

Predictors of pregnancy
Predictors of pregnancy amongst women of CBP dispensed SV will be explored using multivariate logistic regression. Potential predictors include: age band, disease indication, ethnic group, region, index of multiple deprivation (IMD) deciles, receiving PPP and number of concurrent anti-seizure medications taken.

Analysis of medicines prescribed and dispensed in hospital
Secondary care electronic Prescribing and Medicines Administration (ePMA) data available for a subset of hospitals within the English SDE (25; representing approximately 12% of all hospital trusts across England, including data from ~1 million individuals) will be analysed to investigate whether trends in the use of SV in secondary care were similar to those in the community during the pandemic period.

Analysis of epilepsy-related hospital episode statistics
Epilepsy-related hospital admission will be extracted (ICD-10 codes G40 Epilepsy & recurrent seizures & G41 Status epilepticus) and analysed for evidence in trend by month (total admissions and distinct N of individuals admitted).

Stratifications by ethnicity, geographical region and IMD
Removed following suggestions by BMJ reviewers

References:

1. BNF. *Valproate*. Available from: https://bnf.nice.org.uk/interaction/valproate.html.
2. UK Teratology Information Service. *BUMPS. Best use of medicines in pregnancy*. Available from: https://medicinesinpregnancy.org/Medicine--pregnancy/Valproic-acid/.
3. The Independent Medicines and Medical Devices Safety Review and chaired by Baroness Julia Cumberlege, *First Do No Harm- The report of the Independent Medicines and Medical Devices Safety Review, https://www.immdsreview.org.uk/downloads/IMMDSReview_Web.pdf*,. 2020.
4. MHRA, *CPRD STUDY MONITORING THE USE OF VALPROATE IN GIRLS AND WOMEN IN THE UK January 2010 to June 2019, https://assets.publishing.service.gov.uk/government/uploads/system/uploads/attachment_data/file/844070/CPRD_valproate_usage_report-3rd-revision-Nov-19.pdf*, .
5. US Food & Drug Administration. *FDA Drug Safety Communication: Valproate Anti-seizure Products Contraindicated for Migraine Prevention in Pregnant Women due to Decreased IQ Scores in Exposed Children*. Available from: https://www.fda.gov/drugs/drug-safety-and-availability/fda-drug-safety-communication-valproate-anti-seizure-products-contraindicated-migraine-prevention.
6. *Valproate use by women and girls, https://www.gov.uk/guidance/valproate-use-by-women-and-girls*, .
7. *New measures to avoid valproate exposure in pregnancy, https://www.gov.uk/government/news/new-measures-to-avoid-valproate-exposure-in-pregnancy*, .
8. Haskell, H., *Cumberlege review exposes stubborn and dangerous flaws in healthcare.* BMJ, 2020.
9. NHS Digital. *Medicines and Pregnancy Registry: Valproate use in females aged 0 to 54 in England (April 2018 to September 2020), https://digital.nhs.uk/data-and-information/publications/statistical/mi-medicines-and-pregnancy-registry/valproate-use-in-females-aged-0-to-54-in-england-april-2018-to-september-2020*,.
10. Watt, T., E. Kelly, and R. Fisher. *Use of primary care during the COVID-19 pandemic: May 2021 update: Patient-level data analysis of the impact of COVID-19 on primary care activity in England., https://www.health.org.uk/news-and-comment/charts-and-infographics/use-of-primary-care-during-the-covid-19-pandemic-may-2021*.

Appendix A: Categorisation of included anti-epileptic medicines; BNF presentation and codes

Valproate / valproic acid:

Valproic acid 150mg gastro-resistant capsules	040801020AAAAAA
Valproic acid 300mg gastro-resistant capsules	040801020AAABAB
Valproic acid 500mg gastro-resistant capsules	040801020AAACAC
Valproic acid 250mg/5ml oral liquid	040801020AAAFAF
Valproic acid 500mg/5ml oral liquid	040801020AAAGAG
Valproic acid 125mg gastro-resistant capsules	040801020AAAHAH
Convulex 150mg gastro-resistant capsules	040801020BBAAAA
Convulex 300mg gastro-resistant capsules	040801020BBABAB
Convulex 500mg gastro-resistant capsules	040801020BBACAC
Depakote 125mg sprinkle gastro-resistant capsules	040801020BCACAH
Sodium valproate 300mg modified-release tablets	0408010W0AAA1A1
Sodium valproate 500mg modified-release tablets	0408010W0AAA2A2
Sodium valproate 500mg/5ml oral liquid	0408010W0AAA7A7
Sodium valproate 200mg/5ml oral solution sugar free	0408010W0AAAAAA
Sodium valproate 100mg tablets	0408010W0AAABAB
Sodium valproate 200mg gastro-resistant tablets	0408010W0AAACAC
Sodium valproate 500mg gastro-resistant tablets	0408010W0AAADAD
Sodium valproate 200mg/5ml oral solution	0408010W0AAAEAE
Sodium valproate 400mg inj vials	0408010W0AAAHAH
Sodium valproate 100mg suppositories	0408010W0AAARAR
Sodium valproate 200mg modified-release tablets	0408010W0AAAZAZ
Sodium valproate 300mg suppositories	0408010W0AABCBC
Sodium valproate 600mg/5ml oral solution	0408010W0AABEBE
Sodium valproate 90mg/5ml oral solution	0408010W0AABFBF
Sodium valproate 60mg/5ml oral solution	0408010W0AABIBI
Sodium valproate 125mg/5ml oral solution	0408010W0AABJBJ
Sodium valproate 20mg/5ml oral solution	0408010W0AABKBK
Sodium valproate 75mg/5ml oral solution	0408010W0AABLBL
Sodium valproate 10mg/5ml oral solution	0408010W0AABMBM
Sodium valproate 300mg/3ml solution for injection ampoules	0408010W0AABPBP
Sodium valproate 150mg modified-release capsules	0408010W0AABQBQ
Sodium valproate 300mg modified-release capsules	0408010W0AABRBR
Sodium valproate 500mg MR gran sachets sugar free	0408010W0AABSBS
Sodium valproate 1g modified-release gran sachets sugar free	0408010W0AABTBT
Sodium valproate 1g/10ml solution for injection ampoules	0408010W0AABUBU
Sodium valproate 50mg MR gran sachets sugar free	0408010W0AABVBV
Sodium valproate 100mg MR gran sachets sugar free	0408010W0AABWBW
Sodium valproate 250mg MR gran sachets sugar free	0408010W0AABXBX
Sodium valproate 750mg MR gran sachets sugar free	0408010W0AABYBY
Sodium valproate 400mg/4ml solution for injection ampoules	0408010W0AABZBZ
Sodium valproate 200mg/ml oral solution	0408010W0AACACA
Sodium valproate 60mg/5ml oral suspension	0408010W0AACBCB
Epilim 100mg crushable tablets	0408010W0BBAAAB

Epilim 200 gastro-resistant tablets	0408010W0BBABAC
Epilim 500 gastro-resistant tablets	0408010W0BBACAD
Epilim 200mg/5ml liquid	0408010W0BBADAA
Epilim 200mg/5ml syrup	0408010W0BBAEAE
Epilim Intravenous 400mg inj vials	0408010W0BBAIAH
Epilim Chrono 200 tablets	0408010W0BBAJAZ
Epilim Chrono 300 tablets	0408010W0BBAKA1
Epilim Chrono 500 tablets	0408010W0BBALA2
Epilim Chronosphere MR 500mg granules sachets	0408010W0BBAMBS
Epilim Chronosphere MR 50mg granules sachets	0408010W0BBANBV
Epilim Chronosphere MR 100mg granules sachets	0408010W0BBAPBW
Epilim Chronosphere MR 250mg granules sachets	0408010W0BBAQBX
Epilim Chronosphere MR 750mg granules sachets	0408010W0BBARBY
Epilim Chronosphere MR 1000mg granules sachets	0408010W0BBASBT
Orlept 200mg gastro-resistant tablets	0408010W0BEAAAC
Orlept 500mg gastro-resistant tablets	0408010W0BEABAD
Orlept SF 200mg/5ml liquid	0408010W0BEACAA
Epival CR 300mg tablets	0408010W0BHAAA1
Epival CR 500mg tablets	0408010W0BHABA2
Episenta 300mg/3ml solution for injection ampoules	0408010W0BIAABP
Episenta 150mg modified-release capsules	0408010W0BIABBQ
Episenta 300mg modified-release capsules	0408010W0BIACBR
Episenta 500mg modified-release granules sachets	0408010W0BIADBS
Episenta 1000mg modified-release granules sachets	0408010W0BIAEBT
Episenta 1g/10ml solution for injection ampoules	0408010W0BIAFBU
Depakin 200mg/ml oral solution	0408010W0BJAACA
Dyzantil 200mg modified-release tablets	0408010W0BKAAAZ
Dyzantil 300mg modified-release tablets	0408010W0BKABA1
Dyzantil 500mg modified-release tablets	0408010W0BKACA2

Lamotrigine:

Lamictal 100mg dispersible tablets	0408010H0BBAFAW
Lamictal 100mg tablets	0408010H0BBABAA
Lamictal 200mg tablets	0408010H0BBAGA1
Lamictal 25mg dispersible tablets	0408010H0BBAEAQ
Lamictal 25mg tablets	0408010H0BBACAC
Lamictal 2mg dispersible tablets	0408010H0BBAKBJ
Lamictal 50mg tablets	0408010H0BBAAAB
Lamictal 5mg dispersible tablets	0408010H0BBADAP
Lamotrigine 1.5mg/5ml oral liquid	0408010H0AABMBM
Lamotrigine 1.7mg/5ml oral liquid	0408010H0AABSBS
Lamotrigine 100mg dispersible tablets sugar free	0408010H0AAAWAW
Lamotrigine 100mg tablets	0408010H0AAAAAA
Lamotrigine 10mg/5ml oral suspension	0408010H0AABUBU
Lamotrigine 15mg/5ml oral liquid	0408010H0AAAZAZ

Lamotrigine 200mg dispersible tablets sugar free	0408010H0AABQBQ
Lamotrigine 200mg tablets	0408010H0AAA1A1
Lamotrigine 200mg/5ml oral liquid	0408010H0AABGBG
Lamotrigine 20mg/5ml oral suspension	0408010H0AABNBN
Lamotrigine 25mg dispersible tablets sugar free	0408010H0AAAQAQ
Lamotrigine 25mg tablets	0408010H0AAACAC
Lamotrigine 25mg/5ml oral liquid	0408010H0AABRBR
Lamotrigine 2mg dispersible tablets sugar free	0408010H0AABJBJ
Lamotrigine 37.5mg/5ml oral liquid	0408010H0AAA4A4
Lamotrigine 5.5mg/5ml oral liquid	0408010H0AAA5A5
Lamotrigine 50mg dispersible tablets sugar free	0408010H0AABPBP
Lamotrigine 50mg tablets	0408010H0AAABAB
Lamotrigine 50mg/5ml oral suspension	0408010H0AAA3A3
Lamotrigine 5mg dispersible tablets sugar free	0408010H0AAAPAP
Lamotrigine 5mg/5ml oral liquid	0408010H0AABEBE
Lamotrigine 60mg/5ml oral liquid	0408010H0AABTBT
Lamotrigine 70mg/5ml oral suspension	0408010H0AABVBV

Levetiracetam and like compounds:

Brivaracetam 100mg tablets	0408010ALAAAEAE
Brivaracetam 10mg tablets	0408010ALAAAAAA
Brivaracetam 25mg tablets	0408010ALAAABAB
Brivaracetam 50mg tablets	0408010ALAAACAC
Brivaracetam 50mg/5ml oral solution sugar free	0408010ALAAAGAG
Brivaracetam 50mg/5ml solution for injection vials	0408010ALAAAFAF
Brivaracetam 75mg tablets	0408010ALAAADAD
Briviact 100mg tablets	0408010ALBBAEAE
Briviact 10mg tablets	0408010ALBBAAAA
Briviact 10mg/ml oral solution	0408010ALBBAGAG
Briviact 25mg tablets	0408010ALBBABAB
Briviact 50mg tablets	0408010ALBBACAC
Briviact 50mg/5ml solution for injection vials	0408010ALBBAFAF
Briviact 75mg tablets	0408010ALBBADAD
Desitrend 1000mg granules sachets	0408010A0BDACAM
Desitrend 100mg/ml oral solution	0408010A0BDADAH
Desitrend 250mg granules sachets	0408010A0BDAAAK
Desitrend 500mg granules sachets	0408010A0BDABAL
Desitrend 500mg/5ml concentrate for inf ampoules	0408010A0BDAEAN
Genlev 100mg/ml oral solution	0408010A0BEAAAH
Keppra 100mg/ml oral solution	0408010A0BBADAH
Keppra 1g tablets	0408010A0BBACAC
Keppra 250mg tablets	0408010A0BBAAAA
Keppra 500mg tablets	0408010A0BBABAB
Keppra 500mg/5ml concentrate for solution for infusion vials	0408010A0BBAFAJ
Keppra 750mg tablets	0408010A0BBAEAI
Kevesy 1.5g/100ml infusion bags	0408010A0BFACAR

Kevesy 1g/100ml infusion bags	0408010A0BFABAQ
Kevesy 500mg/100ml infusion bags	0408010A0BFAAAP
Levetiracetam 1.5g/100ml infusion bags	0408010A0AAARAR
Levetiracetam 100mg/ml oral solution sugar free	0408010A0AAAHAH
Levetiracetam 1g granules sachets sugar free	0408010A0AAAMAM
Levetiracetam 1g tablets	0408010A0AAACAC
Levetiracetam 1g/100ml infusion bags	0408010A0AAAQAQ
Levetiracetam 250mg granules sachets sugar free	0408010A0AAAKAK
Levetiracetam 250mg tablets	0408010A0AAAAAA
Levetiracetam 250mg/5ml oral liquid	0408010A0AAAFAF
Levetiracetam 300mg/5ml oral liquid	0408010A0AAADAD
Levetiracetam 400mg/5ml oral liquid	0408010A0AAAEAE
Levetiracetam 500mg granules sachets sugar free	0408010A0AAALAL
Levetiracetam 500mg tablets	0408010A0AAABAB
Levetiracetam 500mg/100ml infusion bags	0408010A0AAAPAP
Levetiracetam 500mg/5ml solution for infusion ampoules	0408010A0AAANAN
Levetiracetam 500mg/5ml solution for infusion vials	0408010A0AAAJAJ
Levetiracetam 750mg tablets	0408010A0AAAIAI
Matever 1g tablets	0408010A0BCAAAC
Matever 250mg tablets	0408010A0BCADAA
Matever 500mg tablets	0408010A0BCACAB
Matever 500mg/5ml concentrate for inf vials	0408010A0BCAEAJ
Matever 750mg tablets	0408010A0BCABAI

Topiramate:

Topamax 100mg tablets	040801050BBABAB
Topamax 15mg sprinkle capsules	040801050BBAEAU
Topamax 200mg tablets	040801050BBACAC
Topamax 25mg sprinkle capsules	040801050BBAFAV
Topamax 25mg tablets	040801050BBADAD
Topamax 50mg sprinkle capsules	040801050BBAGAW
Topamax 50mg tablets	040801050BBAAAA
Topiramate 100mg tablets	040801050AAABAB
Topiramate 100mg/5ml oral solution	040801050AACCCC
Topiramate 100mg/5ml oral suspension	040801050AAAZAZ
Topiramate 100mg/5ml oral suspension sugar free	040801050AACHCH
Topiramate 10mg/5ml oral liquid	040801050AAAQAQ
Topiramate 12.5mg/5ml oral liquid	040801050AAAXAX
Topiramate 125mg/5ml oral liquid	040801050AAAYAY
Topiramate 12mg/5ml oral liquid	040801050AABPBP
Topiramate 150mg/5ml oral liquid	040801050AABSBS
Topiramate 15mg capsules	040801050AAAUAU
Topiramate 15mg/5ml oral liquid	040801050AABVBV
Topiramate 16mg/5ml oral liquid	040801050AABTBT
Topiramate 200mg tablets	040801050AAACAC
Topiramate 200mg/5ml oral liquid	040801050AABABA

Topiramate 20mg/5ml oral liquid	040801050AABDBD
Topiramate 250mg/5ml oral suspension	040801050AABZBZ
Topiramate 25mg capsules	040801050AAAVAV
Topiramate 25mg tablets	040801050AAADAD
Topiramate 25mg/5ml oral solution	040801050AACECE
Topiramate 25mg/5ml oral suspension	040801050AABXBX
Topiramate 30mg/5ml oral liquid	040801050AABLBL
Topiramate 35mg/5ml oral liquid	040801050AABRBR
Topiramate 50mg capsules	040801050AAAWAW
Topiramate 50mg tablets	040801050AAAAAA
Topiramate 50mg/5ml oral suspension	040801050AAARAR
Topiramate 50mg/5ml oral suspension sugar free	040801050AACGCG
Topiramate 5mg/5ml oral liquid	040801050AABIBI
Topiramate 6.25mg/5ml oral liquid	040801050AABHBH
Topiramate 62.5mg/5ml oral suspension	040801050AACDCD
Topiramate 67.5mg/5ml oral liquid	040801050AABUBU
Topiramate 75mg/5ml oral liquid	040801050AAASAS
Topiramate 8mg/5ml oral suspension	040801050AACFCF

Carbamazipine and like compounds:

Arbil MR 200mg tablets	0408010C0BGAAAG
Arbil MR 400mg tablets	0408010C0BGABAH
Carbagen 100mg tablets	0408010C0BFACAB
Carbagen 200mg tablets	0408010C0BFADAC
Carbagen 400mg tablets	0408010C0BFAEAD
Carbagen SR 200mg tablets	0408010C0BFAAAG
Carbagen SR 400mg tablets	0408010C0BFABAH
Carbamazepine 100mg chewable tablets sugar free	0408010C0AAAJAJ
Carbamazepine 100mg tablets	0408010C0AAABAB
Carbamazepine 100mg/5ml oral liquid	0408010C0AABDBD
Carbamazepine 100mg/5ml oral suspension sugar free	0408010C0AAAPAP
Carbamazepine 10mg/5ml oral liquid	0408010C0AABGBG
Carbamazepine 120mg/5ml oral liquid	0408010C0AABFBF
Carbamazepine 125mg suppositories	0408010C0AAAUAU
Carbamazepine 200mg chewable tablets sugar free	0408010C0AAAKAK
Carbamazepine 200mg modified-release tablets	0408010C0AAAGAG
Carbamazepine 200mg tablets	0408010C0AAACAC
Carbamazepine 200mg/5ml oral liquid	0408010C0AAAXAX
Carbamazepine 250mg suppositories	0408010C0AAAVAV
Carbamazepine 35mg/5ml oral liquid	0408010C0AABEBE
Carbamazepine 400mg modified-release tablets	0408010C0AAAHAH
Carbamazepine 400mg tablets	0408010C0AAADAD
Carbamazepine 40mg/5ml oral suspension	0408010C0AABHBH
Carbamazepine 500mg/5ml oral suspension	0408010C0AAAYAY
Carbamazepine 50mg/5ml oral solution	0408010C0AABIBI

Epimaz 100mg tablets	0408010C0BCAAAB
Epimaz 200mg tablets	0408010C0BCABAC
Epimaz 400mg tablets	0408010C0BCACAD
Epimaz Retard 200mg tablets	0408010C0BCADAG
Epimaz Retard 400mg tablets	0408010C0BCAEAH
Eslicarbazepine 200mg tablets	0408010AIAAABAB
Eslicarbazepine 50mg/1ml oral suspension sugar free	0408010AIAAACAC
Eslicarbazepine 800mg tablets	0408010AIAAAAAA
Oxcarbazepine 150mg tablets	0408010D0AAACAC
Oxcarbazepine 300mg tablets	0408010D0AAABAB
Oxcarbazepine 400mg/5ml oral suspension	0408010D0AAAFAF
Oxcarbazepine 600mg tablets	0408010D0AAADAD
Oxcarbazepine 60mg/ml oral suspension sugar free	0408010D0AAAEAE
Tegretol 100mg Chewtabs	0408010C0BBAGAJ
Tegretol 100mg tablets	0408010C0BBAAAB
Tegretol 100mg/5ml liquid	0408010C0BBADAP
Tegretol 125mg suppositories	0408010C0BBAIAU
Tegretol 200mg Chewtabs	0408010C0BBAHAK
Tegretol 200mg tablets	0408010C0BBABAC
Tegretol 250mg suppositories	0408010C0BBAJAV
Tegretol 400mg tablets	0408010C0BBACAD
Tegretol Prolonged Release 200mg tablets	0408010C0BBAEAG
Tegretol Prolonged Release 400mg tablets	0408010C0BBAFAH
Teril Retard 200mg tablets	0408010C0BEAAAG
Teril Retard 400mg tablets	0408010C0BEABAH
Trileptal 150mg tablets	0408010D0BBABAC
Trileptal 300mg tablets	0408010D0BBAAAB
Trileptal 600mg tablets	0408010D0BBACAD
Trileptal 60mg/ml oral suspension	0408010D0BBADAE
Zebinix 200mg tablets	0408010AIBBABAB
Zebinix 50mg/1ml oral suspension	0408010AIBBACAC

Appendix B: List and source of included pregnancy codes

Multiple definitions of pregnancy will be compiled to include the widest possible range of codes indicating possible pregnancies including pregnancies that end before full-term. Definitions will include:

1) Pregnancy – Source: HDRUK Phenotype library (Authors: Julie George, Emily Herrett, Liam Smeeth, Harry Hemingway, Anoop Shah, Spiros Denaxas) – 1940 entries

This definition will be mapped from Read v2 to SNOMED

2) Definition from dss_corporate.gdppr_cluster_refset where Cluster_ID = 'C19PREG_COD'

cluster_id	cluster_desc	conceptid	conceptid_description
C19PREG_COD	Pregnant patients at any stage of pregnancy	10742121000119100	Asthma in mother complicating childbirth (disorder)
C19PREG_COD	Pregnant patients at any stage of pregnancy	169488004	Contraceptive intrauterine device failure - pregnant (finding)
C19PREG_COD	Pregnant patients at any stage of pregnancy	169501005	Pregnant diaphragm failure (finding)
C19PREG_COD	Pregnant patients at any stage of pregnancy	169508004	Pregnant sheath failure (finding)
C19PREG_COD	Pregnant patients at any stage of pregnancy	169560008	Pregnant - urine test confirms (finding)
C19PREG_COD	Pregnant patients at any stage of pregnancy	169561007	Pregnant - blood test confirms (finding)
C19PREG_COD	Pregnant patients at any stage of pregnancy	169562000	Pregnant - vaginal examination confirms (finding)
C19PREG_COD	Pregnant patients at any stage of pregnancy	169564004	Pregnant - on abdominal palpation (finding)
C19PREG_COD	Pregnant patients at any stage of pregnancy	169565003	Pregnant - planned (finding)
C19PREG_COD	Pregnant patients at any stage of pregnancy	169566002	Pregnancy unplanned but wanted (finding)
C19PREG_COD	Pregnant patients at any stage of pregnancy	413567003	Aplastic anemia associated with pregnancy (disorder)
C19PREG_COD	Pregnant patients at any stage of pregnancy	72301000119103	Asthma in pregnancy (disorder)
C19PREG_COD	Pregnant patients at any stage of pregnancy	77386006	Pregnant (finding)
C19PREG_COD	Pregnant patients at any stage of pregnancy	91948008	Asymptomatic human immunodeficiency virus infection in pregnancy (disorder)
C19PREG_COD	Pregnant patients at any stage of pregnancy	10231000132102	In-vitro fertilization pregnancy (finding)
C19PREG_COD	Pregnant patients at any stage of pregnancy	1148801000000110	Monochorionic monoamniotic triplet pregnancy (disorder)
C19PREG_COD	Pregnant patients at any stage of pregnancy	1148811000000100	Trichorionic triamniotic triplet pregnancy (disorder)
C19PREG_COD	Pregnant patients at any stage of pregnancy	1148821000000100	Dichorionic triamniotic triplet pregnancy (disorder)
C19PREG_COD	Pregnant patients at any stage of pregnancy	1148841000000110	Dichorionic diamniotic triplet pregnancy (disorder)
C19PREG_COD	Pregnant patients at any stage of pregnancy	1149411000000100	Monochorionic diamniotic triplet pregnancy (disorder)
C19PREG_COD	Pregnant patients at any stage of pregnancy	1149421000000110	Monochorionic triamniotic triplet pregnancy (disorder)
C19PREG_COD	Pregnant patients at any stage of pregnancy	134781000119106	High risk pregnancy due to recurrent miscarriage (finding)
C19PREG_COD	Pregnant patients at any stage of pregnancy	16356006	Multiple pregnancy (disorder)
C19PREG_COD	Pregnant patients at any stage of pregnancy	169563005	Pregnant - on history (finding)
C19PREG_COD	Pregnant patients at any stage of pregnancy	237238006	Pregnancy with uncertain dates (finding)

C19PREG_COD	Pregnant patients at any stage of pregnancy	237239003	Low risk pregnancy (finding)
C19PREG_COD	Pregnant patients at any stage of pregnancy	276367008	Wanted pregnancy (finding)
C19PREG_COD	Pregnant patients at any stage of pregnancy	314204000	Early stage of pregnancy (finding)
C19PREG_COD	Pregnant patients at any stage of pregnancy	439311009	Intends to continue pregnancy (finding)
C19PREG_COD	Pregnant patients at any stage of pregnancy	444661007	High risk pregnancy due to history of preterm labor (finding)
C19PREG_COD	Pregnant patients at any stage of pregnancy	459166009	Dichorionic diamniotic twin pregnancy (disorder)
C19PREG_COD	Pregnant patients at any stage of pregnancy	459167000	Monochorionic twin pregnancy (disorder)
C19PREG_COD	Pregnant patients at any stage of pregnancy	459168005	Monochorionic diamniotic twin pregnancy (disorder)
C19PREG_COD	Pregnant patients at any stage of pregnancy	459171002	Monochorionic monoamniotic twin pregnancy (disorder)
C19PREG_COD	Pregnant patients at any stage of pregnancy	47200007	High risk pregnancy (finding)
C19PREG_COD	Pregnant patients at any stage of pregnancy	60810003	Quadruplet pregnancy (disorder)
C19PREG_COD	Pregnant patients at any stage of pregnancy	64254006	Triplet pregnancy (disorder)
C19PREG_COD	Pregnant patients at any stage of pregnancy	65147003	Twin pregnancy (disorder)
C19PREG_COD	Pregnant patients at any stage of pregnancy	713575004	Dizygotic twin pregnancy (disorder)
C19PREG_COD	Pregnant patients at any stage of pregnancy	713576003	Monozygotic twin pregnancy (disorder)
C19PREG_COD	Pregnant patients at any stage of pregnancy	72957006	Diamniotic-monochorionic twins (finding)
C19PREG_COD	Pregnant patients at any stage of pregnancy	80997009	Quintuplet pregnancy (disorder)
C19PREG_COD	Pregnant patients at any stage of pregnancy	890096007	Pregnancy with implant contraceptive (finding)

Using these two definitions 50 SNOMED codes were found to be active in the GDPPR

Appendix C: Contraceptive counselling/ pregnancy advice for people with epilepsy

Consortium definition from dss_corporate.gdppr_cluster_refset where Cluster ID = 'EPILCC_COD'

Cluster_ID	Cluster_Desc	Refset_Description	ConceptId	ConceptId_
EPILCC_COD	Contraceptive counselling codes for people with epilepsy	United Kingdom National Health Service primary care data extraction - General practice data extraction - contraceptive counselling for epilepsy done simple reference set (foundation metadata concept)	526921000000102	Contracept with epilep
EPILPA_COD	Pregnancy advice codes for people with epilepsy	United Kingdom National Health Service primary care data extraction - General practice data extraction - pregnancy advice for epilepsy given simple reference set (foundation metadata concept)	526961000000105	Pregnancy epilepsy (p
EPILPCA_COD	Pre-conception advice codes for people with epilepsy	United Kingdom National Health Service primary care data extraction - General practice data extraction - pre conception advice for epilepsy given simple reference set (foundation metadata concept)	526941000000109	Pre-concep with epilep

Cluster_ID = 'EPILPAEXC_COD'

Cluster_ID	Cluster_Desc	Refset_Description	ConceptId	ConceptId_D
EPILCCEXC_COD	Contraceptive counselling for people with epilepsy inappropriate or declined exception codes	United Kingdom National Health Service primary care data extraction - General practice data extraction - contraceptive counselling for epilepsy declined or not indicated simple reference set (foundation metadata concept)	5.27001E+14	Contraceptive epilepsy not i
EPILCCEXC_COD	Contraceptive counselling for people with epilepsy inappropriate or declined exception codes	United Kingdom National Health Service primary care data extraction - General practice data extraction - contraceptive counselling for epilepsy declined or not indicated simple reference set (foundation metadata concept)	5.27091E+14	Contraceptive epilepsy decl
EPILPAEXC_COD	Pregnancy advice for people with epilepsy inappropriate or declined exception codes	United Kingdom National Health Service primary care data extraction - General practice data extraction - pregnancy advice for epilepsy declined or not indicated simple reference set (foundation metadata concept)	5.27041E+14	Pregnancy ad epilepsy not i
EPILPAEXC_COD	Pregnancy advice for people with epilepsy inappropriate or declined exception codes	United Kingdom National Health Service primary care data extraction - General practice data extraction - pregnancy advice for epilepsy declined or not indicated simple reference set (foundation metadata concept)	5.27131E+14	Pregnancy ad epilepsy decl
EPILPCAEXC_COD	Pre-conception advice inappropriate or declined exception codes	United Kingdom National Health Service primary care data extraction - General practice data extraction - pre conception advice or pre conception advice for epilepsy declined or not indicated simple reference set (foundation metadata concept)	5.27021E+14	Pre-conceptio with epilepsy
EPILPCAEXC_COD	Pre-conception advice inappropriate or declined exception codes	United Kingdom National Health Service primary care data extraction - General practice data extraction - pre conception advice or pre conception advice for epilepsy declined or not indicated simple reference set (foundation metadata concept)	5.27111E+14	Pre-conceptio with epilepsy
EPILPCAEXC_COD	Pre-conception advice inappropriate or declined exception codes	United Kingdom National Health Service primary care data extraction - General practice data extraction - pre conception advice or pre conception advice for epilepsy declined or not indicated simple reference set (foundation metadata concept)	9.06171E+14	Pre-conceptio (situation)

Appendix D: Indications definitions (epilepsy, bipolar)

Epilepsy:
caliber_cprd_epilespsy_snomedct
caliber_icd_epilepsy

Bipolar:
caliber_cprd_bad_snomedct
caliber icd_bad
