# Supplementary material for: Use of sodium valproate and other antiseizure drug treatments in England and Wales: quantitative analysis of nationwide linked electronic health records
Source: BMJ Med. 2024 Dec 20;3(1):e000760. doi: 10.1136/bmjmed-2023-000760 (PMC12164316; doi:10.1136/bmjmed-2023-000760)
Supplement: Supplementary file 3 [file bmjmed-2023-000760.reviewer_comments.pdf]

PEER REVIEW HISTORY

BMJ Medicine publishes all reviews undertaken for accepted manuscripts. Reviewers are asked to complete a checklist review form and are provided with free text boxes to elaborate on their assessment. These free text comments are reproduced below.

ARTICLE DETAILS

**TITLE (PROVISIONAL)**	Evaluating the use of sodium valproate and other anti-seizure medication in nationwide linked electronic health records in England and Wales
**AUTHORS**	Dale, Caroline; Takhar, Rohan; Fan, Yat Yi; Torabi, Fatemeh; Katsoulis, Michail; Kim, Samuel; Lambarth, Andrew; Tomlinson, Christopher; Wilkinson, Tim; Mueller, Tanja; Kurdi, Amanj; Ashworth, Mark; Mamas, Mamas A; Khunti, Kamlesh; Akbari, Ashley; Morris, Andrew D; Pirmohamed, Munir; Marson, Anthony G; Williams, David; Hunt, David; Sudlow, Cathie; Sofat, R.; CVD-COVID-UK/COVID-IMPACT Consortium, on behalf of the

VERSION 1 - REVIEW

**REVIEWER NAME**	Schaffer, Andrea
**REVIEWER AFFILIATION**	University of Oxford Nuffield Department of Primary Care Health Sciences, Bennett Institute of Applied Data Science
**REVIEWER CONFLICT OF INTEREST**	None
**DATE REVIEW RETURNED**	26-Oct-2023

**GENERAL COMMENTS**	This manuscript describes patterns valproate and other antiepileptic dispensing in England and Wales, with a focus on people of child-bearing age. There are a lot of interesting data here, however when reading it I found it a bit difficult to navigate as there are so many different results and analyses. One of the problems with trying to include too much is that you can’t do justice to everything - some of the analyses are only given a few short sentences in the Methods and the Results when they warrant more. I suggest the authors consider paring it down (potentially addressing some aspects in a separate paper), such as focussing on the prescribing patterns analyses only - I think this would make it a much stronger paper.<br><br>1. To help readers navigate this work, this manuscript would benefit from a clear and explicit statement of its aims, including each specific outcome being addressed. Currently, the stated aims are “to understand the use of valproate in women of CBP, and the impact of regulatory changes, including whether the COVID-19 pandemic impacted on the implementation of regulatory advice” But this is a bit too vague – it wasn’t until I got to the Methods that I learned that the authors were also going to be predicting pregnancy, and looking at changes in seizure mortality.<br><br>2. I’m curious why the authors chose to compare counts by age and sex (Figure 3) instead of rates? For instance, the authors state: “Distribution of dispense of SV…was much lower in women of CBP compared to men in the same age bands.” But that may be partially explained by differences in the population at risk - wouldn't a comparison of rates be more appropriate? Differences by sex/age may also be explained by differences in rates of the conditions that these medicines treat. Perhaps a better way to look at this this would be to examine the distribution of ASMs among people dispensed any ASM only.<br><br>3. I have a couple of issues with the logistic regression predicting

	pregnancy. First, the goal of this analysis is not clear, and how it fits in with the stated aims. A clearer statement of the rationale is needed to understand whether the methods used and interpretation of results are appropriate. For instance, the authors state they wanted to "predict" pregnancy, but I suspect the goal was to identify associations, as the approach to model building for these two aims differ (this is covered by: Laubach et al. A biologist's guide to model selection and causal inference. Proc Biol Sci 2021 10.1098/rspb.2020.2815.) Second, the description of this analysis is lacking in detail – over what time period were the predictors measured, in relation to assessment of the outcome (this is particularly important)? How were the potential "predictors" selected? How was the final model chosen? Given these issues, I don't think this analysis contributes much, and the paper would be stronger without it.<br><br>4. For the analysis of epilepsy-related deaths, the outcome is the number of deaths, which is quite a rare event. Therefore, the authors should consider whether a count model (Poisson or negative binomial) more appropriate. As with the logistic regression, I think this analysis is less interesting than the prescribing patterns analysis.<br><br>5. Data on valproate dose is presented in the Results, but I didn't see it mentioned in the Methods, or in the Supplementary Methods either. How was dose ascertained? How complete is that data?<br><br>6. Some of the acronyms don't seem necessary (like SV).<br><br>7. How was the list of ASMs included in the study selected? What about other antiepileptics like gabapentinoids or clonazapem?<br><br>8. The authors use the term "dispenses" as a noun. In my experience working with medicine data, I've not heard that term before – I'm more familiar with "dispensings" (or less commonly, "dispensations"). A quick search of the literature confirms that "dispenses" is uncommon.<br><br>9. In Figure 4, the y-axis says Count, but the title says rate.

**REVIEWER NAME**	Zuccolo, Luisa
**REVIEWER AFFILIATION**	Fondazione Human Technopole, Health Data Science Centre
**REVIEWER CONFLICT OF INTEREST**	I have previously published a paper with the first author of this submitted article (10 years ago or so), and I have more recently contributed as Co-I to a grant submission by the same colleague (not funded).
**DATE REVIEW RETURNED**	21-Nov-2023

**GENERAL COMMENTS**	This study describes time trends in the use of sodium valproate, a known teratogen, and other anti-seizure medications in the English and Welsh general population, by demographic characteristics, and with a focus on women of childbearing age and pregnant women in particular. The authors also estimate whether a prescribing guideline change (in 2018) and more general changes in healthcare systems and access during the COVID-19 pandemic (2020-22) affected anti-epileptic drugs dispensing and/or rates of deaths due to epilepsy. Linked electronic health records covering the entire national populations of England and Wales, many aspects of healthcare utilization, and an 8 year period provide detailed phenotyping and statistical power to the analyses.<br><br>I congratulate the authors on a well powered, well designed and generally well presented piece of research. The scale of the population coverage and clinical importance of the medication(s)

	make this a high impact study. I have some general comments, mainly pertaining to the way that objectives, methods and results are organized, a request to add sensitivity analyses to the mortality trends component and a suggestion to streamline the Discussion. Below the authors can find my general and more specific comments. I have recommended that the paper be accepted following minor revisions.<br><br>General Comments<br>• The Research in context section on page 3 could be shortened considerably, and it is not advisable for this section to include so many acronyms.<br>• The scientific objectives need to be made more explicit: could the authors also separate out descriptive objectives from the inferential analysis estimating the effects of guidelines change and pandemic? In particular, there needs to be more consistency between phrasing of research aims in the last paragraph of the Introduction and analytical strategies used, both throughout the Methods and Results chapters (subheadings relative to each research objective would greatly help here) (eg currently there is no mention of investigating pregnancies).<br>• A clearer link between research objectives and analyses could help understand analytical choices, eg why were men included? What can we learn from the comparison between men and women's dispensing patterns? (this is described in the case of choosing to estimate dispenses with more than one ASM 'as a potential proxy for severity').<br>• The description of methods could be clarified in a number of ways. 1) Data used should be described before analytical strategies (eg first rows of Method section states trends computed and analysed, this should be left for a later section). 2) all analytical choices should be clearly linked to the paper's scientific objectives as spelled out in the last paragraph of the Introduction (eg p. 4 line states inclusion of men 'for comparison' but it is unclear what specifically is being compared and why – are they used as a benchmark for time trends since policy didn't explicitly recommend different prescribing for men of reproductive age (are they used as a type of negative control, to check what other temporal trends are affecting SV and ASM prescribing/dispensing)? 3) Analysis section about distribution of SV comparisons could be shortened and details moved to supplementary materials (it is confusing to read with all the details about geographical groupings and comparisons).<br>• Could the authors have used hospitalisations including emergency admissions for estimating the morbidity burden of seizures, alongside mortality data for epilepsy?<br>• Given that epilepsy specific deaths are rare, the linear regression might not be the best modelling choice for time trends (data are likely to be over-dispersed). How did the authors account for this, and what sensitivity analyses or alternative modelling approaches could be used to check that the trends they identify are robust to the choice of linear regression? This is particularly important since this analysis is aimed at drawing inferences about the impact of a) policy change in recommending SV prescribing practices and b) pandemic -related healthcare changes.<br>• The Discussion is very thorough but will benefit from some editing as it is difficult to read in places and too long overall. In particular, it would be helpful to the reader if all of the commentary about clinical implications and impact of the results and recommendations for follow-up analyses were lifted and moved to a section dedicated to this, right before the final Conclusion paragraph. I also suggest that the authors streamline the writing throughout.<br>• The mortality analyses estimating effectiveness and implementation of the guidelines change and of the pandemic should be given more space in the Discussion (ideally one paragraph dedicated to this important part of the results). I recommend adding this when discussing results in the context of the

	current evidence (how have mortality rates responded to similar guideline changes elsewhere?), following more careful modelling approaches to produce more robust results (see previous comment).<br><br>Minor/Specific Comments<br>Abstract<br>• the Objectives and Methods paragraph should reflect the focus on pregnancy<br>• Methods section should include data source and type (linked electronic health records, pharmaceuticals dispensing etc), and clearly state how the mortality time-trends analysis relate to the focus of the manuscript (assessing the impact of the pandemic and guideline implementation on ASM prescribing).<br>• Results should include a brief comment on stratification by geographical area, deprivation and ethnicity.<br>• Throughout – please be consistent with use of present or past tense, also throughout the manuscript (eg in the abstract lines 23-25)<br>• Please define abbreviations upon their first use eg Abstract line 29 (CBP?).<br>Introduction<br>• Since SV's known teratogenic effect, the authors could use a causal language and refer to effects rather than associations (eg p. 4, line 7).<br>Results<br>• Figures 4 and 5 should be in colour or better differentiated with shades of grey (difficult to distinguish between categories).<br>• Footnote to Figure 6 is better placed in a table, or as text.<br>• P. 8 line 31 – the authors should avoid reference to the sign of the slope here, which is very close to zero in terms of point estimate and with extremely wide confidence intervals as to be compatible with both a large positive or large negative direction of association.

**REVIEWER NAME**	Perera, Rafael
**REVIEWER AFFILIATION**	University of Oxford, Primary Care Health Sciences
**REVIEWER CONFLICT OF INTEREST**	None
**DATE REVIEW RETURNED**	07-Feb-2024

**GENERAL COMMENTS**	Statistical Review<br>The present study uses data from individuals in anti-seizure medication to evaluate trends associated with changes in policy/advice regarding sodium valproate (SV) safety. The period explored was from 1st January 2019 to 31st December 2021, with data available for England and Wales (~60 million). As part of a secondary analysis, English data on mortality were analysed from a separate data source (ONS) from January 2015 to December 2022. Their principal findings are a continuous reduction of SV in women (2019 to 2022) but not in men, while the volume of other anti-seizure medications increased or stayed stable over that period.<br>The main findings appear correct, and the information presented is likely reassuring in that the guidance for reducing SV in women of childbearing potential (CBP) is having a steady impact. Of interest is that this reduction has not plateaued, even if the initial recommendation was given in 2013. Part of the challenge in this paper is the lack of clarity of the research question and too many analyses partially obscuring the findings. Some specific comments aiming to improve the manuscript are given below.<br>1. The 'Research in Context' section is too long and descriptive. This section usually benefits from having two to three bullet points for each section: what is known, what this adds, and how this study impacts. I suggest that converting this section to a few bullet points will help clarify the rest of the paper and determine if all the analyses

	need to be included.<br><br>2. The current analysis presents information from (substantially) fewer than 60 million individuals, and the current explanation is slightly misleading. Please provide a clear flowchart of how the individuals are filtered from the original 60 million until the number used for each of the analyses. As the data could not be combined for England and Wales, I suggest a separate flowchart for each nation.<br><br>3. One of the most challenging parts of interpreting the analyses is that the current background expands on recommendations only for women of childbearing potential (CBP). Also, these recommendations were made in 2013 and strengthened in 2018, both time points outside the period used for the primary analysis. It is, therefore, unclear what the main question is being asked as no evidence for the impact of these recommendations could have been estimated given the data. The inclusion of men in the analysis could be made as a comparator group, but only if a clear justification for this being an adequate comparator.<br><br>4. Regarding the point of a comparator group and the type of analyses carried out (linear regression and time series analyses), an interrupted time series approach would have been more appropriate if the interest is in evaluating the impact of the change in guidance (recommendations). Please see {doi: 10.1136/bmj.h2750} as a brief introduction to these methods.<br><br>5. Information in the Background about the advice (across the period studied) for the other ASMs would be particularly relevant to interpreting these findings. Has the advice to reduce the use of SV been accompanied by suggesting instead a particular alternative?<br><br>6. Please provide some information in the Methods or the Discussion about the level of ascertainment of the volume for the different ASMs. Is this equal, or are there variations? Particularly important if these are different over the period studied.<br><br>7. There is no clarity about what data are being used for the analyses. Pay particular attention to linking each result to the data being included. For example, in the Abstract, England and Wales provide the totality of the data (~60M). The Methods describe that data from the two nations could not be combined due to important differences, so it needs to be made clear that the Results in the Abstract (and elsewhere) represent a specific nation (England?). The same issue is present in the rest of the manuscript.<br><br>8. The rationale for the pregnancy prediction model is unclear. This is, at most, an association model exploring factors linked to pregnancy. Describing it as a prediction model is not helpful as prediction models require a higher level of development (and validation) to what is being presented (and used for). Please adjust the terminology and expand on the rationale.<br><br>9. Minor technical point: for ARIMA models to be used, stationarity is needed. What methods were used to test or convert the time series? Also, what is the rationale for using these models, and what was the question being addressed? A change in the process? When defined?<br><br>10. The uptick after the national lockdown is also present post-pandemic (and to a lesser extent during). This might be a seasonal effect (challenges of using ARIMA).<br><br>11. Figure 2 - These look like general trends, and how long they have been going on is unclear. Since 2018? 2013? Also, clarification

	is needed if these are incident or prevalent cases.<br><br>12. Figure 5 – Please provide the total numbers (not only rates. From this analysis, there appears to be a reduction across most of the ASMs, not only SV. This might require a comment in your Discussion.<br><br>13. Figure 6 - Why is the x scale not constant? Also, expecting an immediate discontinuity would need to be justified. What is the rationale for the expected change if it was only a strengthening of the recommendations? Would a difference in difference approach provide more robust evidence (if this methodology is valid)?<br><br>14. Table 1 – Please also provide absolute numbers for these (as well as rates).

VERSION 1 – AUTHOR RESPONSE

Reviewer: 1
Dr. Andrea Schaffer, University of Oxford Nuffield Department of Primary Care Health Sciences

	Reviewer Comment	Author Response
1.0	This manuscript describes patterns valproate and other antiepileptic dispensing in England and Wales, with a focus on people of child-bearing age. There are a lot of interesting data here, however when reading it I found it a bit difficult to navigate as there are so many different results and analyses. One of the problems with trying to include too much is that you can't do justice to everything - some of the analyses are only given a few short sentences in the Methods and the Results when they warrant more. I suggest the authors consider paring it down (potentially addressing some aspects in a separate paper), such as focussing on the prescribing patterns analyses only - I think this would make it a much stronger paper	We thank the reviewer for their comments and have suggested changes to pare the manuscript down in line with their advice to make the message of the manuscript stronger and clearer.<br><br>To make the navigation of the manuscript easier we have outlined clear aims in the introduction as well as shortening this section. The structure of the methods and reporting of the results follow these aims.<br><br>As in our response to the Editor, we also think that there are a number of questions which could be picked up in subsequent analyses and we thank the reviewer for the advice in helping us to tighten up the message of our manuscript.<br><br>As suggested, we have refocused the manuscript on the dispensing patterns using men and other ASMs as comparators and removed several additional analyses from the manuscript, particularly those that were presented in the supplementary section.<br><br>In summary, we have removed:<br>• Stratifications by ethnicity, geographical region and IMD<br>• Logisitic regression of factors associated with sodium valproate pregnancy<br>• Secondary care analyses<br><br>And we have revised:<br>• Monthly trends of all ASMs by gender<br>• Flow diagram of data included<br>• Population pyramids of prevalent and incident dispenses

		• Epilepsy-related mortality models<br><br>In addition, other figures have been updated to include the most recently available data. Corresponding changes to the methods and results are tracked in the manuscript.
1.1	To help readers navigate this work, this manuscript would benefit from a clear and explicit statement of its aims, including each specific outcome being addressed. Currently, the stated aims are "to understand the use of valproate in women of CBP, and the impact of regulatory changes, including whether the COVID-19 pandemic impacted on the implementation of regulatory advice" But this is a bit too vague – it wasn't until I got to the Methods that I learned that the authors were also going to be predicting pregnancy, and looking at changes in seizure mortality.	Thank you for this helpful comment. We agree that this change would make the manuscript easier to navigate. In line with the reviewer's suggestion we have added an aims section and linked the structure of the methods and results more clearly to these.<br><br>The revised aims are:<br><br>(1) To describe the use of sodium valproate across the population of England and Wales during the period 2019-2023, following MHRA policy changes (April 2018), the Covid-19 pandemic (March 2020), the publication of the Cumberlege report (July 2020) and the Government's response to it (2021)<br><br>(2) To put the use of sodium valproate in the context of other ASMs (lamotrigine, levetiracetam, carbamazepine, topiramate), and to benchmark use in women against men in whom no policy change was implemented during the study period<br><br>(3) To describe geographical variation in the rate of use of sodium valproate by women of CBP (age 15-49) by local authority district, which is important for implementation of regulatory policy<br><br>(4) To describe the use of sodium valproate amongst women of CBP, including associated disease indications, and during pregnancy<br><br>(5) To explore population wide methods to aid understanding of the wider impact of policy changes, using epilepsy-related mortality as an example<br><br>We have revised the introduction and adjusted the methods, results and discussion, and introduced sub-headings in the manuscript in line with this delineation of the aims.
1.2	I'm curious why the authors chose to compare counts by age and sex (Figure 3) instead of rates? For instance, the authors state: "Distribution of dispense of SV…was much lower in women	We thank the reviewer for this important observation. One of the reasons that drove us to focus on counts in the first instance is to inform the translation of these findings into clinical care. Whilst a rate is a useful metric for comparing and reporting between groups, to implement at a local level knowing how many women of childbearing potential are being dispensed sodium valproate and where they are

	of CBP compared to men in the same age bands." But that may be partially explained by differences in the population at risk - wouldn't a comparison of rates be more appropriate? Differences by sex/age may also be explained by differences in rates of the conditions that these medicines treat. Perhaps a better way to look at this this would be to examine the distribution of ASMs among people dispensed any ASM only.	provides a mechanism to those implementing policy to measure how effective their implementation is. This was informed by our interactions with those that need to implement policy, who have advised that these numbers, are the most useful metric to track implementation.<br><br>Another reason to focus on absolute counts was the very low and declining numbers in women. The low counts relative to the population at risk will therefore result in very small and relatively stable rates, but these may mask important instances where policy has not been implemented.<br><br>However, we do agree with the reviewer that representing the data as rates to compare use independent of population structure in different populations at risk can be a very useful. We have already implemented this in revised Figure 3 which shows the rates amongst women of childbearing potential geographically. We also present rates of indications in revised Figure 4 and rates of pregnancy by ASM in revised Figure 5.<br><br>We feel that looking rates among people dispensed any ASM could potentially be misleading here as, picking up on the editor's comment E3, there is likely to have been some temporal underdiagnosis of epilepsy during the study period due to pandemic restrictions and therefore the denominator may not be stable, hindering interpretation of these rates. We also refer the reviewer to the comment and response to the editors comment in E8, where diagnoses may be uncertain given that prescriptions are not routinely recorded against a diagnosis.<br><br>As an alternative, we present comparative data from:<br>(1) men – where no policy changes were implemented and<br>(2) other ASMs to better understand the changes in sodium valproate dispense during the study period.<br><br>We have therefore revised Figure 2a and Supplementary Figures 1a-1c bringing them into the main paper and moving Figure 3 to Supplementary Material (now Supplementary Figure 1).
1.3	I have a couple of issues with the logistic regression predicting pregnancy. First, the goal of this analysis is not clear, and how it fits in with the stated aims. A clearer statement of the rationale is needed to understand whether the methods used and interpretation of results are appropriate. For instance, the	Thank you for this constructive comment. We have reflected on the inclusion of the logistic regression in the manuscript. Our motivation for this analysis was to help inform how a range of variables may associate with use of valproate during pregnancy, rather than a formal prediction analysis.<br><br>However, we agree with the reviewer that there are already a lot of data within this manuscript and that the focus should be on the patterns

	authors state they wanted to “predict” pregnancy, but I suspect the goal was to identify associations, as the approach to model building for these two aims differ (this is covered by: Laubach et al. A biologist’s guide to model selection and causal inference. Proc Biol Sci 2021 10.1098/rspb.2020.2815.) Second, the description of this analysis is lacking in detail – over what time period were the predictors measured, in relation to assessment of the outcome (this is particularly important)? How were the potential “predictors” selected? How was the final model chosen? Given these issues, I don’t think this analysis contributes much, and the paper would be stronger without it.	in dispensing, and these analyses require attention that could be addressed in a follow up manuscript.<br><br>We have therefore removed the logistic regression from the main manuscript, but highlighted in the final paragraph of the discussion the need for further research to better understand the increasingly rare, but ongoing, instances of use of sodium valproate during pregnancy.
1.4	For the analysis of epilepsy-related deaths, the outcome is the number of deaths, which is quite a rare event. Therefore, the authors should consider whether a count model (Poisson or negative binomial) more appropriate. As with the logistic regression, I think this analysis is less interesting than the prescribing patterns analysis.	Thank you for this important observation. We also refer the reviewer to the comments made to the editor in E7. As stated in our final aim, we would like to begin to build an approach to help assess impact of unintended consequences of policy and here we use mortality as an example.<br><br>The MBRRACE report which has highlighted a potential increase in maternal mortality during pregnancy and the post-partum period due to Sudden Unexpected Death in Epilepsy (SUDEP), although absolute numbers were low hindering ability to draw definition conclusions (https://www.npeu.ox.ac.uk/assets/downloads/mbrrace-uk/reports/maternal-report-2020/MBRRACE-UK_Maternal_Report_Dec_2020_v10_ONLINE_VERSION_1404.pdf). Since SUDEP may be influenced by restrictions in prescribing of sodium valproate we feel it is a complementary analysis to the prescribing patterns.<br><br>In response to this comment and also Reviewer comment 2.6 we have conducted sensitivity analyses re-modelling the mortality data using Poisson regression. We find results from the Poisson models to be consistent with the linear models. Please see Alternative Figure 6 below including results from both models. Since results were consistent with both approaches we have retained the original linear models in the manuscript; however these could be changed to the Poisson models should the reviewer and editor prefer.<br><br>While updating these analyses we were able to refresh the mortality data which are now more complete from the data provider. Analysing these updated data we observe some evidence for a slight increase in epilepsy-related mortality in men aged 15-49 with both the linear and Poisson models. We have updated results accordingly and added some commentary on this finding to the discussion.

		These are important analyses in light of the MHRAs position on restricting use of sodium valproate in all those aged under 55, including men, as outlined in addressing the Editors comment E7.
1.5	Data on valproate dose is presented in the Results, but I didn't see it mentioned in the Methods, or in the Supplementary Methods either. How was dose ascertained? How complete is that data?	Thank you for this comment and apologies for the omission of the description. The method for calculating dose has been added to the methods section on page 8.<br><br>Within the NHSBSA data each dispensing event is accompanied by information about the quantity (number of items e.g. tablets) and medicine strength (concentration either in mg or mg/ml). Dose was calculated by multiplying the quantity of items at each event by the concentration after converting this to grams. The total number of grams dispensed was summed over the total pregnancy episode and during each trimester. This was divided by the corresponding number of days to calculate average grams per day (280 for the whole pregnancy episode; 90 for the first trimester).
1.6	Some of the acronyms don't seem necessary (like SV).	We have replaced SV with sodium valproate throughout the manuscript. We have removed the acronym TRE as it was not referred to later in the manuscript. We can replace further acronyms if required.
1.7	How was the list of ASMs included in the study selected? What about other antiepileptics like gabapentinoids or clonazapem?	The preliminary list of ASMs was drawn from the British National Formulary CNS sub chapter on anti-seizure medications. This was downloaded and curated to cluster drugs with similar mechanism of action, e.g. levitaracetam and brivaracetam were analysed together.<br><br>The preliminary list included gabapentinoids and benzodiazepenes; however, these were not initially selected in the group of ASMs, as in the case of gabapentinoids, these are not only used for epilepsy; and benzodiazepines are often only used for emergency treatment or short-term control and bridging (e.g. clobazam).<br><br>The focus of this manuscript was on sodium valproate with other potential ASMs used to control epilepsy, particularly in women of child-bearing potential, included as comparators, hence the focus on the five selected.<br><br>We have also analysed the broader group – please see Alternative Figures 2a & 2b shown below. However, these figures are not included in the revised manuscript version as we feel it obscures the trends in sodium valproate which are the main subject of the manuscript; but if the reviewer feels that it is useful to add to the supplementary figures we can do so.

1.8	The authors use the term “dispenses” as a noun. In my experience working with medicine data, I’ve not heard that term before – I’m more familiar with “dispensings” (or less commonly, “dispensations”). A quick search of the literature confirms that “dispenses” is uncommon.	Thank-you for raising this. We have changed dispenses to dispensings throughout the manuscript.
1.9	In Figure 4, the y-axis says Count, but the title says rate.	Thank-you for highlighting this error. The y axis label has been changed to Rate.

Reviewer: 2
Dr. Luisa Zuccolo, Fondazione Human Technopole, University of Bristol
Comments to the Author
Reviewer: Dr. Luisa Zuccolo

This study describes time trends in the use of sodium valproate, a known teratogen, and other anti-seizure medications in the English and Welsh general population, by demographic characteristics, and with a focus on women of childbearing age and pregnant women in particular. The authors also estimate whether a prescribing guideline change (in 2018) and more general changes in healthcare systems and access during the COVID-19 pandemic (2020 -22) affected anti-epileptic drugs dispensing and/or rates of deaths due to epilepsy. Linked electronic health records covering the entire national populations of England and Wales, many aspects of healthcare utilization, and an 8 year period provide detailed phenotyping and statistical power to the analyses.

I congratulate the authors on a well powered, well designed and generally well presented piece of research. The scale of the population coverage and clinical importance of the medication(s) make this a high impact study. I have some general comments, mainly pertaining to the way that objectives, methods and results are organized, a request to add sensitivity analyses to the mortality trends component and a suggestion to streamline the Discussion. Below the authors can find my general and more specific comments. I have recommended that the paper be accepted following minor revisions.

	**Reviewer Comment**	**Author Response**
	**General Comments**	
2.0	I congratulate the authors on a well powered, well designed and generally well presented piece of research. The scale of the population coverage and clinical importance of the medication(s) make this a high impact study. I have some general comments, mainly pertaining to the way that objectives, methods and results are organized, a request to add sensitivity analyses to the mortality trends component and a suggestion to streamline the Discussion. Below the authors can find my general and more specific comments. I have recommended that the paper be accepted following minor revisions.	We thank the reviewer for her very kind remarks and have addressed her more specific points below.
2.1	The Research in context section on page 3 could be shortened considerably, and it is not advisable for this section to include so many acronyms.	This section has been shortened, also reducing the number of acronyms. We have also removed the acronyms SV and TRE.
2.2	The scientific objectives need to be made more explicit: could the authors also separate out descriptive objectives from the inferential analysis estimating the effects of guidelines change and pandemic? In particular, there needs to be more consistency between phrasing of research aims in the last paragraph of the Introduction and analytical strategies used, both throughout the Methods and Results chapters (subheadings relative to each	Thank-you for this point also highlighted by the editor and other reviewers. Please also see response to reviewer comment 1.1 where we clarify highlight the aims that have been added to the revised manuscript.<br><br>As suggested, we have also added sub-headings to the Methods and Results sections. These are:

	research objective would greatly help here) (eg currently there is no mention of investigating pregnancies).	• Prevalent and incident dispensings of sodium valproate and other ASMs over time and variation by sex<br>• Geographical variation in dispensings of sodium valproate<br>• Disease indications in women of CBP dispensed sodium valproate<br>• Pregnancy rates in women of CBP dispensed sodium valproate and other ASMs<br>• Trends in epilepsy & recurrent seizures mortality by sex
2.3	A clearer link between research objectives and analyses could help understand analytical choices, eg why were men included? What can we learn from the comparison between men and women's dispensing patterns? (this is described in the case of choosing to estimate dispenses with more than one ASM 'as a potential proxy for severity').	Please see response to editor (E7) and comments made by reviewer 1 (1.1), where we provide an explanation of why we have included men, and also now also other anti-seizure medications, as comparators to act as benchmarks against which sodium valproate can be compared. We hope these responses already address this comment in full, but please do let us know if you would like further clarification.
2.4	The description of methods could be clarified in a number of ways. 1) Data used should be described before analytical strategies (eg first rows of Method section states trends computed and analysed, this should be left for a later section). 2) all analytical choices should be clearly linked to the paper's scientific objectives as spelled out in the last paragraph of the Introduction (eg p. 4 line states inclusion of men 'for comparison' but it is unclear what specifically is being compared and why – are they used as a benchmark for time trends since policy didn't explicitly recommend different prescribing for men of reproductive age (are they used as a type of negative control, to check what other temporal trends are affecting SV and ASM prescribing/dispensing)? 3) Analysis section about distribution of SV comparisons could be shortened and details moved to supplementary materials (it is confusing to read with all the details about geographical groupings and comparisons).	We have restructured the methods as indicated, adding sub -headings in line with the revised aims highlighted in response to Reviewer 1 comment 1.1<br><br>We hope the reason for the inclusion of men as a comparator group is now clearer in the revised manuscript – please see responses to E7 and reviewer comment 1.2, and the response above.<br><br>We have moved original Figures 3a and 3b (population pyramids) to the supplementary section as suggested.
2.5	Could the authors have used hospitalisations including emergency admissions for estimating the morbidity burden of seizures, alongside mortality data for epilepsy?	Yes, we have looked at this previously and include figures on epilepsy-related hospital admissions – please see figures below.<br><br>However, trends in admissions appear characteristic of the Covid-19 pandemic in general and therefore we prefer not to add these analyses to the manuscript at this stage given

		overall editor and reviewer comments regarding streamlining content.
2.6	Given that epilepsy specific deaths are rare, the linear regression might not be the best modelling choice for time trends (data are likely to be over-dispersed). How did the authors account for this, and what sensitivity analyses or alternative modelling approaches could be used to check that the trends they identify are robust to the choice of linear regression? This is particularly important since this analysis is aimed at drawing inferences about the impact of a) policy change in recommending SV prescribing practices and b) pandemic-related healthcare changes.	As per response to Reviewer 1, comment 1.4 – we have undertaken sensitivity analyses re-modelling this data with Poisson regression, finding consistent results with the linear models (please see Alternative Figure 6 below).
2.7	The Discussion is very thorough but will benefit from some editing as it is difficult to read in places and too long overall. In particular, it would be helpful to the reader if all of the commentary about clinical implications and impact of the results and recommendations for follow-up analyses were lifted and moved to a section dedicated to this, right before the final Conclusion paragraph. I also suggest that the authors streamline the writing throughout.	Thank-you for this comment. The discussion has been revised and shortened. The tracked changes attachment outline where and how these edits have been made. We hope that this more focussed approach meets with the Reviewer's concerns.
2.8	The mortality analyses estimating effectiveness and implementation of the guidelines change and of the pandemic should be given more space in the Discussion (ideally one paragraph dedicated to this important part of the results). I recommend adding this when discussing results in the context of the current evidence (how have mortality rates responded to similar guideline changes elsewhere?), following more careful modelling approaches to produce more robust results (see previous comment).	Thank- you for this suggestion. Please see paragraphs 7 and 8 of the revised discussion.
	**Minor/Specific Comments**	
	Abstract<br><br>the Objectives and Methods paragraph should reflect the focus on pregnancy	This has been revised.

	Methods section should include data source and type (linked electronic health records, pharmaceuticals dispensing etc), and clearly state how the mortality time-trends analysis relate to the focus of the manuscript (assessing the impact of the pandemic and guideline implementation on ASM prescribing).	We have added this to the methods and revised the flowchart (Figure 1) to improve clarity.
	Results should include a brief comment on stratification by geographical area, deprivation and ethnicity	In response to reviewer and editor's comments to improve the clarity of the manuscript, these analyses have been removed from the manuscript.
	Throughout – please be consistent with use of present or past tense, also throughout the manuscript (eg in the abstract lines 23-25)	We have used the past tense throughout the abstract.
	Please define abbreviations upon their first use eg Abstract line 29 (CBP?).	Thank-you, and apologies for the omission on the first reference of the acronym (CBP = Child Bearing Potential). This has now been updated and included this definition earlier on first use in the abstract.
	Introduction<br>Since SV's known teratogenic effect, the authors could use a causal language and refer to effects rather than associations (eg p. 4, line 7).	We have avoided using causal language in this manuscript, as whilst the teratogenic effects are known to be causal, we are in this manuscript describing a narrative of the use.<br><br>Also, while there is a known teratogenic effect of sodium valproate, much remains unknown, for example about the role of dose and timing.
	Results<br>Figures 4 and 5 should be in colour or better differentiated with shades of grey (difficult to distinguish between categories).	Thank-you, to confirm these figures are in colour – please see manuscript. We therefore assume the reviewer was sent a black and white version of the manuscript. If this is correct, we are able to change to different colours with better differentiation at the editor's request.
	Footnote to Figure 6 is better placed in a table, or as text.	Thank you, we have replaced this with a table as suggested.
	P. 8 line 31 – the authors should avoid reference to the sign of the slope here, which is very close to zero in terms of point estimate and with extremely wide confidence intervals as to be compatible with both a large positive or large negative direction of association.	Thank you, we have revised the language we use to describe these results

Reviewer: 3
Prof. Rafael Perera, University of Oxford
Comments to the Author
bmjmed-2023-000760 Use of sodium valproate and other anti-seizure medications in England and Wales during the COVID-19 pandemic: a population-scale analysis of 60 million individuals
Statistical Review
The present study uses data from individuals in anti-seizure medication to evaluate trends associated with changes in policy/advice regarding sodium valproate (SV) safety. The period explored was from 1st January 2019 to 31st December 2021, with data available for England and Wales (~60 million). As part of a secondary analysis, English data on mortality were analysed from a separate data source (ONS) from January 2015 to December 2022. Their principal findings are a continuous reduction of SV in women (2019 to 2022) but not in men, while the volume of other anti-seizure medications increased or stayed stable over that period.
The main findings appear correct, and the information presented is likely reassuring in that the guidance for reducing SV in women of childbearing potential (CBP) is having a steady impact. Of interest is that this reduction has not plateaued, even if the initial recommendation was given in 2013. Part of the challenge in this paper is the lack of clarity of the research question and too many analyses partially obscuring the findings. Some specific comments aiming to improve the manuscript are given below.

	**Reviewer Comment**	**Author Response**
3.1	The 'Research in Context' section is too long and descriptive. This section usually benefits from having two to three bullet points for each section: what is known, what this adds, and how this study impacts. I suggest that converting this section to a few bullet points will help clarify the rest of the paper and determine if all the analyses need to be included.	Thank-you for this suggestion. The 'Research in Context' has now been shortened and changed to bullet points.<br><br>This was also raised by Reviewer 2 (comment 2.1).
3.2	The current analysis presents information from (substantially) fewer than 60 million individuals, and the current explanation is slightly misleading. Please provide a clear flowchart of how the individuals are filtered from the original 60 million until the number used for each of the analyses. As the data could not be combined for England and Wales, I suggest a separate flowchart for each nation.	Thank you, please see response to editor comment E5. We have removed 60 million individuals from the discussion and the suggested revised title (see comment E1).<br><br>Flowcharts have been updated and presented separately for England (Figure 1) and Wales (Supplementary Figure 1) as suggested. These document data linkage and the number of medicines and individuals included.
3.3	One of the most challenging parts of interpreting the analyses is that the current background expands on recommendations only for women of childbearing potential (CBP). Also, these recommendations were made in 2013 and strengthened in 2018, both time points outside the period used for the primary analysis. It is, therefore, unclear what the main question is being asked as no evidence for the impact of these recommendations could have been estimated given the data. The inclusion of men in the analysis could be made as a	Thank-you for this insightful comment – please see also the response to Editor comment E1.<br><br>We agree that we are limited by the lack of availability of dispensing data prior to 2018. However, we can demonstrate the real ongoing and cumulative impact of the various contributing factors influencing use of sodium valproate from 2019 to the end of 2023 (MHRA policy changes, publication of the Cumberlege Report and government response, Covid-19 pandemic). We reference a study from Scotland (McTaggart S et al., 2018 doi.org/10.1136/bmjopen-2021-058312) which presents

	comparator group, but only if a clear justification for this being an adequate comparator.	trends prior to our study period (2011-2019). Given that policy changes are UK wide, trends in England & Wales are likely to be similar. The question that we are seeking to address, is whether restrictions on use of sodium valproate were hampered by Covid-19. While it is not possible to judge whether declines would have happened at a faster rate without the pandemic, importantly we are able to report that declines were sustained. However, we do agree that a more comprehensive analysis would be possible with historic data.<br><br>The additional reason for also carrying out these analyses, as highlighted to the editor, is to make the point as to how data science can be used to track changes using whole population data. Whilst smaller datasets like CPRD can estimate changes, actual counts are beneficial to action implementation as well as track the effects of regulatory advice. As such our data has already been used by the Patient Safety Commissioner in her Redress report (https://www.patientsafetycommissioner.org.uk/our-reports/the-hughes-report/), and in discussion with her and others we are now working towards how to action the analytics described in this manuscript.<br><br>In the revised manuscript we have clarified the inclusion of men and improved the justification of their use as comparator group – please see responses to editor E7 and reviewer comment 1.2. We also include the other ASMs as comparator groups during the study period, since these were not subject to policy changes. We highlight this in the revised manuscript including updated and extended Figures 2a and 2b. Tracking men will also now be more important in light of the most recent MHRA's advice as well as our revised analyses (Figure 6) which suggest epilepsy-related mortality may be increasing amongst men. This is important to monitor ongoing, and this manuscript can help set the scene for this to be done.
3.4	Regarding the point of a comparator group and the type of analyses carried out (linear regression and time series analyses), an interrupted time series approach would have been more appropriate if the interest is in evaluating the impact of the change in guidance (recommendations). Please see {doi: 10.1136/bmj.h2750} as a brief introduction to these methods.	Thank-you for this suggestion. Please also see responses to reviewer comments 1.4 and 2.6 where we discuss improvements and sensitivity analyses we have undertaken around the modelling of epilepsy-related mortality.<br><br>We have experience of using interrupted time-series analysis with the NHSBSA data from a previous publication ((Dale & Takhar, 2023 doi.org/10.1038/s41591-022-02158-7). However, as the reviewer correctly highlights in comment 3.3, the limitation of this data for the current research question is the relatively short period of time pre-pandemic; and in particular, the absence of any data prior to the key MHRA policy changes in 2018 which would be the most relevant "interruption". For the epilepsy-related

		mortality analyses (Figure 6) we are able to model before and after this key policy change.<br><br>In addition, several of the potential contributing factors overlap in time (MHRA policy changes, Covid-19 pandemic, publication of the Cumberlege report and Government response) making it impossible to delineate clear time periods for each. Given these limitations, we feel it is more appropriate to present a clear description of the trends observed rather than attempt to imperfectly attribute particular periods of change to specific causes.
3.5	Information in the Background about the advice (across the period studied) for the other ASMs would be particularly relevant to interpreting these findings. Has the advice to reduce the use of SV been accompanied by suggesting instead a particular alternative?	Thank you for this suggestion. The advice on the MHRA website is to consider alternative ASMs including lamotrigine which are not associated with the teratogenic effects of sodium valproate. As the reviewer will be able to see in revised Figure 2b we have now updated to include new starters (initiators) on each ASM, which addresses new starters on sodium valproate relative to other ASMs, and in particular those that are recommended.<br><br>In addition, during this study, some data for the teratogenic effect for topiramate has begun to emerge, although this is not universally established. This follows recent observational evidence that prenatal exposure to topiramate is associated with an increased risk of autism spectrum disorders, intellectual disability, and neurodevelopmental disorders (Bjork MH et al. JAMA Neurol 2022;79(7):672-681. DOI: 10.1001/jamaneurol.2022.1269.). However, unlike valproate topiramate does not currently have a formal pregnancy prevention program in place, and risks are thought to be of a lower magnitude than valproate although evidence is still emerging. The MHRA have started a new safety review to assess the benefits and risks of topiramate and to consider whether further measures are required to reduce the risk of harm associated with topiramate use during pregnancy ().<br><br>Our incidence charts also demonstrate that topiramate is the ASM that most women are initiating (Figure 2b & Supplementary Figure 1). Results also indicate that even in the absence of a PPP, the rate of dispense of topiramate during pregnancy is lower than other ASMs (Figure 5), although not as low as valproate, suggesting there may be further potential for reduction. Furthermore, there is some evidence that the rate of pregnancy amongst CBP women dispensed topiramate may be increasing.

		These finding demonstrate the utility of our approach in providing a mechanism to track alternatives as well as monitoring implementation of valproate policy.<br><br>Topiramate was previously highlighted in the background. We have further highlighted topiramate findings in the results and discussion in response to the reviewer's comment.
3.6	Please provide some information in the Methods or the Discussion about the level of ascertainment of the volume for the different ASMs. Is this equal, or are there variations? Particularly important if these are different over the period studied.	Thank you for this observation. The ascertainment of all medicines in primary care in the NHS SDE is through the NHSBSA data. This is the most comprehensive way of ascertaining all medicines that are dispensed through community pharmacies in the study population in England and Wales. There should not be any variation in ascertainment across the study period as these data have been primarily instituted for reimbursement, although through linkages are now also being used for monitoring. The NHSBSA data are the same data that are being used, for example by the NHS, to monitor trends e.g. EPACT 2 and Open Prescribing.
3.7	There is no clarity about what data are being used for the analyses. Pay particular attention to linking each result to the data being included. For example, in the Abstract, England and Wales provide the totality of the data (~60M). The Methods describe that data from the two nations could not be combined due to important differences, so it needs to be made clear that the Results in the Abstract (and elsewhere) represent a specific nation (England?). The same issue is present in the rest of the manuscript.	Thank-you for this important comment. As outlined in the response to the Editors comment E5 we have removed reference to 60 million individuals from the manuscript and clarified the data flow in Figure 1 and Supplementary Figure 1 as well as the narrative across the manuscript to it is clearer how many individuals, and from which nation, are included in each part of the analysis.
3.8	The rationale for the pregnancy prediction model is unclear. This is, at most, an association model exploring factors linked to pregnancy. Describing it as a prediction model is not helpful as prediction models require a higher level of development (and validation) to what is being presented (and used for). Please adjust the terminology and expand on the rationale.	Thank-you for this comment which is consistent with Reviewer Comment 1.3. In response to these comments, and also in response to the editor's and broader reviewer's comments to streamline and focus the manuscript, we have removed this model from the manuscript.
3.9	Minor technical point: for ARIMA models to be used, stationarity is needed. What methods were used to test or convert the	Thank-you for highlighting this. We can confirm that both male and female times series data satisfied the requirement for stationarity. This was confirmed using the Augmented

	time series? Also, what is the rationale for using these models, and what was the question being addressed? A change in the process? When defined?	Dickey–Fuller (ADF) and Kwiatkowski–Phillips–Schmidt–Shin (KPSS) tests. We have added this to the Methods.<br><br>The rationale of this analysis was to establish the level of epilepsy-related mortality prior to the MRHA policy change in March 2018 that restricted use of sodium valproate by women of child-bearing potential, and measure any subsequent change in the level of epilepsy-related mortality (April 2018 to December 2022). Given that sodium valproate is the evidence-based, first-choice, anti-seizure medication (ASM) for newly diagnosed idiopathic generalised epilepsy (IGE), it is possible that restriction of it's use may lead to unintended consequences including more uncontrolled seizures and deaths. The confidential enquiry investigating maternal deaths (MBRRACE-UK) report in 2020 (covering the period 2016-2018) highlighted an increase in maternal mortality during pregnancy and the post-partum period due to Sudden Unexpected Death in Epilepsy (SUDEP). As the Reviewer has already commented (3.3) ideally we would like a longer time period of data, however in the absence of these data and in the mounting evidence, it is also important to begin to build these analytical pipelines to be able to aid regulatory advice.
3.10	The uptick after the national lockdown is also present post-pandemic (and to a lesser extent during). This might be a seasonal effect (challenges of using ARIMA).	We agree with the reviewer that the upticks are difficult to reliably attribute. This sentence has therefore been removed from the results on page 10.
3.11	Figure 2 - These look like general trends, and how long they have been going on is unclear. Since 2018? 2013? Also, clarification is needed if these are incident or prevalent cases.	Thank you for this observation – we refer also to response 3.3 and response to the Editor comment E1.<br><br>Figure 2 represents prevalent use of sodium valproate benchmarking use in women against use in men and compared with other ASMs. Working within the NHS SDE we are limited to being able to access dispensing data from April 2018, with the first few months considered unstable (discussed in our CVD publication Nature Medicine, Dale & Takhar, 2023 doi.org/10.1038/s41591-022-02158-7). Hence we chose January 2019 as the start of the study period.<br><br>We have however as part of this response been able to update the data to the end of 2023 to include a greater time period, including both prevalent (Revised Figure 2a) and incident data (Revised Figure 2b). As in the response to the editor however, and pertinent to the comments above,

		having earlier data before the MHRA policy changes in 2018 would provide a much better baseline to begin to understand the nature of the effects of labelling and warnings on the use of valproate. We are, however, limited by the dispensing data availability in this respect, although we are able to include mortality data from 2015.<br><br>We have made the data included clearer in the revised flowcharts (Figure 1 and Supplementary Figure 1).
3.12	Figure 5 – Please provide the total numbers (not only rates. From this analysis, there appears to be a reduction across most of the ASMs, not only SV. This might require a comment in your Discussion.	As part of the revision we have been able to update the pregnancy rates by ASM for 2022. In the new data we observe a higher rate in 2021 for all ASMs and a lower rate for 2022. This is in line with trends in live births in England and Wales in 2022, which are reported to be 3.1% lower than in 2021 (https://www.ons.gov.uk/peoplepopulationandcommunity/birthsdeathsandmarriages/livebirths/bulletins/birthsummarytablesenglandandwales/2022). Results are also in line with the recent trend of decreasing live births observed before the coronavirus (COVID-19) pandemic. We are unable to include rates for 2023 as some pregnancies commenced in this year are still ongoing and not yet identifiable by HES delivery date.<br><br>We have added counts in addition to the rates to the table accompanying Figure 5 as suggested. Please also see the response to Reviewer 1, comment 1.2 where we discuss the utility of both absolute counts and rates in these analyses.<br><br>In these analyses we also observe a potential increase in the rate of pregnancy by women dispensed topiramate. Given the emerging evidence around the safety of topiramate this is an important finding that we have highlighted in the results and discussion. The inclusion of the counts helps highlight the size and relative importance of this group of women and the population level and we thank the reviewer for this suggestion.
3.13	Figure 6 - Why is the x scale not constant? Also, expecting an immediate discontinuity would need to be justified. What is the rationale for the expected change if it was only a strengthening of the recommendations? Would a difference in	Thank-you for this suggestion. The scale is now the same for both men and women (0 to 50 deaths per month) and we agree it improves the clarity of Figure 6.<br><br>While the 2018 MHRA policy change can be described as a strengthening of the recommendations, it also marked clear changes around the dispense of sodium valproate to

	difference approach provide more robust evidence (if this methodology is valid)?	women of child-bearing potential, including the requirement for the PPP to be in place. It can therefore be considered to be a turning point in relation to sodium valproate policy.<br><br>In response to comments from reviewers this analysis has been revised and improved (see reviewer comments 1.4 and 2.6). Revision of the analyses also gave an opportunity to refresh the data and the 2022 data is more complete. For this same reason however, we consider the 2023 mortality data currently too incomplete to include. In the updated analysis we now observe some evidence for an uplift in epilepsy-related mortality in men. We have updated the results and discussion accordingly.<br><br>We prefer not to implement a difference in difference approach to avoid complicating interpretation and because the parallel paths assumption may be violated due to gender-specific pandemic behaviours.
3.14	Table 1 – Please also provide absolute numbers for these (as well as rates).	Absolute counts of dispensings have now been added to table 1 in addition to rates per 100,000 and it has also been updated to 2022. It is not possible to include 2023 as ONS have not yet released mid-year population estimates for that year.

Alternative Figure 2a: Monthly counts of prevalent ASMs dispensed in the community January 2019 to December 2023; males and females, England. Vertical lines indicate dates of pandemic lockdowns for reference.

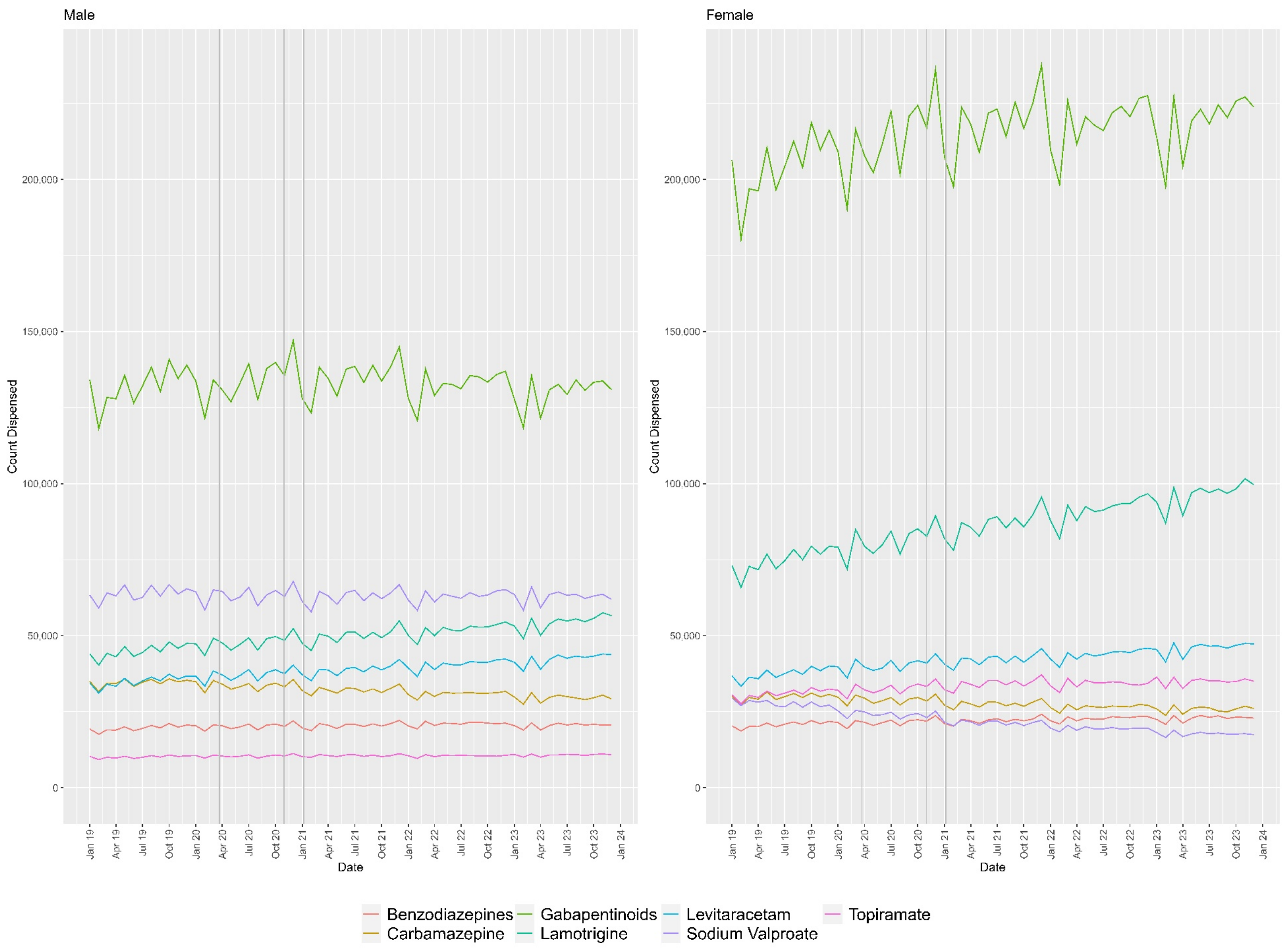

Alternative Figure 2b: Monthly counts of incident ASMs dispensed in the community January 2019 to December 2023; males and females, England. Vertical lines indicate dates of pandemic lockdowns for reference.

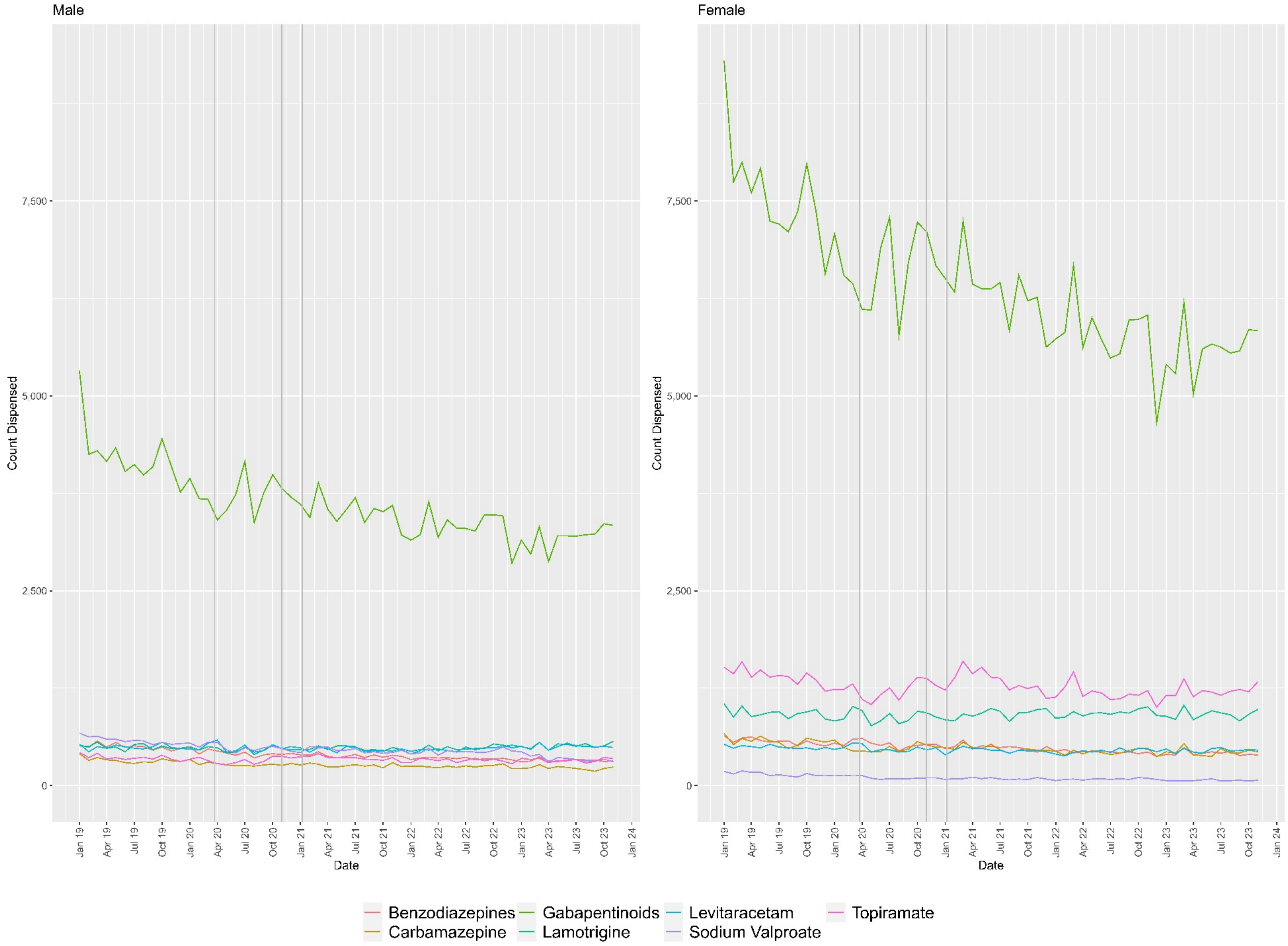

Alternative Figure 6: Epilepsy and recurrent seizures mortality (ICD-10 G40 & G41) over time; count of deaths in men and women of child-bearing age 15-49 years by year and month, 2015 to 2022; England. Solid black vertical lines indicate MHRA policy change; broken vertical lines indicate pandemic national lockdowns. Poisson and linear models.

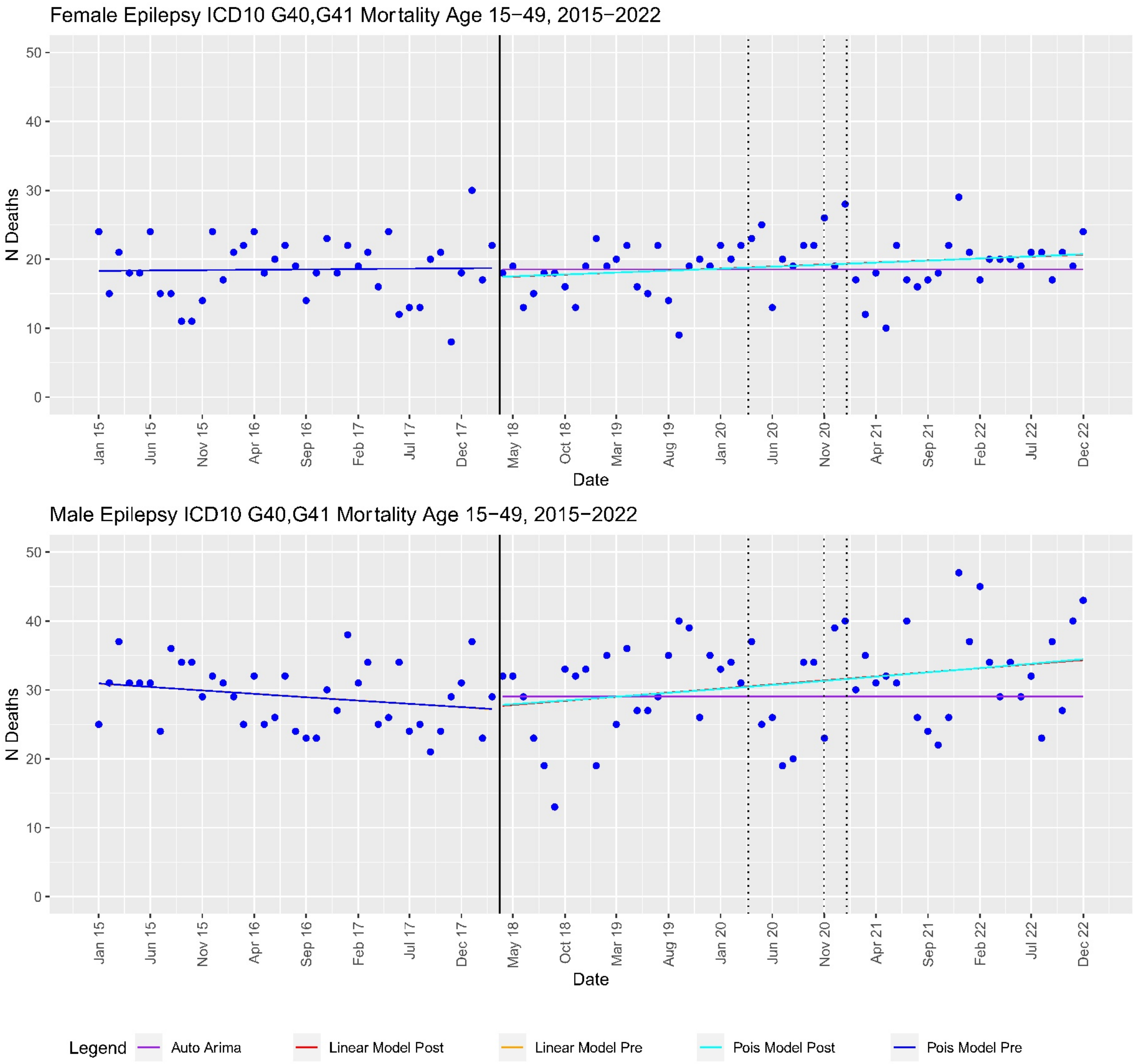

	Jan 2015 to March 2018			April 2018 to December 2022		
	Coefficient	SE	P-value	Coefficient	SE	P-value
Males						
Linear	-0.0032	0.0021	0.134	0.0039	0.0018	0.034
Poisson	-0.0001	0.0001	0.203	0.0001	0.0000	0.008
Females						
Linear	0.0004	0.0022	0.872	0.0019	0.0010	0.066
Poisson	0.0000	0.0001	0.861	0.0001	0.0001	0.098

** Poisson coefficient is exponentiated*

Hospital episode statistics (ICD-10 G40 & G41) over time; count of episodes by month

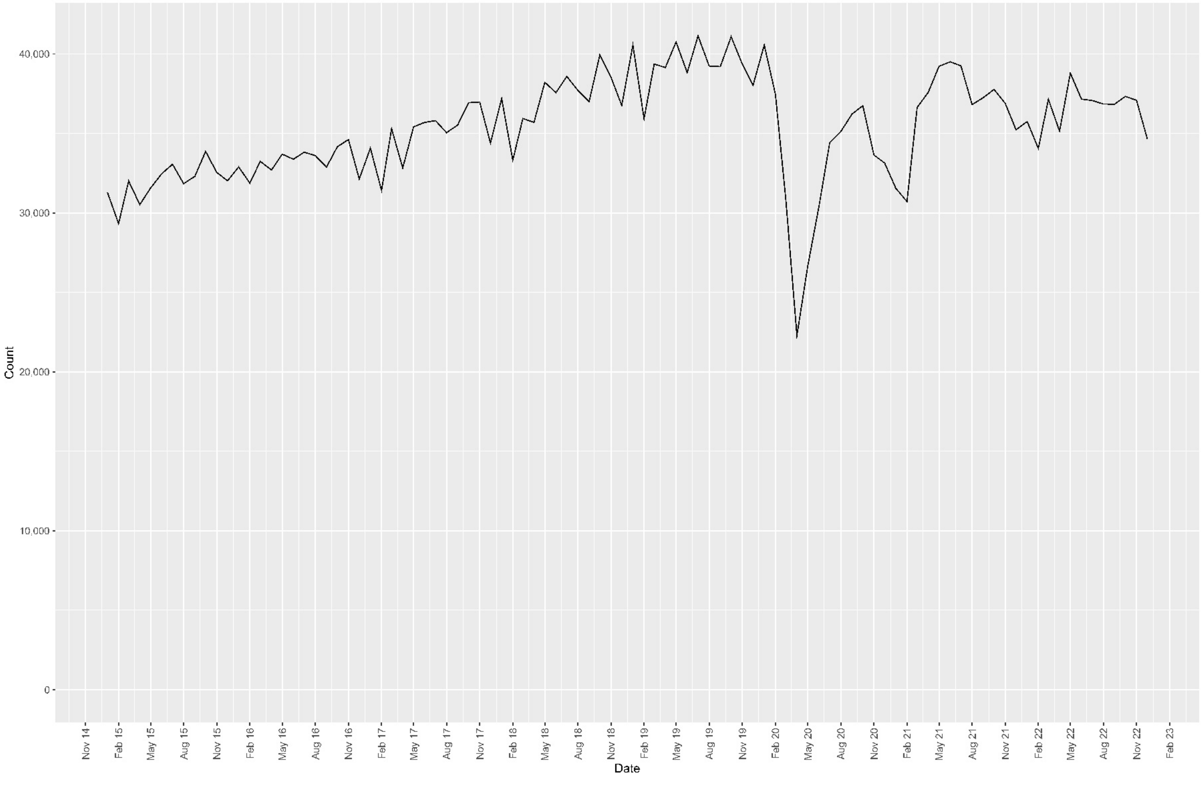

Hospital episode statistics (ICD-10 G40 & G41) over time; count of unique individuals by month

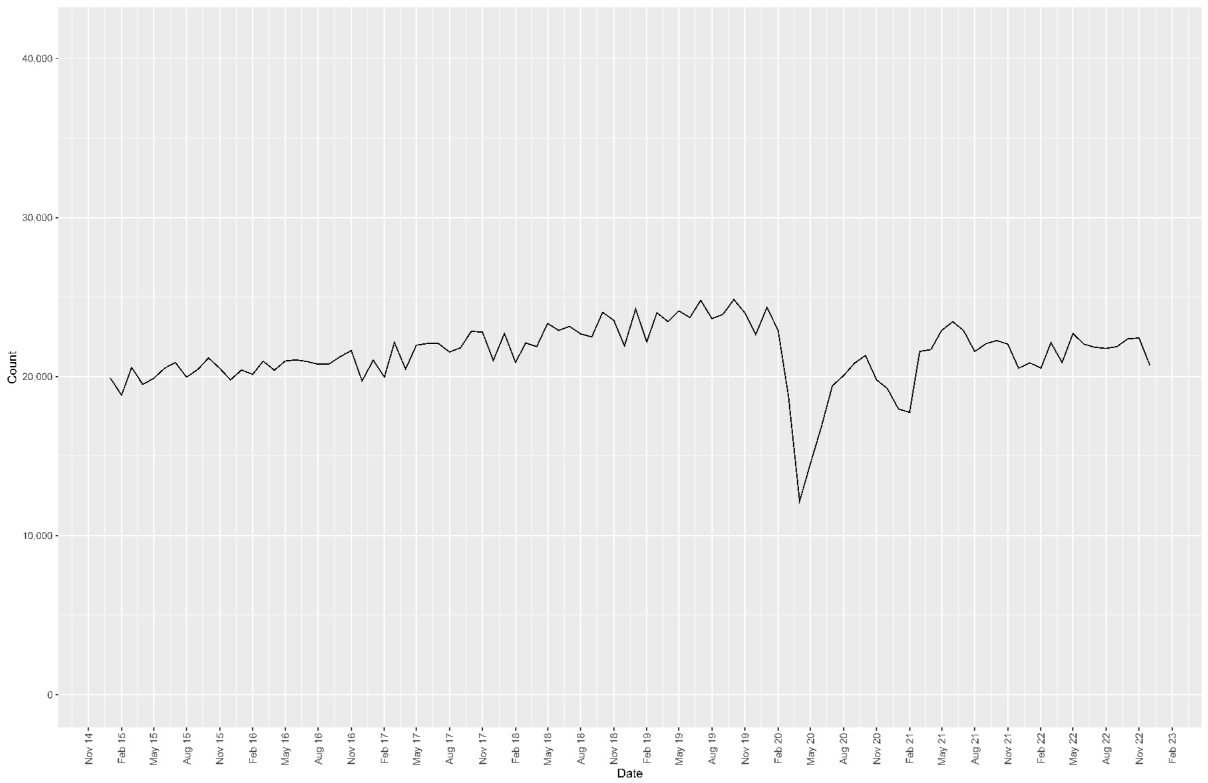

Count
40,000
30,000
20,000
10,000
0
Nov 14
Feb 15
May 15
Aug 15
Nov 15
Feb 16
May 16
Aug 16
Nov 16
Feb 17
May 17
Aug 17
Nov 17
Feb 18
May 18
Aug 18
Nov 18
Feb 19
May 19
Aug 19
Nov 19
Feb 20
May 20
Aug 20
Nov 20
Feb 21
May 21
Aug 21
Nov 21
Feb 22
May 22
Aug 22
Nov 22
Feb 23
Date
